# Global Patterns of Niche Changes in Alien Mammals: Potential Drivers and Significance for Invasion Projections

**DOI:** 10.1111/gcb.70755

**Published:** 2026-03-20

**Authors:** Dino Biancolini, Olivier Broennimann, Antoine Guisan, Carlo Rondinini

**Affiliations:** ^1^ National Research Council of Italy—Institute of Bioeconomy (CNR‐IBE) Rome Italy; ^2^ Global Mammal Assessment Programme, Dipartimento di Biologia e Biotecnologie “Charles Darwin” Sapienza Università di Roma Rome Italy; ^3^ IUCN SSC Invasive Species Specialist Group Rome Italy; ^4^ Department of Ecology and Evolution University of Lausanne Lausanne Switzerland; ^5^ Institute of Earth Surface Dynamics University of Lausanne Lausanne Switzerland

**Keywords:** biotic resistance, climate match, information theory, invasion history, invasion‐risk assessment, life‐history traits, propagule pressure

## Abstract

Biological invasions are a major driver of global change, and prevention is the most effective mitigation strategy. Bioclimatic species distribution models (SDMs) are widely used to estimate invasion risk, assuming that species retain their realized native climatic niches after introduction. We tested this assumption for 194 alien mammal species established across 11 zoogeographic realms, examining realized niche changes, their drivers, and significance for invasion projections. We used a robust ordination framework to compare native and alien niches in 337 species‐by‐realm niche comparisons and quantify niche expansion, the proportion of the alien niche not overlapping with the native niche, and niche unfilling, the proportion of the native niche not overlapping with the alien niche. We then applied Generalized Linear Mixed Models (GLMMs) with multi‐model inference to test how species attributes, invasion history, and environmental context are associated with expansion and unfilling. Additionally, we evaluated the transferability of SDMs built on native presences to receiving regions using multiple metrics and used GLMMs to assess how niche changes may affect it. Niche expansion was rare and modest, whereas niche unfilling was common and pronounced. Niche expansion declined with increasing human disturbance, larger native range size, and introductions within similar communities, but increased with higher introduction effort. Niche unfilling decreased with greater introduction effort and longer residence time, but increased with alien insularity, human disturbance, and native range loss. SDM transferability was generally good, but it declined with niche expansion, as alien presences fell outside native‐like suitable areas, and with unfilling, because suitable areas remained unoccupied under colonization lags. Proactive management can rely on SDMs to anticipate future spread and should prioritize species showing high niche unfilling, indicating substantial spread potential, and any evidence of niche expansion, which makes spread harder to anticipate.

## Graphical abstract

Alien mammals largely retain their realized native climatic niche, yet often show incomplete colonization of receiving zoogeographic regions. Across 337 species‐by‐realm niche comparisons, niche expansion is generally low and concentrated in a few zoogeographic realms, whereas niche unfilling is widespread, indicating large pools of suitable yet unoccupied climatic conditions. Generalized linear mixed models mainly associate niche expansion with higher introduction effort, smaller native ranges, and lower community similarity, and niche unfilling with lower introduction effort, shorter residence time, and higher human disturbance. Species distribution models built on native presences generally transfer well to the receiving regions, but niche expansion reduces performance as alien presences occur outside native‐like suitable conditions, while niche unfilling mainly lowers metrics penalized by suitable but unoccupied areas.

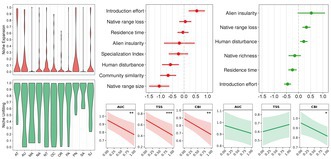

## 1 Introduction

The introduction of alien species is a major driver of global change, with pervasive impacts on ecosystem functioning and human societies (Roy et al. 2023; Schwindt et al. 2023). Prevention and rapid responses are widely recognized as the most effective strategies for mitigating the consequences of biological invasions (Hassall et al. 2025; Sankaran et al. 2023). In this context, proactive management is frequently supported by Species distribution models (SDMs), correlative models that use species presences and environmental variables to identify areas suitable for alien establishment (Guisan et al. 2017; Sankaran et al. 2023). These models are often built on bioclimatic variables, estimating the species' climatic niche, i.e., the range of climatic conditions that allow populations to persist over time (Guisan et al. 2017). However, the transferability of bioclimatic SDMs can be impaired if species' climatic requirements are not conserved after introduction, and SDM projections based on the native climatic niche may then fail to accurately identify suitable climates in new regions (de Andrade et al. 2019; Liu et al. 2020b, 2022; Parravicini et al. 2015).

Over short time scales, niches are expected to remain stable under the niche conservatism hypothesis (Wiens et al. 2010), which underpins numerous climate‐change projection studies about alien (e.g., Biancolini et al. 2024; Louppe et al. 2020) and native species (Biancolini et al. 2025; Newbold 2018). Yet, species introductions can result in mismatches between climatic niches realized in a species' native versus alien ranges, hereafter the “native niche” and the “alien niche” (Broennimann et al. 2012; Guisan et al. 2014). Such mismatches have been reported for alien insects (Bates et al. 2020; Hill et al. 2017), freshwater mollusks (Mahapatra et al. 2025), and marine fishes (Parravicini et al. 2015), whereas niche conservatism has been reported for alien plants (Cao Pinna et al. 2025; Guisan et al. 2014; Petitpierre et al. 2012) and terrestrial vertebrates (Broennimann et al. 2021; Du et al. 2024; Liu et al. 2017; Strubbe et al. 2013, 2015).

To quantify these mismatches in a way that is directly relevant for proactive management, niche changes during alien spread can be categorized into three components: “niche unfilling”, the proportion of the native niche not realized in the receiving region; “niche expansion”, the proportion of the alien niche not realized in the native region; and “niche stability”, the overlap between the two niches (Guisan et al. 2014; Petitpierre et al. 2012). Niche expansion and unfilling are particularly relevant for invasion‐risk assessments based on native‐range SDMs (Liu et al. 2022; Pili et al. 2020). Niche expansion indicates the occupation of conditions that were available but unoccupied in the native region and, consequently, causes alien presences to occur in areas classified as unsuitable by native‐range SDMs (Liu et al. 2020b, 2022). Niche unfilling instead reflects incomplete spread after introduction within the receiving region, and therefore, leaves areas classified as suitable still unoccupied there (Liu et al. 2020b, 2022; Pili et al. 2020). Quantifying both components is therefore necessary to interpret native‐range SDMs and their uncertainty when projected to receiving regions (Liu et al. 2020b, 2022).

In this context, niche changes can arise from multiple processes linked to species attributes, invasion history, and the environmental characteristics of the alien range (Li et al. 2014; Strubbe et al. 2013, 2015), and identifying these drivers is essential to improve invasion‐risk assessments (Guisan et al. 2014; Li et al. 2014; Strubbe et al. 2013, 2015). Species attributes influence how alien populations occupy climatic conditions in the receiving region. Fast life histories and high dispersal ability facilitate colonization by generating new dispersing individuals rapidly and increasing the capacity of alien populations to reach suitable conditions by their own movement (Capellini et al. 2015; Wilson et al. 2009). Furthermore, broad diet and habitat breadth may increase the likelihood that species can occupy new and native‐like suitable conditions in the receiving region (Biancolini and Rondinini 2025; Dyer et al. 2016; Li et al. 2014). By contrast, species with broad native ranges, already occupying a wide climatic spectrum, may have limited scope to expand into new climates and may also show greater difficulty in filling their native niche in the receiving region (Bates et al. 2020; Liu et al. 2020a). However, anthropogenic range loss can truncate the native climatic niche (Chevalier et al. 2021; Faurby and Araújo 2018), potentially distorting comparisons between native and alien niches. Invasion history further shapes the climatic space that alien populations can reach and occupy. The magnitude and spatial pattern of human‐assisted dispersal, understood here as propagule pressure arising from multiple introduction locations and pathways, determine the diversity of climatic conditions to which alien populations are initially exposed (Duncan et al. 2001; Lockwood et al. 2009). Starting from introduction locations, colonization usually proceeds through progressive spread, requiring extended time spans, and recently established populations are unlikely to have reached the full range of climates available in the receiving region (Broennimann et al. 2014; Li et al. 2014; Rouget et al. 2016). However, alien species are frequently introduced to islands (Stroud 2021), where limited climatic heterogeneity and strong geographical isolation can restrict the realization of the alien niche (Alexander and Edwards 2010). Finally, the environmental conditions in the alien range can constrain or facilitate niche changes. When introductions occur within the same zoogeographic regions as the native range, alien populations encounter communities with similar composition (Holt et al. 2013), posing native‐like biotic constraints on the alien niche, whereas introductions into different regions may benefit from enemy release and lower biotic constraints (Crego et al. 2018; Gallagher et al. 2010). In both cases, high native‐mammal richness may limit the available environmental space for alien populations and pose high biotic resistance (Beaury et al. 2020; Gotelli et al. 2010), whereas human disturbance can weaken such resistance (Gallardo et al. 2015; Jeschke and Heger 2018).

Large‐scale studies have significantly advanced our understanding of alien niche dynamics for major groups, including plants (Petitpierre et al. 2012), birds (Cardador and Blackburn 2020), and herpetofauna (Li et al. 2014). In contrast, a comprehensive global study of alien mammals is currently lacking. Mammals share a long history of co‐existence with humans (Biancolini et al. 2021; Blackburn et al. 2017; Long 2003; Seebens et al. 2025), being intentionally and accidentally introduced for multiple purposes since prehistoric times (Biancolini et al. 2021; Long 2003; Seebens et al. 2025). At least 230 mammals have established self‐sustaining alien populations on all continents except Antarctica, many causing impacts through diverse mechanisms (Biancolini et al. 2021; Blackburn et al. 2017; Long 2003). A taxon‐wide global analysis of potential niche changes and their drivers could provide useful insights to guide alien mammal management under global change.

Here, we investigated global patterns of climatic niche changes in established alien mammals, their potential drivers, and significance for SDM projections. We addressed three main questions: (1) How widespread and important are climatic niche changes for alien mammals? (2) What species and environmental factors might drive these changes? (3) What are the implications for anticipating the spread of alien mammals? To address these questions, we (1) applied a robust framework to compare native and alien climatic niches, (2) used Generalized Linear Mixed Models to identify which factors are correlated with niche changes, and (3) investigated how niche changes could affect the transferability of native‐range SDMs to the receiving regions.

We expected greater niche expansion and lower niche unfilling for factors that increase the production, movement, and establishment of dispersers across the available climatic conditions. Specifically, higher dispersal ability (1) and a faster life history (2) should promote expansion while reducing unfilling, and lower ecological specialization, understood as broader use of habitats and food types (3), should show the same pattern because generalist species are more likely to cope with diverse environments. In terms of invasion history, higher human‐assisted dispersal (4) and longer residence time (5) should also increase expansion and decrease unfilling by increasing the probability that alien populations access and persist in a wide range of conditions. Finally, stronger human disturbance (6) should favor expansion and reduce unfilling by weakening biotic resistance. We also expected native range loss (7) to show these same directions, inflating apparent expansion and reducing unfilling because local extinction truncates the native niche. Conversely, we expected lower niche expansion and higher unfilling when the native range is broad or when the alien range is intrinsically constrained. Thus, native range size (8) should be negatively related to expansion and positively to unfilling, because species already occupying broad native climatic space have limited scope to occupy new climates but more native‐like suitable climates that may remain unoccupied. Alien insularity (9) should likewise limit expansion and increase unfilling due to the narrow climatic spectrum and strong geographic isolation of islands. We also anticipated that high native‐mammal richness (10) would constrain expansion and maintain higher unfilling through stronger biotic resistance. Finally, we expected greater community similarity between native and alien ranges (11) to reduce both niche expansion and unfilling, because introductions into biogeographically similar regions expose alien populations to native‐like biotic constraints.

Regarding native‐range SDM transferability, we hypothesized that it would be generally good but would decline with niche expansion and, differently, with unfilling, across two metric classes: (1) discrimination and classification metrics that compare presences to pseudo‐absences, and (2) presence‐only metrics that quantify the concentration of presences in high‐suitability classes. Under niche expansion, alien presences should occur in climates not occupied in the native range and projected as unsuitable by native‐range SDMs, reducing performance across both classes. Under niche unfilling, native‐range SDMs should classify climates included in the native niche as suitable, but these climates may remain only partially occupied, so metrics relying on pseudo‐absences or on occupancy of high‐suitability classes should decline, whereas metrics reflecting correct identification of presences should remain high.

## 2 Methods

### 2.1 Study and Background Areas

We conducted all analyses at 5 arc‐min resolution (~10 km at the equator) across the 11 zoogeographic realms (Holt et al. 2013). For niche analyses and SDMs, we sampled pseudo‐absences within background areas intended to represent the climatic conditions accessible to the species (Guisan et al. 2014; Rausell‐Moreno et al. 2025). We delineated species‐specific native and alien backgrounds using the mammal zoogeographic regions (Holt et al. 2013) that overlapped each species' native and alien ranges (Table S1.1 and S1.2). These 34 zoogeographic regions reflect major dispersal barriers and shared mammalian evolutionary histories, providing ecologically meaningful background boundaries for mammals (Biancolini et al. 2024; Biancolini and Rondinini 2025).

### 2.2 Species Distribution and Climate Data

We obtained alien range maps from the global Distribution of Alien Mammals (DAMA) database, which maps established alien populations (Biancolini et al. 2021). For each species, we grouped alien polygons by the realm of introduction, producing 388 “realm‐level” alien ranges (Biancolini and Rondinini 2025). We obtained native range maps from the IUCN Red List (IUCN 2020). When native and alien ranges overlapped, we assigned overlapping portions to the alien range (Biancolini and Rondinini 2025) because DAMA ranges were explicitly designed to include only alien populations and exclude native ones, whereas IUCN ranges may include alien, uncertain, or misclassified areas (IUCN 2019). We converted native and alien ranges to 5 arc‐min cells that received a value of one (Biancolini et al. 2024; Biancolini and Rondinini 2025) (Appendix S1: Section 1.1). Among the 230 DAMA species, 194 met the minimum of five presences per range required for niche analyses (Di Cola et al. 2017); the same threshold was met by 337/388 realm‐level alien ranges. Median presences were 39,207 (SD = 192,847.5) in the native ranges and 291 (SD = 35,016.69) in the alien ranges (Tables S1.1‐S1.3; Figure S1.1).

To characterize climatic niches and build native‐range SDMs, we used the 19 bioclimatic variables from the CHELSA 2.1 dataset (Karger et al. 2017), aggregated from 30 arc‐seconds to 5 arc‐min resolution (Appendix S1: Section 1.2). Climate is a key constraint for terrestrial vertebrates' distributions at broad scales (Li et al. 2016; Liu et al. 2020b), and bioclimatic variables have been frequently used to analyze (e.g., Arlé et al. 2024; Broennimann et al. 2021; Di Marco et al. 2021; Strubbe et al. 2015) and model the niche (e.g., Biancolini et al. 2024; Biancolini and Rondinini 2025; Newbold 2018; Strubbe et al. 2015) of mammals.

### 2.3 Analysis of Niche Changes

For each of the 194 established alien mammals, we compared the native niche with the alien niche(s) realized within the receiving realm(s). Because some species were introduced to multiple realms, this resulted in 337 niche comparisons. We derived presences from native and alien range maps (Section 2.2). To generate pseudo‐absences, we converted native and alien backgrounds to 5‐arc‐min grids, assigning their cells a value of zero and removing any cell overlapping presences (Appendix S1: Section 1.2). Using the *ecospat* R package (Broennimann et al. 2024; Di Cola et al. 2017), we conducted a principal component analysis (PCA) separately for each species‐by‐realm comparison on bioclimatic values extracted from presences and pseudo‐absences in both native and alien backgrounds, defining a climatic space on the first two PCA axes (Broennimann et al. 2012; Di Cola et al. 2017) that captured > 75% of the total variance. We then applied kernel smoothing to estimate species presence densities on a 100 × 100 gridded climatic space, enabling niche comparisons despite differences in range and background extent (Broennimann et al. 2012; Di Cola et al. 2017). We quantified niche expansion (portion of the alien niche not occupied in the native range), niche stability (overlap between native and alien niches), and niche unfilling (portion of the native niche not occupied in the alien range) (Petitpierre et al. 2012). All metrics range from 0 to 1; niche expansion and stability partition the alien niche, whereas niche unfilling refers to the native niche (Petitpierre et al. 2012).

To ensure meaningful niche comparisons, we restricted analyses to climatic conditions shared between native and alien backgrounds, excluding novel climates (Guisan et al. 2012, 2014). This design isolates differences in niche realization within comparable climatic space, so observed niche changes are not conflated with background climatic differences and can be interpreted in relation to species attributes, invasion history, and environmental conditions (Guisan et al. 2012, 2014). We quantified the prevalence of novel climates using the Extrapolation Detection tool (Mesgaran et al. 2014) implemented in *ecospat* (Appendix S1: Section 1.2.1), which identifies areas where alien‐background climates fall outside the range or the multivariate structure of climates observed in the native background. Climate novelty exceeded 5% of cells in only 19/337 niche comparisons (Table S1.4, Figure S1.2).

Finally, we mapped spatial patterns of niche metrics in QGIS 3.34 (https://qgis.org/) by binarizing expansion and unfilling at ≥ 0.1 (1) versus < 0.1 (0), and defining stability as 1 when expansion < 0.1 and niche filling as 1 when unfilling < 0.1 (Petitpierre et al. 2012).

### 2.4 Drivers of Niche Changes

To investigate potential drivers of niche changes in established alien mammals, we compiled data on species attributes, invasion history, and environmental characteristics of the alien ranges (Table 1, Appendix S1: Section 1.3, Figures S1.3–S1.13). Nine of the 11 considered variables were prepared by or adapted from Biancolini and Rondinini (2025).

**TABLE 1:** Descriptions and expected effects of the considered drivers of niche change of established alien mammals, categorized under three classes: environmental conditions (E), invasion history (H), and species attributes (S). Drivers under “E” and “H” are calculated for each species within each zoogeographic realm (Holt et al. 2013). Further information about variables statistics is provided in Appendix S1.

Driver	Class	Metric	Description	Expansion	Unfilling	Source
Native range size	S	km^2^	The geographical extent of a species' native distribution	Negative	Positive	IUCN (2020)
Native range loss	S	Count	The number of cells lost by the native range due to human pressures	Positive	Negative	Faurby et al. (2018)
Dispersal	S	Km	The median distance traveled by a species between the birth and breeding areas	Positive	Negative	Soria et al. (2021)
Fast‐growth index	S	Number	The potential of a species for rapid population growth^a^	Positive	Negative	Soria et al. (2021)
Specialization index	S	Number	A measure combining the variety of consumed food types and frequented habitat types^a^	Negative	Positive	Kissling et al. (2014), IUCN (2020)
Introduction effort	H	Count	A measure of the magnitude of human‐assisted dispersal based on distinct introduction locations and pathways.^a^	Positive	Negative	Long (2003), Biancolini et al. (2021), Blackburn et al. (2017)
Residence time	H	Years	Time since the first recorded introduction^a^	Positive	Negative	Biancolini et al. (2021)
Alien insularity	H	Binary	Whether the alien range is confined to islands (1 = yes, 0 = no)^a^	Negative	Positive	Biancolini et al. (2021)
Human disturbance	E	Median	The degree of human alteration of the environment^a^	Positive	Negative	Mu et al. (2022)
Native‐mammal richness	E	Median	The number of native mammal species^a^	Negative	Positive	IUCN (2020); Lumbierres et al. (2022)
Community similarity	E	Proportion	The proportion of mammal zoogeographic regions common to the alien and native backgrounds^b^	Negative	Negative	Holt et al. (2013)

^a^ Prepared by Biancolini and Rondinini (2025).

^b^ Adapted from Biancolini and Rondinini (2025).

#### 2.4.1 Species Attributes

We used COMBINE trait data (Soria et al. 2021) to position species along the fast‐slow life‐history continuum (Stott et al. 2024; Strubbe et al. 2013). A scaled PCA was run on eight traits measured as durations or rates of survival, development, and reproduction (Stott et al. 2024). The first PCA axis accounted for 71.87% of the variance and provided a fast‐growth index (Biancolini and Rondinini 2025), with higher values indicating fast‐strategists (e.g., early maturation, high reproductive output) and lower values indicating slow strategists (e.g., delayed reproduction, extended lifespan) (Figure S1.3). We quantified species' autonomous access to suitable conditions using COMBINE median dispersal distance (Figure S1.4).

We represented native niche breadth with native range size (IUCN 2020; Table 1, Figure S1.5), the number of level 2 habitat types (IUCN 2020), and the number of consumed food types (Kissling et al. 2014). Habitat and diet breadth were combined into a specialization index, ln[100/(foods × habitat types)] (Sekercioglu 2011), ranging from generalists (negative) to specialists (positive) (Table 1, Figure S1.6).

We estimated native range loss as the number of cells present in the Phylacine “present natural” range but absent from current native ranges (Faurby et al. 2018), capturing anthropogenic contraction (Table 1, Figure S1.7).

#### 2.4.2 Invasion History

For each species in each realm, introduction locations were obtained from published sources and compiled datasets (Biancolini et al. 2021; Blackburn et al. 2017; Long 2003), following established methodologies (Capellini et al. 2015; Duncan et al. 2001; Tedeschi, Lenzner, Schertler, Wessely, et al. 2025), and introduction pathways were extracted from DAMA. Introduction effort was calculated as sqrt(*L* × *P*), where *L* is the number of distinct introduction locations and *P* the number of pathways (Biancolini and Rondinini 2025), so the product rewards species with multiple locations and pathways, while the square root attenuates extremes and stabilizes variance (Zuur et al. 2007, 2009) (Table 1; Figure S1.8). Residence time was calculated by subtracting the first year of introduction from DAMA for a species in a given realm from 2021, the database publication year (Table 1, Figure S1.9). Alien insularity was defined as alien ranges within a realm consisting exclusively of polygons labeled as “Island” or “Archipelago” in DAMA (Table 1, Figure S1.10).

#### 2.4.3 Environmental Characteristics

To characterize the alien ranges, spatial data on human disturbance and native‐mammal richness (Table 1, Figures S1.11 and S1.12) were obtained from the Human Footprint dataset (Mu et al. 2022) and from mammal Area of Habitat (Lumbierres et al. 2022) within native range maps (IUCN 2020), respectively. For each alien range, we used the median of both variables, providing a measure of central tendency. We quantified community similarity as the proportion of zoogeographic regions (Holt et al. 2013) composing the alien background that also occur in the native one (Table 1, Figure S1.13), a proxy for the presence of natural enemies (Biancolini and Rondinini 2025).

### 2.5 Generalized Linear Mixed Models of Niche Change

Collinearity can inflate standard errors, mask effects, and increase overfitting risk (Zuur et al. 2007, 2009). We assessed collinearity among variables using the Variance Inflation Factor (VIF) with the “vifstep” function from the *usdm* R package (Naimi 2023). All variables had VIF < 3 (Table S1.5), indicating low collinearity (Zuur et al. 2007, 2009).

Shapiro–Wilk tests in R indicated that niche expansion and unfilling were skewed (Appendix S1: Section 1.4.1). Because niche expansion was strongly right‐skewed with many near‐zero values, we converted it to a binary response to allow binomial modelling. We used *k*‐means clustering (*k* = 2) to obtain a data‐driven threshold (Mucherino et al. 2009; Zuur et al. 2007) and defined the outcome as 1 for values > 0.035, corresponding to the centroid of the denser cluster near zero (Appendix S1: Section 1.4). For niche unfilling, we applied a square‐root transformation (Zuur et al. 2007, 2009), ensured transformed values remained within (0, 1) (Appendix S1: Section 1.4), and modeled them with beta regression (Figure S1.14).

We built Generalized Linear Mixed Models (GLMMs) using the *glmmTMB* R package (Brooks et al. 2017, 2024). Using the compiled dataset of 337 species‐by‐realm niche metrics, we fitted global models for niche expansion and unfilling including the 11 selected variables as independent fixed effects, without interaction terms (Table 1). We used a binomial family for niche expansion and a beta family for niche unfilling. Because baseline response levels may differ among recipient realms and among taxonomic orders, and because species occurring in multiple realms can introduce pseudo‐replication, we included random intercepts (Bolker et al. 2009; Zuur et al. 2009) for receiving realm (Biancolini and Rondinini 2025; Li et al. 2014) and taxonomic order (Dembitzer et al. 2022).

To avoid overly complex models, we considered the common rule‐of‐thumb of at least 10 observations per variable (Bolker et al. 2009; Harrell 2015), which was satisfied (337/11 = 30.64). We further assessed the random‐effects structure by checking for singularity (Bolker et al. 2009) using the “check_singularity” function from the *performance* R package (Lüdecke et al. 2025). The niche‐expansion global model exhibited singularity, indicating that the random‐effects structure was over‐parameterized. We therefore simplified it by using species identity as a single random effect to address pseudo‐replication. We tested for phylogenetic signal in niche expansion by estimating Pagel's *λ* (Münkemüller et al. 2012) using a maximum clade credibility tree computed from a posterior sample of Phylacine trees (Appendix S1: Section 1.2.2). Pagel's *λ* was 0.090 with a *p*‐value of 0.129, confirming that a taxonomic random effect was unnecessary. The niche‐unfilling model with Realm and Order random effects showed no singularity.

To improve linearity and homoscedasticity (Zuur et al. 2007, 2009), we transformed fixed variables (Appendix S1: Section 1.4). For the niche‐unfilling model, we Yeo‐Johnson transformed (Peterson 2023a, 2023b) native range loss and introduction effort, and log‐transformed dispersal, native range size, native‐mammal richness, residence time, and human disturbance. For the niche‐expansion model, we Yeo‐Johnson transformed human disturbance, introduction effort, native range loss, and native‐mammal richness, Box–Cox transformed (Peterson 2023a) native range size, and log‐transformed dispersal and residence time. For each response, we fitted models with raw versus transformed variables and compared them using AICc and effect plots (Appendix S1: Section 1.4.1). The transformed models consistently outperformed and produced more linear effect profiles (Appendix S1: Section 1.4.1, Figures S1.15 and S1.16). We also standardized all non‐binary variables to allow comparisons across different measurement scales (Zuur et al. 2007, 2009).

We adopted an information‐theoretic framework based on the small‐sample‐corrected Akaike Information Criterion (AICc) to perform multi‐model inference (Akaike 1973; Burnham and Anderson 2002). Starting from a global model, we constructed a candidate set comprising all ecologically informed additive subsets of the global fixed effects (Burnham and Anderson 2002). We used this full‐subsets approach because there was no strong prior support for any particular combination of variables, and we aimed to evaluate multiple ecological hypotheses. We ranked candidate models using AICc, which is appropriate when the sample size‐to‐parameter ratio is < 40 (Burnham and Anderson 2002). We generated the candidate set and AICc values using the “dredge” function from the *MuMIn* R package (Bartoń 2024), which systematically generates all candidate models derived from the global model. We defined a confidence set as models with substantial support (ΔAICc < 4) and excluded more complex nested variants of simpler models to avoid redundancy and prevent inflating support for unnecessary parameters (Burnham and Anderson 2002; Grueber et al. 2011; Symonds and Moussalli 2011). Within this confidence set, we performed full model averaging using Akaike weights to obtain model‐averaged parameter estimates, unconditional standard errors, and 95% confidence intervals (Burnham and Anderson 2002; Grueber et al. 2011). Because the all‐subsets approach entails model selection uncertainty, we treated coefficients of variables absent from a given model as zero; this full‐averaging approach reduces model selection bias by limiting the tendency for estimates to be inflated away from zero (Bolker et al. 2009; Grueber et al. 2011; Symonds and Moussalli 2011). Model averaging reduces reliance on a single “best” model and provides effect‐size estimates that incorporate model‐selection uncertainty, while AICc penalizes model complexity relative to sample size, reducing the risk of overfitting (Burnham and Anderson 2002; Bolker et al. 2009; Grueber et al. 2011).

We evaluated GLMM fit and assumptions using the *DHARMa* R package (Hartig et al. 2024), which generates Q–Q plots and tests for residual uniformity (Kolmogorov–Smirnov), dispersion, and outliers, and produces residual plots of observed versus expected values (Appendix S1: Section 1.6).

### 2.6 Transferability of Native‐range Species Distribution Models

We used the native‐range SDMs built by Biancolini and Rondinini (2025) for 193 species with at least 20 native presences (Guisan et al. 2017; Table S1.1). Presences were paired with pseudo‐absences sampled at a 1:1 ratio within the native background (defined by zoogeographic regions) (Rausell‐Moreno et al. 2025) to generate five pseudo‐absence datasets per species. To reduce multicollinearity and maximize model transferability, the 19 bioclimatic variables from CHELSA were reduced to the first four PCA axes (Liu et al. 2020b; Petitpierre et al. 2017; Zhang et al. 2023), containing > 90% of the total variance (Biancolini and Rondinini 2025). Native‐range SDMs were built with these data and Generalized Boosted Models with 200 trees, implemented in the *biomod2* R package (Thuiller et al. 2023), a low‐complexity setting enhancing transferability (Biancolini et al. 2024; Elith et al. 2010). Three replicates per pseudo‐absence dataset were run, with 15 runs per species. Occurrences were split into calibration (70%) and evaluation (30%) sub‐datasets, and SDM performance was evaluated by calculating the Area Under the receiver operating characteristic Curve (AUC). AUC measures discrimination between presences and pseudo‐absences from 0.5 (random) to 1 (perfect) (Guisan et al. 2017). Only replicates with an AUC > 0.7 were retained for the final averaged projection (Guisan et al. 2017). Following best practice (Sillero et al. 2021), SDMs were also evaluated with maximized True Skill Statistic (TSS) and the Continuous Boyce Index (CBI; Hirzel et al. 2006). TSS combines Sensitivity (true positive rate) and Specificity (true negative rate), ranges from −1 to +1 (higher indicates better discrimination), and it is computed across thresholds from 0 to 1, with the maximum taken as performance (Guisan et al. 2017). In contrast, CBI is a presence‐only metric comparing observed versus random presence frequencies across suitability classes, ranges from −1 to +1, and higher values indicate a strong concentration of presences in high‐suitability classes (Di Cola et al. 2017; Hirzel et al. 2006). Native‐range SDMs performed well in native backgrounds (median AUC = 0.949, TSS = 0.794, and CBI = 0.944; Biancolini and Rondinini 2025). Novel climates from SDM projections were excluded using the *biomod2* clamping mask (Fitzpatrick and Hargrove 2009; Thuiller et al. 2023) so that SDM extrapolation beyond the native climatic space did not influence transferability evaluations (Fitzpatrick and Hargrove 2009; Strubbe et al. 2013).

To assess native‐range SDM transferability to alien backgrounds, we used realm‐level alien presences as an independent evaluation dataset (C. Liu et al. 2020b; Strubbe et al. 2013, 2015) to calculate 337 species‐by‐realm AUC, TSS, Sensitivity, Specificity, and CBI (Appendix S1: Section 1.5). We sampled pseudo‐absences at random from unoccupied cells using the “randomPoints” function from the *dismo* R package (Hijmans et al. 2024). Following Rausell‐Moreno et al. (2025), who found that background sampling proportional to species presences or to background area yields comparable performance, we implemented a tiered fallback to ensure sufficient pseudo‐absence availability across backgrounds (Biancolini and Rondinini 2025). We first paired alien presences with pseudo‐absences sampled at a 1:1 ratio within the alien background; when too few unoccupied cells were available, we instead targeted a sample size equal to 5% of all background cells (41 cases). When this target also could not be met due to limited unoccupied cells, we sampled pseudo‐absences at a 1:1 ratio within all realms (6 cases). We then calculated AUC, TSS, Sensitivity, and Specificity using the *enmSdmX* R package (Smith 2024), while for CBI, we used *ecospat*.

To investigate the relationship between native‐range SDM transferability and niche changes, we constructed mixed models using the five performance metrics as response variables and niche expansion and unfilling as fixed effects. We included the receiving realm as a random effect to account for pseudo‐replication and native sample size as an additional random effect to control for the known influence of data availability on SDM transferability (C. Liu et al. 2022). We created the latter by converting native range size into five percentile‐based classes (Bolker et al. 2009): very small (0–20th), small (20–40th), medium (40–60th), large (60–80th), and very large (80–100th). We selected the most appropriate transformation and modeling approach for each metric (Appendix S1: Section 1.5.1). For AUC, TSS, and CBI, we applied the arcsine square root transformation (Zuur et al. 2007, 2009) to approximate a Gaussian distribution (Figure S1.17) and subsequently fitted linear mixed models. For Sensitivity and Specificity, we binarized the continuous scores at their respective first quartile and built binomial GLMMs. We assessed random‐effects structures for singularity (Lüdecke et al. 2025). All models were non‐singular except the Sensitivity model, which was consistently singular across alternative random effects (species identity, receiving realm, and native sample size); we therefore removed random effects from it. Finally, we evaluated model fit, residual behavior, and assumption validity using *DHARMa* (Appendix S1: Section 1.6).

## 3 Results

### 3.1 Patterns of Niche Changes

**FIGURE 1:**
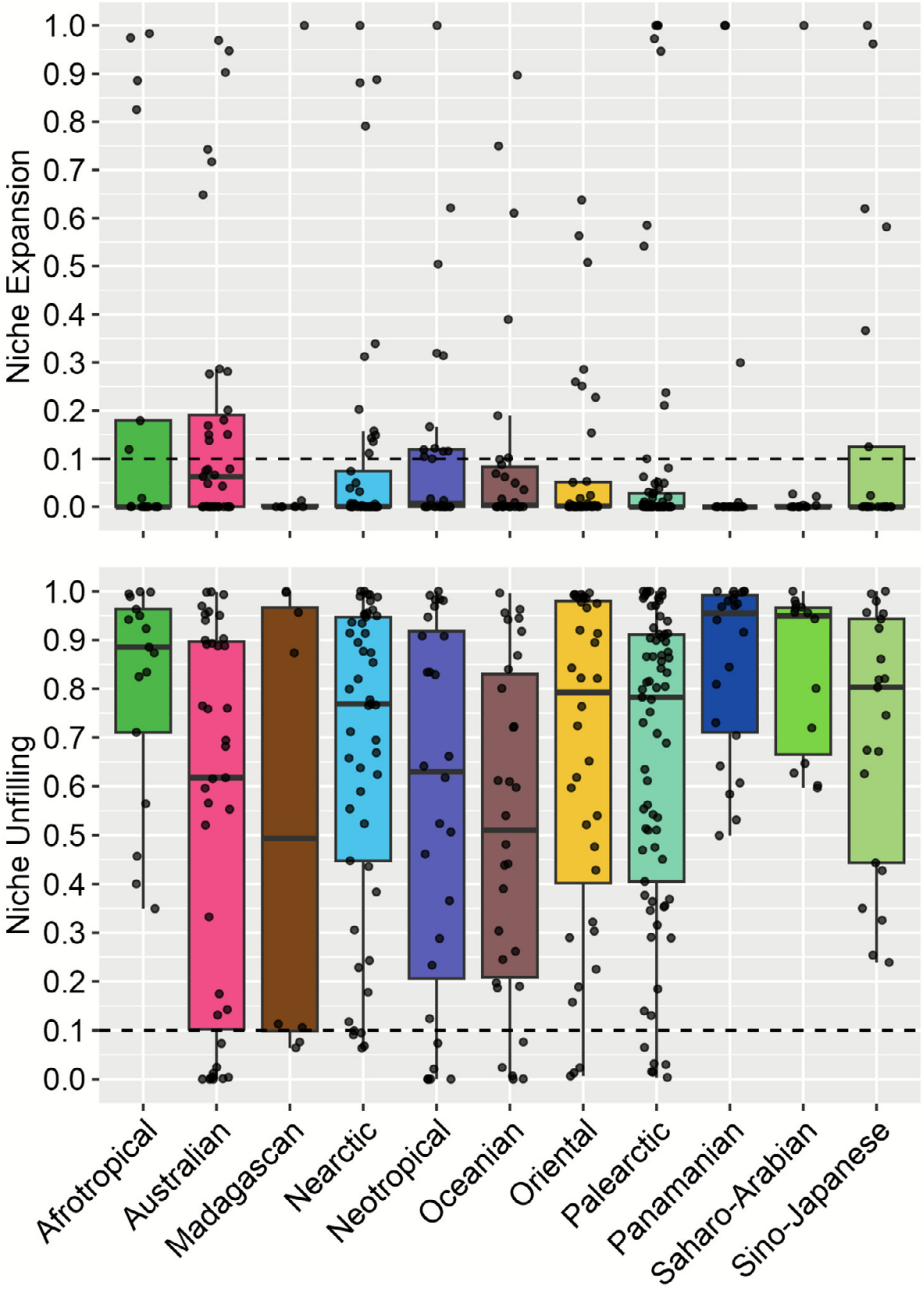
Boxplots showing niche expansion (top) and niche unfilling (bottom) of alien mammals across zoogeographic realms, with a dashed line indicating values ≥ 0.1 following Petitpierre et al. (2012).

We found realm‐specific patterns in niche expansion and unfilling (Figure 1, Table S2.1). Median niche expansion was generally low (0.0002, SD = 0.268), with the Australian realm showing a higher median (0.063, 0.292), while other realms had medians close to zero (Figure 1). Niche stability was the complement of expansion and therefore showed the opposite pattern across realms (Table S2.1). By contrast, median niche unfilling was generally high (0.766, 0.330), peaking in the Panamanian (0.95, 0.18), Saharo‐Arabian (0.95, 0.16) and Afrotropical (0.85, 0.22) realms, and was more moderate in the Madagascan (0.49, 0.46), Oceanian (0.51, 0.34) and Australian (0.62, 0.39) (Figure 1, Table S2.1).

**FIGURE 2:**
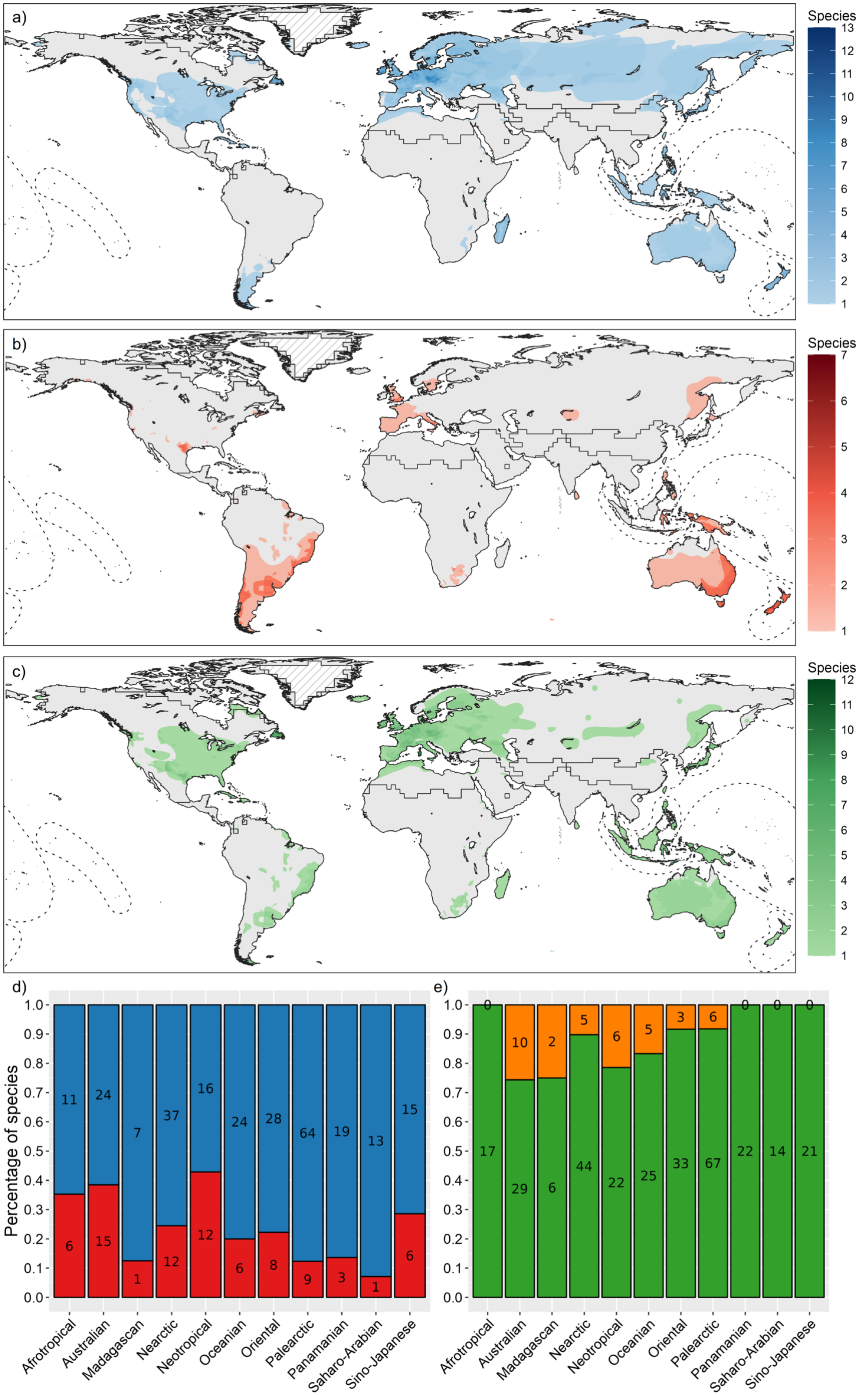
(a–c) Maps showing the number of alien species ranges with niche stability (expansion < 0.1; a), niche expansion (expansion ≥ 0.1; b), and niche unfilling (unfilling ≥ 0.1; c) across zoogeographic realms. (d) Barplots of the count and proportion of ranges classified as stable (expansion < 0.1; blue) versus expanding (expansion ≥ 0.1; red). (e) Barplots of the count and proportion of ranges classified as unfilling (unfilling ≥ 0.1; green) versus filling (unfilling < 0.1; orange). Continuous niche metrics were binarized at a threshold of 0.1, assigning one when ≥ 0.1 and zero otherwise, with complementary states (stability and filling) defined accordingly, following Petitpierre et al. (2012).

Niche expansion ≥ 0.1 occurred in 79/337 alien ranges (23.44%) and 54/194 species (27.83%) in at least one realm (Figures 1 and 2, Table S2.1). The highest percentage of species with expansion ≥ 0.1 was in the Neotropical realm (12/28 species, 42.86%), followed by the Australian (15/39, 38.46%) and the Afrotropical (6/17, 35.29%). The highest percentage of species with stable niches was in the Saharo‐Arabian (13/14, 92.86%), followed by the Palearctic (64/73, 87.67%) and the Madagascan (7/8, 87.50%). Niche unfilling ≥ 0.1 occurred in 300/337 alien ranges (89.02%) and 185/194 species (96.36%) in at least one realm (Figures 1 and 2, Table S2.1), with 100% of species showing niche unfilling ≥ 0.1 in the Afrotropical (17 species), Panamanian (22), Saharo‐Arabian (14) realms and Sino‐Japanese (21).

### 3.2 Drivers of Niche Changes

We produced 2048 candidate GLMMs for niche expansion and unfilling, of which 114 and 66, respectively, were non‐nested. The best‐supported non‐nested models (ΔAICc < 4) were six for niche expansion and three for unfilling (Tables S2.3–S2.8). *DHARMa* diagnostics indicated no major issues for either the global models or the best‐supported models (Figures S2.2–S2.12).

**FIGURE 3:**
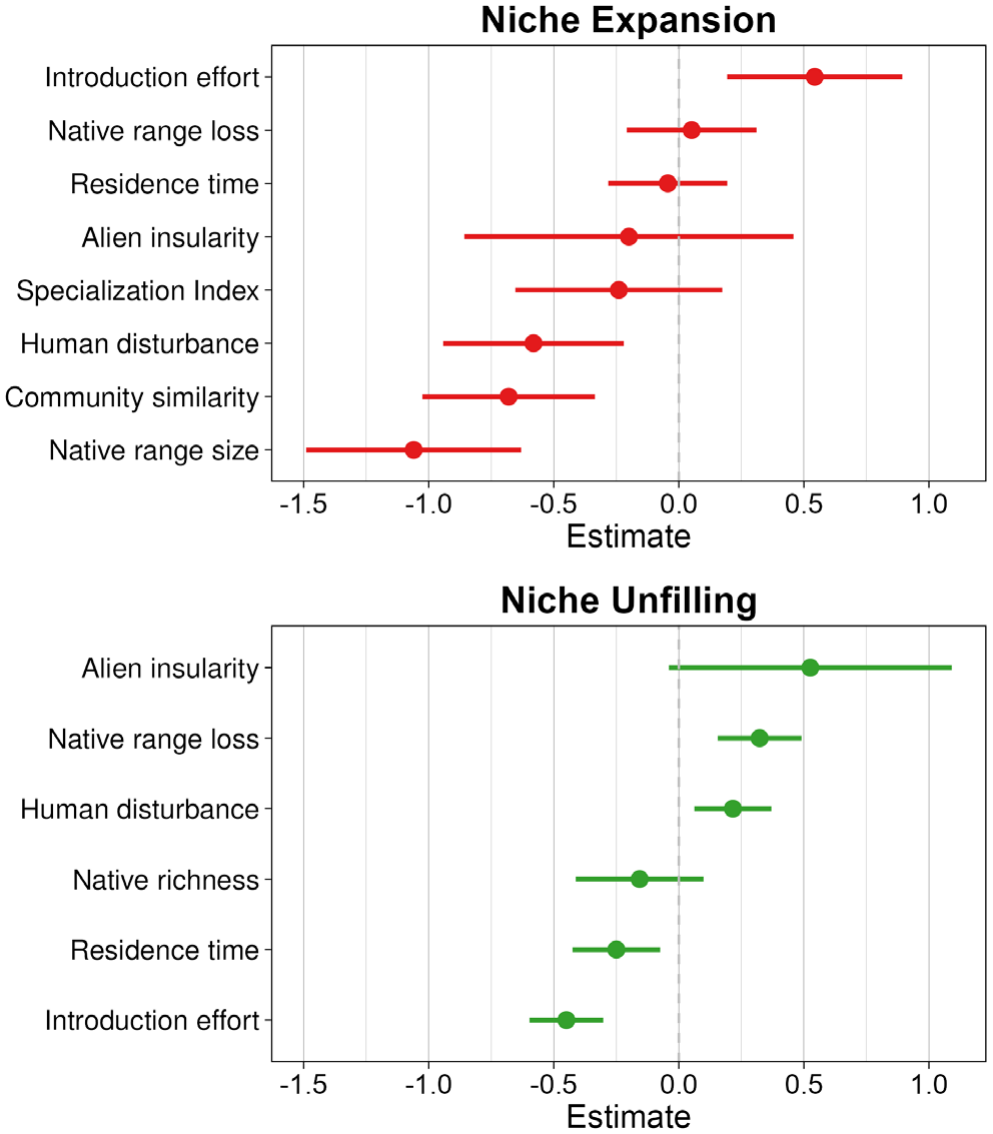
Effect estimates of niche‐change drivers on niche expansion and unfilling in GLMMs. Points indicate effect estimates and horizontal bars show 95% confidence intervals.

For niche expansion, the fully averaged model highlighted a core group of variables whose 95% confidence intervals excluded zero (Figure 3, Figure S2.18 and Table S2.9). In the averaged model, niche expansion was negatively associated with native range size, community similarity, and human disturbance, and positively associated with introduction effort. In all these cases, confidence intervals lay entirely on one side of zero, indicating supported effect directions. By contrast, alien insularity, ecological specialization, native range loss, and residence time all had confidence intervals that overlapped zero, in some cases almost symmetrically around zero, indicating weak support.

For niche unfilling, the fully averaged model also identified a set of variables whose 95% confidence intervals excluded zero (Figure 3, Figure S2.19 and Table S2.10). Unfilling increased with human disturbance and native range loss and decreased with introduction effort and residence time, with confidence intervals that lay entirely on one side of zero. Alien insularity and native richness had confidence intervals that only marginally overlapped zero, with estimates suggesting positive and negative associations, respectively.

### 3.3 Transferability of Native‐range Species Distribution Models

Native‐range SDMs transferred well to alien backgrounds, although performance varied among metrics (Table S2.2, Figure S2.1): AUC (median = 0.660, SD = 0.241), TSS (0.411, 0.306), CBI (0.267, 0.553), Sensitivity (98.100%, 19.277%), and Specificity (62.800%, 32.862%).

**FIGURE 4:**
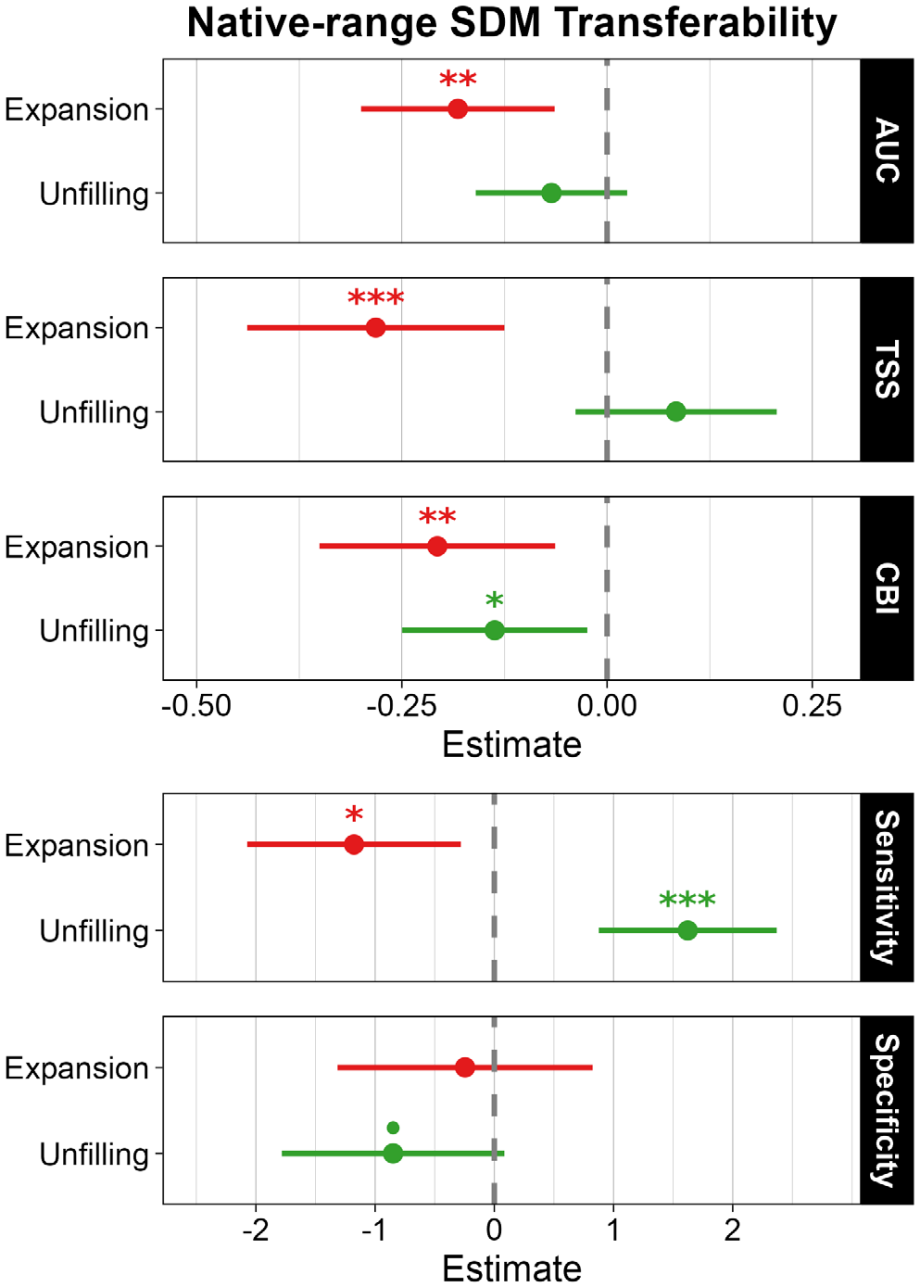
Effect estimates of niche expansion and unfilling on native‐range SDM transferability in (G)LMMs. Points indicate effect estimates and horizontal bars show 95% confidence intervals. Asterisks indicate significance levels: *p < 0.05, **p < 0.01 and ***p < 0.001. Marginal significance at *p* < 0.10 is indicated by a dot.

*DHARMa* diagnostics detected no major issues in any linear model fitted to transferability metrics (Figures S1.13–S1.17). Models showed distinct relationships between niche changes and native‐SDM performance in alien backgrounds (Figure 4, Tables S2.11–S.13, Figure S2.20). Niche expansion significantly decreased AUC, TSS, CBI, and Sensitivity (all *p* ≤ 0.01), but showed no significant association with Specificity (Figure 4, Table S2.11, Figure S2.20). Niche unfilling was significantly positively related to Sensitivity (*p* < 0.001), marginally negatively related to Specificity, and significantly negatively related to CBI (*p* = 0.017), with no significant effects on AUC or TSS (Figure 4, Table S2.11, Figure S2.20).

## 4 Discussion

We investigated realized climatic niche changes in established alien mammals and their potential drivers at a global scale. Across species and zoogeographic realms, niche stability was more common than niche expansion, confirming that niche conservatism (Wiens et al. 2010) shapes alien mammal distributions. However, most species only partially filled their native niche in the receiving zoogeographic regions (unfilling), indicating incomplete colonization (Guisan et al. 2014). Niche expansion was generally low, with the highest median in the Australian realm, and occurred more frequently in southern realms, harboring numerous Palearctic‐origin alien species (Biancolini et al. 2021), suggesting a potential influence of zoogeography. By contrast, niche unfilling was high in most realms, particularly in the Panamanian, Saharo‐Arabian, and Afrotropical realms; the Panamanian realm includes many islands, while alien mammals occur only in restricted areas of the Afrotropical and Saharo‐Arabian realms. Finally, native‐range SDMs generally transferred well to receiving regions; however, all performance metrics were affected by niche expansion and unfilling.

### 4.1 Patterns and Drivers of Niche Expansion and Unfilling

Niche unfilling clearly dominated the global ecology of alien mammals, consistent with previous findings in plants (Cao Pinna et al. 2025; Liu, Heberling, et al. 2023; Petitpierre et al. 2012), insects (Hill et al. 2017), freshwater mollusks (Mahapatra et al. 2025), and terrestrial vertebrates (Cardador and Blackburn 2020; Strubbe et al. 2013, 2015). The widespread niche unfilling observed across realms indicates climatic disequilibrium in the alien ranges of mammals, suggesting potential for further spread, as previously highlighted by bioclimatic SDM projections (Biancolini et al. 2024). In contrast, niche expansion was rare and modest across species and realms, aligning with previous findings on the predominant niche stability of alien vertebrates at regional (Strubbe et al. 2013, 2015) and global scales (Broennimann et al. 2021; Cardador and Blackburn 2020; Liu et al. 2020a; Liu et al. 2017).

#### 4.1.1 Species Attributes

We observed a clear negative correlation between niche expansion and native range size, the strongest of the negative drivers. Native range size largely reflects abiotic constraints, that is, the “Grinnellian” niche component in the Grinnellian–Eltonian distinction (Kambach et al. 2019; Slatyer et al. 2013), and species with broad native ranges are therefore less likely to expand into new conditions relative to those experienced natively, consistent with past evidence linking native range size with niche conservatism (Bates et al. 2020; C. Liu et al. 2020a, 2020b; Louppe et al. 2020). For example, *Vulpes vulpes* , whose native range spans much of the Palearctic and Nearctic realms (IUCN 2020), was widely introduced to four realms and showed no niche expansion in any of them. Likewise, insular endemics, which inherently have small native ranges, have shown greater niche expansion after introduction than mainland species (García‐Rodríguez et al. 2023; Stroud 2021). Accordingly, the specialization index, which reflects biotic resource use and thus the “Eltonian” niche component (Sekercioglu 2011; Soberón 2007), showed a negative association with niche expansion, consistent with the idea that exploiting a wider range of habitat and food types may facilitate colonization of new conditions; however, this effect was not supported because the confidence intervals overlapped zero. This aligns with evidence from alien birds, where diet and habitat breadth showed no supported association with niche expansion (Strubbe et al. 2013). Native range loss showed at most a weak association with niche expansion, as the confidence interval overlapped zero. Nevertheless, the positive effect is consistent with the expectation that evaluating niche changes on current native ranges can be problematic when human pressures caused their contraction: if the native climatic niche is truncated (Breiner et al. 2017; Chevalier et al. 2021; Faurby and Araújo 2018; Sales et al. 2022), apparent alien niche expansion may partly reflect “re‐filling” of conditions that were previously part of the realized native niche. At the same time, native range loss showed an opposite and supported association with niche unfilling. This was unexpected considering niche truncation, as a smaller native niche should lead to reduced niche unfilling; however, the positive correlation may instead reflect the vulnerability of species that experienced native‐range contraction (Sales et al. 2022; Tedeschi, Lenzner, Schertler, Biancolini, et al. 2025). The same human pressures that caused it could also act in their alien range, likely limiting species spread and promoting niche unfilling. For example, many of these species declined due to over‐hunting and were introduced as game species (Biancolini et al. 2021; Tedeschi, Lenzner, Schertler, Biancolini et al. 2025). Threatened mammals with alien populations represent a conservation paradox (Tedeschi, Lenzner, Schertler, Biancolini, et al. 2025), and quantifying the extent to which they re‐fill their lost native niches after introduction could help evaluate the conservation value of those populations. Finally, while life‐history strategies and dispersal ability were linked to changes in the climatic niche of native mammals (Di Marco et al. 2021), they appear to have less influence on the climatic niche of alien mammals, since these factors were not included in our final models.

#### 4.1.2 Invasion History

Introduction effort emerged as a main driver of both niche expansion and unfilling, which was expected considering that human‐mediated dispersal moves alien mammals across geographic space (Biancolini and Rondinini 2025) and, consequently, across environmental space in ways that fundamentally differ from native colonization (Wilson et al. 2016). High introduction effort should promote niche expansion by increasing the chances that alien populations encounter and establish in unoccupied climatic conditions (Strubbe et al. 2013, 2015), potentially aided by higher numbers of released individuals that can increase founding genetic variation and facilitate colonization through plasticity or evolution (Simberloff 2009). Consistent with this expectation, we found a positive association between introduction effort and niche expansion, and our analysis identified it as the primary driver among those considered. The absence of this association in previous work (Strubbe et al. 2013, 2015) may reflect the difficulty of reconstructing human‐assisted dispersal from fragmentary introduction records (Buba et al. 2024; Tedeschi, Lenzner, Schertler, Wessely, et al. 2025) together with the rarity of niche expansions across taxa (Li et al. 2014; Liu et al. 2022; Petitpierre et al. 2012; Strubbe et al. 2015). At the same time, greater introduction effort should reduce niche unfilling because it helps overcome environmental and demographic barriers (Kanarek and Webb 2010; Lockwood et al. 2009), and spreads species across multiple introduction locations where climates match the native niche (Biancolini and Rondinini 2025; Strubbe et al. 2013, 2015). In line with this, we found a strong negative association between introduction effort and niche unfilling, consistent with prior evidence in vertebrates at regional and global scales (Li et al. 2014; Pili et al. 2020; Strubbe et al. 2013, 2015). Similarly, longer residence time was associated with lower niche unfilling, consistent with the idea that suitable conditions in the alien range are gradually filled over time (Biancolini and Rondinini 2025; Liu et al. 2024), as populations spread within the receiving regions (Williamson et al. 2009). This result aligns with past research on alien species at regional (Cano‐Barbacil et al. 2023; Strubbe et al. 2013, 2015) and global scales (Liu et al. 2020a, 2020b), suggesting that native niche unfilling decreases as residence time lengthens. This is also in line with known invasion time lags, during which alien populations undergo demographic build‐up before expanding (Daly et al. 2023). Residence time showed a negative effect on niche expansion, against the expectation that long‐established alien populations have had more opportunities to occupy new climates. This may reflect a shift in mammal introduction pathways (Biancolini et al. 2021): older, planned releases (e.g., fauna enhancement, hunting) likely favored sites perceived as climatically matching the native niche (Broennimann et al. 2021), whereas more recent unplanned releases associated with the pet trade may occur into a wider range of climatic conditions. However, the confidence interval of residence time overlapped zero, indicating high uncertainty in the found relationship. A similarly weak negative signal has been reported for invasive endotherms globally (C. Liu et al. 2020a) and, more generally, past research suggests that residence time is not a consistent cross‐taxon driver of niche expansion (Liu et al. 2020a). By contrast, alien insularity showed opposite associations with niche expansion and unfilling: its effect on expansion was negative, but the confidence interval overlapped zero, whereas its effect on unfilling was positive, with a confidence interval that marginally overlapped zero. When terrestrial alien mammals are confined to islands, the limited climatic heterogeneity of these landmasses (Alexander and Edwards 2010; Stroud 2021) may leave much of the native climatic niche locally unavailable. In addition, surrounding seas constrain dispersal, making the new conditions of the receiving regions unreachable without further human assistance (Biancolini and Rondinini 2025; Wilson et al. 2016).

#### 4.1.3 Environmental Conditions

We found a coherent human‐disturbance signal across both niche changes: alien mammals showed higher niche unfilling but lower niche expansion in more disturbed contexts. This fits the idea that invasions often begin in anthropized environments (González‐Moreno et al. 2015; Hill et al. 2017), where populations can persist while occupying only a subset of the climates available in the receiving region, inflating unfilling until spread into less disturbed environments occurs (Dietz and Edwards 2006; Petitpierre et al. 2012). This is consistent with evidence that alien mammals often spread into relatively low‐disturbance ecosystems over time (Liu, Semenchuk, et al. 2023). In parallel, if populations remain concentrated in anthropized landscapes, access to a broader spectrum of conditions may be limited, lowering the likelihood of niche expansion, a pattern reported for alien plants and insects (González‐Moreno et al. 2015; Hill et al. 2017). In our results, *Sciurus stramineus* exemplifies this mechanism, showing high niche unfilling and no expansion in the Panamanian realm while remaining confined to urban areas (Merrick et al. 2012), whereas *Oryctolagus cuniculus* showed high niche expansion in the Australian realm, where it spread across both natural and anthropized environments (McLeod and Saunders 2014). Contrary to expectations, we found a negative effect of native‐mammal richness on niche unfilling, but its confidence interval overlapped zero. This could tentatively be read as weak evidence that diverse ecosystems might still offer available niche space, consistent with alien mammals tending to favor low‐disturbance ecosystems (Liu, Semenchuk, et al. 2023) and with global findings linking native richness to vertebrate range expansion via native‐niche filling (Biancolini and Rondinini 2025; Du et al. 2024; Liu et al. 2014). As expected, community similarity constrained niche expansion, consistent with biotic resistance: introductions into zoogeographically similar areas (Holt et al. 2013) likely exposed alien mammals to more native‐like, co‐evolved competitors and predators, strengthening biotic constraints and limiting the realized alien niche (Daly et al. 2023). Conversely, species introduced to less similar communities may more often benefit from enemy release and weaker biotic constraints, facilitating expansion into climatic conditions not occupied in the native range (Cao Pinna et al. 2025; García‐Rodríguez et al. 2023), as exemplified by *O. cuniculus* in the Australian realm, where native enemies were largely absent (McLeod and Saunders 2014). However, the large random‐effect variance for realms and orders suggests that unmeasured realm‐level and lineage‐specific features may influence niche unfilling beyond what is captured by the considered drivers.

### 4.2 Transferability of Native‐range Species Distribution Models

Consistent with expectations, native‐range SDMs transferred mostly well to receiving zoogeographic regions; however, niche changes generally appeared to reduce their transferability. Increased niche unfilling lowered AUC and Specificity because pseudo‐absences fell into climatically suitable yet currently unoccupied cells, inflating omission errors, and reduced CBI because high‐suitability classes remain unoccupied under incomplete colonization (Adelino and Lima 2023; Strubbe et al. 2015). Because early invasions may occupy sub‐optimal conditions due to dispersal limitations (Daly et al. 2023; Wilson et al. 2009), reduced performance under high niche unfilling should not be interpreted as poor transferability (Liu et al. 2022). Consistently, TSS and Sensitivity increased with niche unfilling, indicating good classification of alien presences despite incomplete colonization, with median Sensitivity > 98%. For management, high Sensitivity is preferable to high Specificity to avoid overlooking invasion‐risk areas (Cordier et al. 2020; Heidy Kikillus et al. 2010).

As we hypothesized, all metrics were negatively correlated with niche expansion, consistent with evidence that native‐range SDMs remain broadly reliable but are impaired by expansion across taxa (C. Liu et al. 2022), including vertebrates (Strubbe et al. 2013, 2015), insects (Hill et al. 2017), and plants (Petitpierre et al. 2012). When niche expansion occurs, alien presences fall in conditions that would be considered unsuitable according to the native niche. Consequently, AUC, TSS, and Sensitivity consider these presences to be misclassified. Similarly, niche expansion increased the presence frequency in low‐suitability classes, lowering CBI. Because niche expansion was rare in alien mammals and overall transferability was good, SDMs remain a useful tool for alien mammal management (Bertolino et al. 2020; Biancolini et al. 2024), consistent with the strong broad‐scale climatic constraint on endotherm distributions (C. Liu et al. 2020b).

### 4.3 Data Constraints and Limitations

Our assessment of alien mammal niche changes was based on observational data that represent the realized niche, which is constrained by geographic barriers, biotic interactions, and human factors (Chevalier et al. 2021; Faurby and Araújo 2018; Guisan et al. 2014). Consequently, niche metrics and SDMs may underestimate climatic tolerances in both native and alien ranges (Arlé et al. 2024). Incorporating biotic interactions remains a long‐recognized (Wisz et al. 2013) and still outstanding challenge in niche modelling, given data constraints (Yates et al. 2018). Thus, our data and methods cannot distinguish whether observed niche expansions reflect access to the fundamental niche after relaxation of dispersal and biotic constraints or evolutionary adaptation (Chevalier et al. 2024; Guisan et al. 2014). Nevertheless, we used zoogeographic regions and native‐mammal richness as a proxy for biotic context (Biancolini and Rondinini 2025). Furthermore, most alien ranges derive from introduction events within the last 150 years (Biancolini et al. 2021), a period likely insufficient for relevant evolutionary changes (Liu, Heberling, et al. 2023). Expert‐drawn range maps depict areas where species are considered “extant” (Biancolini et al. 2021) without occupancy details (Di Marco and Santini 2015; Herkt et al. 2017) and are affected by uneven research effort across taxa and regions (Herkt et al. 2017; Seebens et al. 2025). Nonetheless, by synthesizing multiple data sources, they remain the most comprehensive representation of alien mammal distributions for global and regional analyses (Biancolini and Rondinini 2025; Seebens et al. 2025; Canelles et al. 2025; Tedeschi, Lenzner, Schertler, Wessely, et al. 2025). Introduction records are also biased and typically lack propagule size and frequency for most alien mammals (Biancolini and Rondinini 2025; Long 2003; Tedeschi, Lenzner, Schertler, Wessely, et al. 2025); to address this, we used a proxy for propagule pressure (Biancolini and Rondinini 2025) that combines counts of introduction locations (Capellini et al. 2015; Duncan et al. 2001), compiled through a systematic survey of the literature (Biancolini et al. 2021; Blackburn et al. 2017; Long 2003), with introduction pathways (Biancolini et al. 2021). Some variables showed confidence intervals overlapping zero in the averaged models, likely reflecting limitations of a broad, coarse‐resolution design; additional data, finer metrics, or taxon‐specific analyses may clarify their influence. In addition, although native‐range SDMs generally transferred well, performance metrics were sensitive to both niche expansion and unfilling, making it challenging to discern whether low performance stems from model limitations or incomplete colonization. Despite these constraints, the congruence of our results with previous evidence across taxa and regions supports their robustness. Finally, our conclusions are limited to established alien mammals and exclude failed introductions; however, Broennimann et al. (2021) showed that successful introductions typically occur in climates close to the native niche.

### 4.4 Management and Modeling Recommendations

Alien mammal management should focus on preventing future introductions and prioritizing species whose native niche remains unfilled, which may generate invasion debt in receiving regions (Rouget et al. 2016) or that show niche expansion, which are harder to anticipate from native ecological requirements (Liu et al. 2022). Tightening regulations can curtail opportunities to colonize suitable conditions for alien species, as shown by declining invasion rates in European countries following policy action (Canelles et al. 2025). SDMs can support early detection of alien species by identifying invasion‐risk areas, but they should rely on multiple data sources, including historical records, to avoid niche truncation (Chevalier et al. 2021; Faurby and Araújo 2018; Sales et al. 2022). SDM transferability should be evaluated with multiple performance metrics (Sillero et al. 2021) and interpreted in light of niche expansion and unfilling. Continuous monitoring can reveal whether currently unoccupied suitable climates are colonized over time (Johnson et al. 2023), and new records can help distinguish colonization lags from SDM misclassification.

### 4.5 Future Directions

Future research should address the bias introduced by anthropogenic truncation of native ranges when quantifying niche expansion. Using before‐impact native distributions, where historical and paleo‐ecological evidence allows, would help separate genuine niche expansion from re‐filling of the lost native niche, and would contribute to the conservation paradox discussion by clarifying whether alien populations of threatened mammals occupy conditions that were previously occupied in the native range and potentially relevant as ex‐situ refugia. Furthermore, modeling potential interactions between newcomers and receiving communities would help distinguish between climatically suitable areas that remain unoccupied due to competition or predation and areas where enemy release promotes spread. A valuable next step would be to test niche dynamics at finer ecological and spatial scales, where local and regional variability may reveal patterns and drivers obscured at broader scales. Ultimately, advancing this area of research will require integrating observation‐based analyses with independent evidence on the theoretical niche, such as physiological limits, to anticipate spread into novel conditions.

## Author Contributions

**Dino Biancolini:** conceptualization, data curation, formal analysis, funding acquisition, investigation, methodology, software, validation, visualization, writing – original draft, writing – review and editing. **Olivier Broennimann:** conceptualization, methodology, software, validation, visualization, writing – review and editing. **Antoine Guisan:** conceptualization, methodology, resources, supervision, writing – review and editing. **Carlo Rondinini:** conceptualization, funding acquisition, methodology, project administration, resources, supervision, writing – review and editing.

## Funding

This work was supported by Sapienza Università di Roma.

## Conflicts of Interest

The authors declare no conflicts of interest.

## Supporting information


**Appendix S1:** Supporting Methods.

**Appendix S2:** Supporting Results.

## Data Availability

Data and code supporting our findings, including species presences and R scripts for niche analysis, GLMMs, and native‐range SDM transferability, are available in a dedicated Figshare repository (https://doi.org/10.6084/m9.figshare.29546054), while codes for variable processing and building native‐range SDMs are available from Biancolini and Rondinini (2025) (https://doi.org/10.6084/m9.figshare.28100279). We used zoogeographic realms and mammal regions from http://macroecology.ku.dk/resources/wallace; mammals' alien ranges, residence time, and alien insularity from DAMA (https://doi.org/10.1002/ecy.3474); life‐history traits and island endemicity from COMBINE (https://doi.org/10.1002/ecy.3344); native ranges and habitat breadth from the International Union for the Conservation of Nature (https://www.iucnredlist.org/resources/spatial‐data‐download); diet breadth from MammalDIET (https://doi.org/10.1002/ece3.1136); present natural ranges from Phylacine 1.2 (https://doi.org/10.1002/ecy.2443); bioclimatic variables from CHELSA 2.1 (https://doi.org/10.1038/sdata.2017.122); human footprint from https://doi.org/10.1038/s41597‐022‐01284‐8; native‐mammal richness from https://datadryad.org/dataset/doi:10.5061/dryad.02v6wwq48; and introduction records from DAMA and https://doi.org/10.3897/neobiota.33.10471.
