# Supplementary material for: Global Patterns of Niche Changes in Alien Mammals: Potential Drivers and Significance for Invasion Projections
Source: Glob Chang Biol. 2026 Mar 20;32(3):e70755. doi: 10.1111/gcb.70755 (PMC13004023; doi:10.1111/gcb.70755)
Supplement: Supplementary file 1 — Appendix S1: Supporting Methods. [file GCB-32-e70755-s002.pdf]

Global Change Biology

Appendix S1 for:

Global patterns of niche changes in alien mammals: potential drivers and significance for invasion projections

Table of Contents

1. Supporting Methods	Page 1
1.1 Species presences and pseudo-absences	Page 1
Figure S1.1: Boxplots of a) global native and alien presences and b) realm-level alien presences, both on a logarithmic scale.	Page 2
Table S1.1: Number of sampled presences (occupied cells) of each species within its native, alien and total ranges and the total number of zoo-regions (Holt et al. 2013) in which they are found.	Page 3
Table S1.2: Number of sampled presences (occupied cells) of each species within its realm-level alien range and occupied zoo-regions per realm.	Page 7
Table S1.3. Cases excluded from niche analyses due to insufficient number of presences (< 5) in the native and/or the alien range.	Page 14
1.2 Analysis of niche changes	Page 15
1.2.1 Novel climates assessment	Page 15
Figure S1.2: The proportion of cells in each species' alien background showing Type 1 (left) and Type 2 (right) climate novelty across zoogeographic realms, based on the Extrapolation Detection analysis. The dashed line indicates a 5% threshold.	Page 16
1.2.2 Phylogenetic signal	Page 16
Table S1.4: The proportion of cells in each species' alien background showing Type 1 and Type 2 climate novelty across zoogeographic realms, based on the Extrapolation Detection analysis	Page 17
1.3 Variables statistics	Page 24
Table S1.5. Variance Inflation Factor values for raw numerical variables.	Page 24
Figure S1.3: Scaled values of the Fast-growth index across orders.	Page 25
Figure S1.4: Scaled values of Dispersal across orders.	Page 25
Figure S1.5: Scaled values of Native ranges sizes across orders.	Page 25
Figure S1.6: Scaled values of the Specialization index across orders.	Page 25
Figure S1.7: Scaled values of Native range loss across orders.	Page 26
Figure S1.8: Scaled values of Introduction effort across realms.	Page 26
Figure S1.9: Scaled values of Residence time across realms.	Page 26

Figure S1.10: Alien insularity across realms. Alien ranges confined to islands (represented as 1) are depicted in green, while those extending to both islands and the mainland (represented as 0) are shown in blue.	Page 26
Figure S1.11: Scaled values of Human disturbance across realms.	Page 27
Figure S1.12: Scaled values of Native mammal richness across realms.	Page 27
Figure S1.13: Scaled values of Community similarity across realms.	Page 27
1.4 Transformations of niche changes and their drivers	Page 28
Figure S1.15: Goodness-of-fit diagnostics for the beta distribution fitted to the niche unfilling values. The plots show: (top-left) empirical histogram and theoretical beta density (red line), (top-right) Q-Q plot comparing empirical and theoretical quantiles, (bottom-left) empirical and theoretical cumulative distribution functions and (bottom-right) P-P plot comparing empirical and theoretical probabilities.	Page 28
1.4.1 Fixed-response variable relationships	Page 29
Figure S1.17: Response plots showing the relationship between niche expansion (y-axis) and seven fixed variables (x-axes) presented in both their raw and transformed version. The black line represents the modeled relationship based on the transformed variables, and the gray shaded area is the 95% confidence interval.	Page 30
Figure S1.18: Response plots showing the relationship between niche unfilling (y-axis) and eight fixed variables (x-axes) presented in both their raw and transformed version. The black line represents the modeled relationship based on the transformed variables, and the gray shaded area is the 95% confidence interval.	Page 31
1.5 Transferability of native species distribution models	Page 32
1.5.1 Transformation of transferability metrics	Page 33
Figure S1.16: Goodness-of-fit diagnostics for the beta distribution fitted to the transformed AUC, TSS and CBI values. The plots show: (first-row) empirical histogram and theoretical beta density (red line), (second-row) Q-Q plot comparing empirical and theoretical quantiles, (third-row) empirical and theoretical cumulative distribution functions and (forth-row) P-P plot comparing empirical and theoretical probabilities.	Page 33
1.6 GLMM diagnostics	Page 34
1.7 Graphic visualization	Page 35
1.8 References	Page 36

1. Supporting Methods

Here we summarize the key steps of our methodological workflow; see the data and scripts on Figshare (https://figshare.com/s/50927b9d2c1351ad6d82) for further details. All spatial operations were performed using the WGS84 projection (EPSG:4326).

1.1 Species presences and pseudo-absences

We obtained species alien and native ranges from DAMA: the global Distribution of Alien Mammals database (Biancolini et al., 2021), the IUCN Red List (IUCN, 2020). To define the alien and native backgrounds for each species, we utilized the mammal zoogeographic regions (zoo-regions) identified by Holt in 2013. Specifically, we used the "Join Attributes by Location" function in QGIS 3.34 (https://qgis.org) to overlay the native and alien ranges onto the zoo-regions, both of which were in shapefile format. This process allowed us to retain only zoo-regions that contained a portion of the native or alien range (Table S1.1 and S1.2) (Biancolini et al., 2024). The median number of zoo-regions was 3 (Table S1.1) per native background and 1 per alien background (Table S1.2).

We generated species presences and pseudo-absences by rasterizing the range maps and the backgrounds, both in polygon format, at a resolution of 5×5 arc-minutes (~10 km at the equator). We created a loop in R to rasterize and extract the native and alien presence/pseudo-absences of each species, along with realm information for alien ranges. During each iteration, we rasterized the background and range shapefiles for the current species using the "rasterize" function, assigning a value of "0" to the pseudo-absences cells and "1" to the range cells. Subsequently, we converted the resulting rasters into point data utilizing the "as.points" function from the *terra* 1.7-71 R package (Hijmans, Bivand, et al., 2024) and the "st_as_sf" function from the Simple Features 1.0-15 package (Pebesma et al., 2023). We then extracted the longitude and latitude coordinates of each cell using the "st_coordinates" function from Simple Features. Finally, we combined the presence and pseudo-absence data into a single data frame, ensuring that any pseudo-absence coordinates that overlapped with presence coordinates were removed, using the "rbind" and "duplicated" functions. We saved the occurrence data as CSV files. Both native and alien tables contained "Longitude", "Latitude", and "Value" columns. However, the alien tables also included a "Realm" column. The native distribution of *Camelus dromedarius* was originally a raster (Faurby et al., 2018), making the rasterization steps unnecessary. Presence or pseudo-

absences lacking climatic information were removed (Table S1.1 and S1.2). For a species to be analyzed within a realm, it must have at least five presences in its native range and at least five presences in that corresponding realm (Di Cola et al., 2017). Any species-realm combination that failed to meet this dual threshold was excluded from that specific analysis. Of the 46 excluded cases, the vast majority (42) were due to having sufficient native presences but fewer than five presences in the alien range. The remaining exclusions consisted of three cases that lacked sufficient presences in both ranges and one case that lacked presences in the native range despite having enough in the alien range (Table S1.3). Presence tables are available in CSV format (Native_presences.csv and Alien_presences.csv) on Figshare.

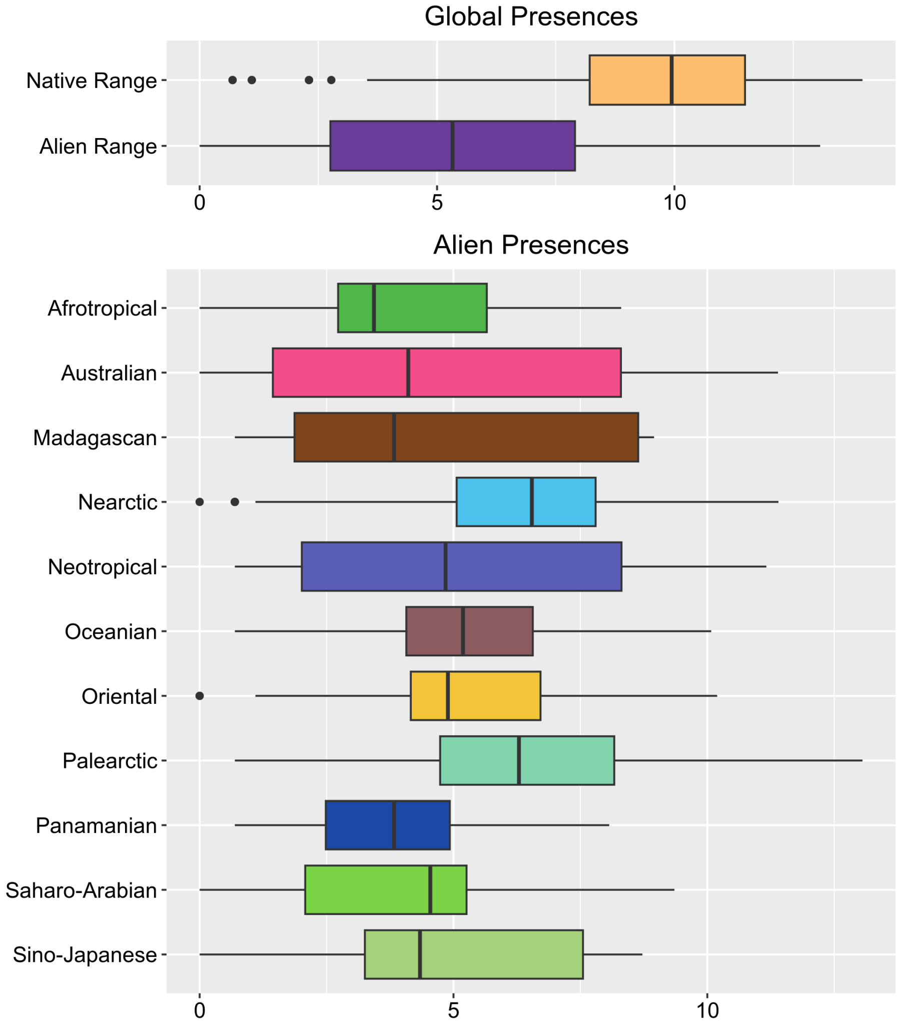

Figure S1.1: Boxplots of a) global native and alien presences and b) realm-level alien presences, both on a logarithmic scale.

Table S1.1: Number of sampled presences (occupied cells) of each species within its native, alien and total ranges and the total number of zoo-regions (Holt et al. 2013) in which they are found.

	**Occurrences**		**Zoo-regions**	
**Binomial**	**Native**	**Alien**	**Alien**	**Native**
Alces alces	527927	2479	1	6
Ammotragus lervia	20092	7590	4	2
Antilope cervicapra	22661	7240	5	1
Apodemus sylvaticus	82304	10490	4	2
Atelerix algirus	10050	1361	3	2
Atlantoxerus getulus	4384	42	2	2
Axis axis	30313	19529	10	3
Axis porcinus	4263	653	3	5
Babyrousa babyrussa	101	132	1	1
Bandicota indica	68880	2382	2	4
Beatragus hunteri	158	25	1	1
Bettongia lesueur	42	5	1	2
Bettongia penicillata	114	8	2	3
Bison bison	3943	214	2	1
Bos javanicus	2818	3267	3	3
Boselaphus tragocamelus	29611	650	3	2
Bubalus bubalis	302	27700	10	3
Callithrix geoffroyi	1570	102	1	2
Callithrix jacchus	11490	14384	2	1
Callithrix penicillata	16391	7230	2	2
Callosciurus erythraeus	53727	979	4	4
Callosciurus finlaysonii	9366	30	3	1
Callosciurus notatus	18979	19	1	2
Callosciurus prevostii	16339	326	1	2
Camelus dromedarius	296559	44924	3	10
Capra aegagrus	21465	381	1	6
Capra ibex	777	55	1	1
Capra sibirica	27743	148	1	6
Capreolus capreolus	126761	22	1	3
Capreolus pygargus	220047	3383	2	7
Castor canadensis	287755	12915	4	4
Castor fiber	80635	438	1	2
Cephalophus adersi	99	2	1	2
Cercopithecus mona	7544	35	2	2
Cervus canadensis	106830	276	3	8
Cervus elaphus	80914	21335	8	5
Cervus nippon	6164	18939	6	6
Chaetophractus villosus	37809	47	1	2
Chlorocebus sabaeus	18612	53	3	3
Civettictis civetta	183499	37	1	5
Connochaetes taurinus	39634	589	1	2
Cricetomys gambianus	50060	2	1	4
Crocidura dsinezumi	4811	171	1	3
Crocidura maxi	3212	61	2	2
Crocidura monticola	17602	70	1	2
Crocidura pachyura	657	462	2	2
Crocidura russula	31858	337	2	3
Crocidura suaveolens	212197	384	1	9
Cuniculus paca	161799	200	1	5
Dama dama	1864	67044	13	1
Dasyprocta leporina	55332	177	3	2

Dasyprocta mexicana	956	193	1	2
Dasyprocta punctata	16614	209	1	2
Dasypus novemcinctus	226650	1411	3	9
Dasyurus hallucatus	4806	3	1	3
Daubentonia madagascariensis	1951	3	1	1
Dendrolagus matschiei	188	44	1	1
Desmana moschata	44823	3941	3	3
Didelphis aurita	18826	2	1	2
Didelphis marsupialis	112516	210	2	6
Didelphis virginiana	79980	7115	2	5
Elephas maximus	10101	277	1	4
Eliomys quercinus	43735	744	1	2
Erinaceus europaeus	76931	7696	3	2
Erinaceus roumanicus	115791	202	1	3
Erythrocebus patas	86303	5	1	4
Eulemur albifrons	292	2	1	1
Eulemur fulvus	4864	14	1	1
Eulemur mongoz	141	46	1	1
Eutamias sibiricus	315053	57	3	6
Funambulus pennantii	38192	131	4	5
Gazella subgutturosa	110399	6	1	9
Genetta genetta	174574	18495	1	6
Geocapromys ingrahami	2	2	1	1
Glis glis	66736	168	1	4
Hemitragus jemlahicus	1974	496	2	3
Herpestes auropunctatus	55899	4789	12	7
Herpestes edwardsii	56842	2	1	7
Herpestes fuscus	712	167	1	1
Hippopotamus amphibius	19262	175	1	4
Hydrochoerus hydrochaeris	163652	2	1	4
Hydropotes inermis	2857	929	1	3
Hystrix cristata	64212	3667	1	5
Hystrix javanica	2182	233	1	2
Isoodon obesulus	3658	7	1	2
Lagorchestes hirsutus	10	2	1	1
Lagostrophus fasciatus	10	3	1	1
Lama glama	25880	2	1	3
Lama guanicoe	25880	2	1	3
Lasiorhinus latifrons	1174	3	1	2
Leggadina lakedownensis	17070	2	1	3
Leporillus conditor	2	6	1	1
Lepus americanus	199955	2892	2	3
Lepus arcticus	113271	13	1	1
Lepus californicus	64441	7	1	4
Lepus capensis	149744	445	1	12
Lepus corsicanus	2076	93	1	1
Lepus europaeus	194050	137662	11	4
Lepus granatensis	8351	242	1	1
Lepus nigricollis	42925	264	2	5
Lepus timidus	490389	527	3	5
Lycalopex griseus	25096	1169	1	3
Macaca arctoides	18964	7	1	3
Macaca cyclopis	366	64	2	1
Macaca fascicularis	35113	850	5	2
Macaca leonina	20027	15	1	4

Macaca mulatta	99601	100	4	7
Macaca nemestrina	17061	69	1	2
Macaca nigra	104	41	1	1
Macaca sylvanus	744	1	1	3
Macropus agilis	17491	99	2	4
Macropus eugenii	371	79	2	2
Macropus fuliginosus	23076	2	1	3
Macropus giganteus	30512	12	1	3
Macropus parma	816	4	1	1
Macropus rufogriseus	9626	167	2	2
Manis culionensis	273	12	1	1
Marmota bobak	15077	718	1	3
Marmota marmota	3343	539	1	1
Martes americana	166248	2293	2	2
Martes foina	202551	308	2	12
Martes martes	203238	589	1	4
Martes melampus	4429	321	2	2
Martes zibellina	237353	5489	2	3
Meles meles	183970	179	1	6
Micromys minutus	336222	1765	2	9
Microtus arvalis	132016	52	1	4
Microtus californicus	4625	20	1	2
Microtus levis	71718	199	2	4
Muntiacus muntjak	18844	114	1	2
Muntiacus reevesi	30193	3273	2	3
Mus caroli	22814	64	2	2
Mus spretus	14997	124	1	3
Mus terricolor	37250	129	1	4
Mustela erminea	969394	4725	2	12
Mustela itatsi	4687	1579	2	2
Mustela lutreola	11619	148	1	2
Mustela nivalis	926855	17620	5	12
Mustela putorius	116705	6201	3	3
Mustela sibirica	239564	2214	1	9
Myocastor coypus	56725	62144	9	4
Myodes gapperi	158516	2287	1	3
Myodes glareolus	184964	1000	1	2
Myodes rutilus	459336	46	1	5
Nanger soemmerringii	3803	31	2	3
Nasua nasua	150711	87	1	5
Neovison vison	285704	386977	10	3
Nyctereutes procyonoides	96092	166587	5	7
Odocoileus hemionus	106488	1933	3	4
Odocoileus virginianus	220598	7028	7	7
Ondatra zibethicus	279493	473757	9	3
Oreamnos americanus	19234	7974	1	1
Ornithorhynchus anatinus	17296	26	1	2
Oryctolagus cuniculus	16036	137869	15	3
Oryx gazella	23100	516	1	1
Ovibos moschatus	218745	34565	3	2
Ovis canadensis	11599	646	3	3
Ovis orientalis	33220	23305	4	8
Paguma larvata	83981	4825	2	7
Paradoxurus hermaphroditus	96131	3630	3	7
Parantechinus apicalis	61	2	1	1

Peromyscus fraterculus	2264	3	1	1
Peromyscus maniculatus	224193	2326	2	4
Petaurus breviceps	36388	1511	2	5
Petrogale lateralis	1735	9	1	2
Petrogale penicillata	1529	38	2	1
Petrogale persephone	77	1	1	1
Petrogale rothschildi	1916	4	1	1
Phalanger orientalis	4546	1930	3	2
Phascolarctos cinereus	15415	501	2	2
Piliocolobus kirkii	34	1	1	2
Potamochoerus larvatus	72555	5466	2	4
Potorous gilbertii	3	2	1	1
Procyon lotor	183158	32876	8	5
Pseudocheirus peregrinus	13510	88	2	2
Pseudomys fieldi	3	4	1	1
Rangifer tarandus	520121	1403	4	4
Rattus argentiventer	21772	6949	3	3
Rattus exulans	39207	24650	7	3
Rattus nitidus	48641	1198	3	6
Rattus praetor	3333	1048	1	1
Rattus tanezumi	98401	33474	4	9
Rupicapra rupicapra	6196	1485	2	1
Rusa marianna	1885	44	1	1
Rusa timorensis	237	16185	6	1
Rusa unicolor	97748	2904	5	6
Saguinus oedipus	708	8	1	2
Saimiri sciureus	53167	12	2	2
Sapajus apella	40572	4	1	3
Sapajus nigritus	11663	2	1	2
Sarcophilus harrisii	1144	7	1	1
Sciurus aberti	4250	2982	2	3
Sciurus anomalus	10143	12	1	2
Sciurus aureogaster	8047	2	1	3
Sciurus carolinensis	64354	8518	3	3
Sciurus granatensis	12488	4	1	2
Sciurus niger	66757	20890	3	4
Sciurus stramineus	2002	6	1	2
Sciurus vulgaris	442329	2794	2	5
Semnopithecus entellus	7148	123	1	2
Sorex cinereus	238428	2287	1	3
Spilocuscus maculatus	10863	521	2	3
Suncus etruscus	49140	7438	3	11
Suncus murinus	109744	13424	11	9
Sundasciurus juvencus	159	1	1	1
Sus celebensis	2592	1606	3	1
Sylvilagus floridanus	100104	3342	4	6
Tachyglossus aculeatus	103103	7	1	4
Tamandua tetradactyla	154928	2	1	4
Tamias striatus	68923	59	1	2
Tamiasciurus hudsonicus	209832	3565	1	3
Tenrec ecaudatus	7659	155	3	1
Thylogale billardierii	1197	8	1	1
Thylogale browni	2852	692	1	1
Thylogale brunii	6628	13	1	1
Trachypithecus auratus	1782	82	1	1

Global Change Biology

Tragelaphus angasii	2956	4038	1	1
Tragelaphus derbianus	38820	18	1	4
Tragulus nigricans	16	3	1	1
Trichosurus vulpecula	37479	4681	1	3
Urocitellus parryii	149080	497	2	2
Varecia variegata	247	2	1	1
Vicugna vicugna	8217	7	1	2
Viverra tangalunga	16330	5014	3	2
Viverra zibetha	66513	93	1	5
Viverricula indica	110711	8177	6	6
Vombatus ursinus	5107	7	1	1
Vulpes lagopus	371984	554	3	3
Vulpes vulpes	1155259	179291	7	17
Wallabia bicolor	17041	4	1	2

Table S1.2: Number of sampled presences (occupied cells) of each species within its realm-level alien range and occupied zoo-regions per realm.

**Species**	**Realm**	**Occurrences**	**Zoo-regions**
Bettongia lesueur	Australian	5	1
Cervus nippon	Oriental	13	1
Eulemur fulvus	Madagascan	14	1
Nanger soemmerringii	Afrotropical	31	1
Petrogale penicillata	Nearctic	34	1
Macaca nigra	Oceanian	41	1
Eulemur mongoz	Madagascan	46	1
Mustela lutreola	Sino-Japanese	52	1
Crocidura monticola	Oceanian	70	1
Lepus nigricollis	Oceanian	91	1
Mustela itatsi	Oriental	122	1
Crocidura dsinezumi	Sino-Japanese	130	1
Ovis orientalis	Nearctic	174	1
Lepus europaeus	Saharo-Arabian	181	1
Ovis orientalis	Saharo-Arabian	181	1
Vulpes vulpes	Saharo-Arabian	181	1
Rangifer tarandus	Oceanian	192	1
Herpestes auropunctatus	Nearctic	294	1
Rattus exulans	Nearctic	315	1
Petaurus breviceps	Oceanian	367	1
Axis axis	Nearctic	3241	4
Myocastor coypus	Nearctic	30420	4
Lepus europaeus	Neotropical	70481	4
Nyctereutes procyonoides	Palearctic	166569	4
Neovison vison	Palearctic	367228	4
Ondatra zibethicus	Palearctic	468073	4
Ovis canadensis	Nearctic	646	3
Suncus murinus	Afrotropical	1185	3
Dama dama	Nearctic	1380	3
Cervus nippon	Nearctic	1764	3
Neovison vison	Sino-Japanese	2080	3
Ondatra zibethicus	Sino-Japanese	2709	3
Desmana moschata	Palearctic	3941	3
Cervus elaphus	Neotropical	4037	3
Dama dama	Neotropical	4090	3
Bubalus bubalis	Australian	5752	3
Ammotragus lervia	Nearctic	6266	3

Sciurus niger	Nearctic	20890	3
Camelus dromedarius	Australian	44924	3
Oryctolagus cuniculus	Australian	88567	3
Vulpes vulpes	Australian	88638	3
Vulpes vulpes	Nearctic	89409	3
Herpestes auropunctatus	Sino-Japanese	7	2
Bettongia penicillata	Australian	8	2
Saimiri sciureus	Neotropical	12	2
Atelerix algirus	Saharo-Arabian	36	2
Atlantoxerus getulus	Saharo-Arabian	42	2
Oryctolagus cuniculus	Saharo-Arabian	48	2
Eutamias sibiricus	Palearctic	49	2
Dama dama	Panamanian	64	2
Macropus eugenii	Australian	79	2
Rusa unicolor	Nearctic	86	2
Pseudocheirus peregrinus	Australian	88	2
Macaca mulatta	Sino-Japanese	92	2
Rattus exulans	Oriental	101	2
Dasyprocta leporina	Panamanian	123	2
Viverricula indica	Afrotropical	139	2
Axis porcinus	Oriental	158	2
Procyon lotor	Panamanian	163	2
Rattus nitidus	Oriental	189	2
Cervus elaphus	Nearctic	192	2
Bison bison	Nearctic	214	2
Callosciurus erythraeus	Sino-Japanese	291	2
Vulpes lagopus	Palearctic	401	2
Rattus exulans	Australian	466	2
Procyon lotor	Nearctic	469	2
Phalanger orientalis	Oriental	480	2
Phascolarctos cinereus	Australian	501	2
Boselaphus tragocamelus	Nearctic	625	2
Herpestes auropunctatus	Neotropical	661	2
Antilope cervicapra	Nearctic	748	2
Macaca fascicularis	Oriental	776	2
Rangifer tarandus	Palearctic	866	2
Bubalus bubalis	Oriental	875	2
Suncus murinus	Saharo-Arabian	921	2
Sus celebensis	Oriental	1063	2
Micromys minutus	Palearctic	1765	2
Odocoileus virginianus	Panamanian	1798	2
Odocoileus hemionus	Nearctic	1933	2
Sylvilagus floridanus	Nearctic	2145	2
Martes americana	Nearctic	2293	2
Peromyscus maniculatus	Nearctic	2326	2
Rusa unicolor	Australian	2811	2
Lepus americanus	Nearctic	2892	2
Paradoxurus hermaphroditus	Oriental	2935	2
Sciurus aberti	Nearctic	2982	2
Herpestes auropunctatus	Panamanian	3190	2
Bos javanicus	Oriental	3214	2
Capreolus pygargus	Palearctic	3383	2
Suncus murinus	Oriental	3432	2
Rusa timorensis	Oriental	3839	2
Viverra tangalunga	Oriental	4266	2

Apodemus sylvaticus	Saharo-Arabian	4417	2
Rusa timorensis	Australian	4525	2
Odocoileus virginianus	Palearctic	4794	2
Oryctolagus cuniculus	Neotropical	5357	2
Martes zibellina	Palearctic	5489	2
Antilope cervicapra	Neotropical	6474	2
Rattus argentiventer	Oriental	6900	2
Didelphis virginiana	Nearctic	7115	2
Axis axis	Australian	7116	2
Callithrix penicillata	Neotropical	7230	2
Axis axis	Neotropical	9016	2
Lepus europaeus	Nearctic	10567	2
Bubalus bubalis	Neotropical	10570	2
Castor canadensis	Palearctic	10756	2
Mustela nivalis	Saharo-Arabian	11526	2
Dama dama	Australian	13794	2
Callithrix jacchus	Neotropical	14384	2
Neovison vison	Neotropical	15378	2
Cervus nippon	Palearctic	16951	2
Cervus elaphus	Australian	17098	2
Lepus europaeus	Australian	22776	2
Ovis orientalis	Palearctic	22904	2
Procyon lotor	Palearctic	26112	2
Rattus tanezumi	Oriental	26745	2
Ovibos moschatus	Palearctic	27129	2
Myocastor coypus	Palearctic	27156	2
Lepus europaeus	Palearctic	33645	2
Oryctolagus cuniculus	Palearctic	43414	2
Dama dama	Palearctic	45096	2
Callosciurus finlaysonii	Oriental	1	1
Chlorocebus sabaeus	Nearctic	1	1
Piliocolobus kirkii	Afrotropical	1	1
Sciurus vulgaris	Sino-Japanese	1	1
Macaca sylvanus	Saharo-Arabian	1	1
Petrogale persephone	Australian	1	1
Sundasciurus juvencus	Oriental	1	1
Cricetomys gambianus	Nearctic	2	1
Funambulus pennantii	Panamanian	2	1
Lama glama	Palearctic	2	1
Macaca mulatta	Nearctic	2	1
Potorous gilbertii	Australian	2	1
Sciurus aureogaster	Nearctic	2	1
Cephalophus adersi	Afrotropical	2	1
Dama dama	Oceanian	2	1
Didelphis aurita	Neotropical	2	1
Eulemur albifrons	Madagascan	2	1
Gazella subgutturosa	Palearctic	2	1
Geocapromys ingrahami	Panamanian	2	1
Herpestes edwardsii	Saharo-Arabian	2	1
Hydrochoerus hydrochaeris	Neotropical	2	1
Lagorchestes hirsutus	Australian	2	1
Lama guanicoe	Neotropical	2	1
Leggadina lakedownensis	Australian	2	1
Macropus fuliginosus	Australian	2	1
Parantechinus apicalis	Australian	2	1

Sapajus nigritus	Neotropical	2	1
Tamandua tetradactyla	Neotropical	2	1
Varecia variegata	Madagascan	2	1
Daubentonia madagascariensis	Madagascan	3	1
Funambulus pennantii	Australian	3	1
Lasiorhinus latifrons	Australian	3	1
Peromyscus fraterculus	Nearctic	3	1
Tragulus nigricans	Oriental	3	1
Dasyurus hallucatus	Oceanian	3	1
Lagostrophus fasciatus	Australian	3	1
Macropus parma	Australian	4	1
Petrogale penicillata	Australian	4	1
Sapajus apella	Neotropical	4	1
Sciurus granatensis	Panamanian	4	1
Wallabia bicolor	Australian	4	1
Gazella subgutturosa	Saharo-Arabian	4	1
Nasua nasua	Neotropical	4	1
Petrogale rothschildi	Australian	4	1
Pseudomys fieldi	Australian	4	1
Cervus canadensis	Palearctic	5	1
Crocidura pachyura	Saharo-Arabian	5	1
Erythrocebus patas	Panamanian	5	1
Callosciurus finlaysonii	Palearctic	6	1
Leporillus conditor	Australian	6	1
Macaca mulatta	Panamanian	6	1
Sciurus stramineus	Neotropical	6	1
Hemitragus jemlahicus	Afrotropical	7	1
Isoodon obesulus	Australian	7	1
Lepus californicus	Nearctic	7	1
Macaca arctoides	Panamanian	7	1
Rusa unicolor	Afrotropical	7	1
Sarcophilus harrisii	Australian	7	1
Tachyglossus aculeatus	Australian	7	1
Vicugna vicugna	Neotropical	7	1
Vombatus ursinus	Australian	7	1
Bubalus bubalis	Saharo-Arabian	8	1
Cervus elaphus	Palearctic	8	1
Eutamias sibiricus	Sino-Japanese	8	1
Saguinus oedipus	Neotropical	8	1
Thylogale billardierii	Australian	8	1
Petrogale lateralis	Australian	9	1
Cercopithecus mona	Neotropical	10	1
Lepus europaeus	Panamanian	12	1
Macropus giganteus	Australian	12	1
Manis culionensis	Oriental	12	1
Sciurus anomalus	Palearctic	12	1
Herpestes auropunctatus	Afrotropical	13	1
Lepus arcticus	Nearctic	13	1
Thylogale brunii	Oceanian	13	1
Macaca leonina	Oriental	15	1
Macropus agilis	Australian	15	1
Odocoileus virginianus	Neotropical	15	1
Antilope cervicapra	Panamanian	18	1
Mustela nivalis	Afrotropical	18	1
Nyctereutes procyonoides	Sino-Japanese	18	1

Spilocuscus maculatus	Oriental	18	1
Tragelaphus derbianus	Panamanian	18	1
Callosciurus notatus	Oriental	19	1
Microtus californicus	Nearctic	20	1
Herpestes auropunctatus	Madagascan	21	1
Capreolus capreolus	Palearctic	22	1
Macaca fascicularis	Sino-Japanese	22	1
Axis axis	Palearctic	23	1
Callosciurus finlaysonii	Sino-Japanese	23	1
Chlorocebus sabaeus	Panamanian	24	1
Macropus rufogriseus	Palearctic	24	1
Beatragus hunteri	Afrotropical	25	1
Boselaphus tragocamelus	Panamanian	25	1
Cercopithecus mona	Afrotropical	25	1
Ornithorhynchus anatinus	Australian	26	1
Dasypus novemcinctus	Neotropical	27	1
Chlorocebus sabaeus	Afrotropical	28	1
Funambulus pennantii	Oriental	32	1
Dasypus novemcinctus	Panamanian	36	1
Civettictis civetta	Afrotropical	37	1
Muntiacus reevesi	Sino-Japanese	37	1
Crocidura dsinezumi	Palearctic	41	1
Dendrolagus matschiei	Oceanian	44	1
Rusa marianna	Oceanian	44	1
Myodes rutilus	Palearctic	46	1
Ovis orientalis	Panamanian	46	1
Chaetophractus villosus	Neotropical	47	1
Rattus argentiventer	Oceanian	49	1
Martes foina	Nearctic	51	1
Macaca fascicularis	Oceanian	52	1
Microtus arvalis	Palearctic	52	1
Bos javanicus	Australian	53	1
Dasyprocta leporina	Neotropical	54	1
Capra ibex	Palearctic	55	1
Suncus murinus	Sino-Japanese	58	1
Oryctolagus cuniculus	Sino-Japanese	59	1
Tamias striatus	Nearctic	59	1
Tenrec ecaudatus	Madagascan	60	1
Crocidura maxi	Oceanian	61	1
Herpestes auropunctatus	Oriental	64	1
Macaca cyclopis	Sino-Japanese	64	1
Mus caroli	Oriental	64	1
Macaca nemestrina	Oriental	69	1
Cervus canadensis	Australian	70	1
Odocoileus virginianus	Australian	71	1
Sciurus carolinensis	Afrotropical	71	1
Oryctolagus cuniculus	Panamanian	73	1
Bubalus bubalis	Panamanian	78	1
Trachypithecus auratus	Oriental	82	1
Didelphis marsupialis	Panamanian	83	1
Nasua nasua	Palearctic	83	1
Macropus agilis	Oceanian	84	1
Lepus corsicanus	Palearctic	93	1
Viverra zibetha	Oriental	93	1
Funambulus pennantii	Saharo-Arabian	94	1

Oryctolagus cuniculus	Nearctic	94	1
Tenrec ecaudatus	Oceanian	95	1
Mustela lutreola	Palearctic	96	1
Callithrix geoffroyi	Neotropical	102	1
Myocastor coypus	Afrotropical	104	1
Mustela erminea	Palearctic	105	1
Rupicapra rupicapra	Palearctic	105	1
Muntiacus muntjak	Oriental	114	1
Suncus murinus	Oceanian	115	1
Semnopithecus entellus	Oriental	123	1
Mus spretus	Palearctic	124	1
Didelphis marsupialis	Neotropical	127	1
Mus terricolor	Oriental	129	1
Rangifer tarandus	Neotropical	131	1
Babyrousa babyrussa	Oceanian	132	1
Axis axis	Oriental	133	1
Sylvilagus floridanus	Panamanian	138	1
Macropus rufogriseus	Australian	143	1
Callosciurus erythraeus	Palearctic	148	1
Capra sibirica	Palearctic	148	1
Vulpes lagopus	Nearctic	153	1
Herpestes fuscus	Oceanian	167	1
Glis glis	Palearctic	168	1
Lepus nigricollis	Oriental	173	1
Hippopotamus amphibius	Neotropical	175	1
Herpestes auropunctatus	Palearctic	178	1
Meles meles	Palearctic	179	1
Suncus etruscus	Saharo-Arabian	192	1
Dasyprocta mexicana	Panamanian	193	1
Microtus levis	Palearctic	199	1
Cuniculus paca	Panamanian	200	1
Cervus canadensis	Nearctic	201	1
Erinaceus roumanicus	Palearctic	202	1
Dasyprocta punctata	Panamanian	209	1
Cervus nippon	Australian	211	1
Rangifer tarandus	Nearctic	214	1
Hystrix javanica	Oriental	233	1
Lepus granatensis	Palearctic	242	1
Martes foina	Palearctic	257	1
Oryctolagus cuniculus	Oceanian	257	1
Elephas maximus	Oriental	277	1
Martes melampus	Sino-Japanese	321	1
Callosciurus prevostii	Oriental	326	1
Viverricula indica	Oriental	326	1
Crocidura russula	Palearctic	337	1
Odocoileus virginianus	Nearctic	350	1
Herpestes auropunctatus	Oceanian	361	1
Capra aegagrus	Palearctic	381	1
Crocidura suaveolens	Palearctic	384	1
Myocastor coypus	Saharo-Arabian	412	1
Castor fiber	Palearctic	438	1
Lepus capensis	Palearctic	445	1
Crocidura pachyura	Palearctic	457	1
Hemitragus jemlahicus	Australian	489	1
Axis porcinus	Australian	495	1

Urocitellus parryii	Nearctic	497	1
Spilocuscus maculatus	Oceanian	503	1
Oryx gazella	Nearctic	516	1
Lepus timidus	Palearctic	527	1
Marmota marmota	Palearctic	539	1
Callosciurus erythraeus	Neotropical	540	1
Sus celebensis	Oceanian	543	1
Connochaetes taurinus	Afrotropical	589	1
Martes martes	Palearctic	589	1
Thylogale browni	Oceanian	692	1
Paradoxurus hermaphroditus	Oceanian	695	1
Marmota bobak	Palearctic	718	1
Eliomys quercinus	Palearctic	744	1
Viverra tangalunga	Oceanian	748	1
Castor canadensis	Nearctic	829	1
Hydropotes inermis	Palearctic	929	1
Myodes glareolus	Palearctic	1000	1
Rattus nitidus	Oceanian	1009	1
Rattus praetor	Oceanian	1048	1
Sylvilagus floridanus	Palearctic	1059	1
Vulpes vulpes	Palearctic	1063	1
Petaurus breviceps	Australian	1144	1
Lycalopex griseus	Neotropical	1169	1
Ammotragus lervia	Palearctic	1324	1
Atelerix algirus	Palearctic	1325	1
Castor canadensis	Neotropical	1330	1
Dasypus novemcinctus	Nearctic	1348	1
Rupicapra rupicapra	Australian	1380	1
Suncus etruscus	Palearctic	1410	1
Ondatra zibethicus	Neotropical	1428	1
Phalanger orientalis	Oceanian	1450	1
Mustela nivalis	Palearctic	1456	1
Mustela itatsi	Sino-Japanese	1457	1
Ondatra zibethicus	Nearctic	1547	1
Mustela putorius	Palearctic	1581	1
Rattus tanezumi	Afrotropical	2092	1
Mustela sibirica	Sino-Japanese	2214	1
Myodes gapperi	Nearctic	2287	1
Sorex cinereus	Nearctic	2287	1
Neovison vison	Nearctic	2291	1
Bandicota indica	Oriental	2382	1
Alces alces	Nearctic	2479	1
Dama dama	Afrotropical	2618	1
Sciurus vulgaris	Palearctic	2793	1
Erinaceus europaeus	Palearctic	3076	1
Sciurus carolinensis	Nearctic	3148	1
Muntiacus reevesi	Palearctic	3236	1
Tamiasciurus hudsonicus	Nearctic	3565	1
Hystrix cristata	Palearctic	3667	1
Tragelaphus angasii	Afrotropical	4038	1
Myocastor coypus	Sino-Japanese	4052	1
Erinaceus europaeus	Australian	4620	1
Mustela erminea	Australian	4620	1
Mustela nivalis	Australian	4620	1
Mustela putorius	Australian	4620	1

Rattus tanezumi	Oceanian	4637	1
Trichosurus vulpecula	Australian	4681	1
Paguma larvata	Sino-Japanese	4825	1
Sciurus carolinensis	Palearctic	5299	1
Potamochoerus larvatus	Madagascan	5466	1
Suncus etruscus	Madagascan	5836	1
Apodemus sylvaticus	Palearctic	6073	1
Procyon lotor	Sino-Japanese	6132	1
Ovibos moschatus	Nearctic	7436	1
Viverricula indica	Madagascan	7712	1
Suncus murinus	Madagascan	7713	1
Rusa timorensis	Oceanian	7821	1
Oreamnos americanus	Nearctic	7974	1
Bubalus bubalis	Oceanian	10417	1
Genetta genetta	Palearctic	18495	1
Rattus exulans	Oceanian	23768	1

Table S1.3. Cases excluded from niche analyses due to insufficient number of presences (< 5) in the native and/or the alien range.

**Species**	**Native**	**Realm**	**Alien**
Callosciurus finlaysonii	9366	Oriental	1
Cephalophus adersi	99	Afrotropical	2
Chlorocebus sabaeus	18612	Nearctic	1
Cricetomys gambianus	50060	Nearctic	2
Dama dama	1864	Oceanian	2
Dasyurus hallucatus	4806	Oceanian	3
Daubentonia madagascariensis	1951	Madagascan	3
Didelphis aurita	18826	Neotropical	2
Eulemur albifrons	292	Madagascan	2
Funambulus pennantii	38192	Australian	3
Funambulus pennantii	38192	Panamanian	2
Gazella subgutturosa	110399	Palearctic	2
Gazella subgutturosa	110399	Saharo-Arabian	4
Geocapromys ingrahami	2	Panamanian	2
Herpestes edwardsii	56842	Saharo-Arabian	2
Hydrochoerus hydrochaeris	163652	Neotropical	2
Lagorchestes hirsutus	10	Australian	2
Lagostrophus fasciatus	10	Australian	3
Lama glama	25880	Palearctic	2
Lama guanicoe	25880	Neotropical	2
Lasiorhinus latifrons	1174	Australian	3
Leggadina lakedownensis	17070	Australian	2
Leporillus conditor	2	Australian	6
Macaca mulatta	99601	Nearctic	2
Macaca sylvanus	744	Saharo-Arabian	1
Macropus fuliginosus	23076	Australian	2
Macropus parma	816	Australian	4
Nasua nasua	150711	Neotropical	4
Parantechinus apicalis	61	Australian	2
Peromyscus fraterculus	2264	Nearctic	3
Petrogale penicillata	1529	Australian	4
Petrogale persephone	77	Australian	1
Petrogale rothschildi	1916	Australian	4
Piliocolobus kirkii	34	Afrotropical	1
Potorous gilbertii	3	Australian	2

Pseudomys fieldi	3	Australian	4
Sapajus apella	40572	Neotropical	4
Sapajus nigritus	11663	Neotropical	2
Sciurus aureogaster	8047	Nearctic	2
Sciurus granatensis	12488	Panamanian	4
Sciurus vulgaris	442329	Sino-Japanese	1
Sundasciurus juvencus	159	Oriental	1
Tamandua tetradactyla	154928	Neotropical	2
Tragulus nigricans	16	Oriental	3
Varecia variegata	247	Madagascan	2
Wallabia bicolor	17041	Australian	4

1.2 Analysis of niche changes

To perform the niche dynamics analyses, we used the 19 bioclimatic variables from the CHELSA database (Karger et al., 2017). We resampled the original 30 arc-second rasters to 5 arc minute resolution using the "aggregate" function from *terra* (Hijmans, Bivand, et al., 2024), specifying mean as the summary option. We implemented a loop in R to process each species within each realm iteratively. To evaluate niche dynamics, we conducted a Principal Component Analysis (PCA) using the "dudi.pca" function from the *ade4* R package on bioclimatic variables for the native ranges and realm-specific alien ranges. We then applied the "ecospat.grid.clim.dyn" function from the *ecospat* R package (Broennimann et al., 2024; Di Cola et al., 2017) to create dynamic grids representing the native and alien niches, enabling us to quantify niche dynamics in terms of alien niche expansion and stability and native niche unfilling. Finally, we calculated niche dynamics metrics using "ecospat.niche.dyn.index", setting intersection = 0 to exclude novel climates.

1.2.1 Novel climates assessment

We quantified the prevalence of novel climates in alien backgrounds relative to native backgrounds using an Extrapolation Detection (ExDet) analysis (Mesgaran et al., 2014) implemented in ecospat. For each species-realm combination, we extracted background-specific climate data by cropping bioclimatic PCA rasters to the native and alien backgrounds with terra (Hijmans, Bivand, et al., 2024) and then quantified climate novelty with the "ecospat.climan" function. ExDet distinguishes two forms of novelty: Type 1, where one or more variables in alien-background cells fall outside the range observed in the native background (values < 0), and Type 2, where alien-background cells show novel multivariate combinations within the native univariate ranges (values > 1). For each alien background, we counted cells classified as novel,

calculated their proportional extent, and recorded median novelty values. In most cases, Type 1 novelty exceeded 5% in only 19 of 337 species-by-realm comparisons, while Type 2 novelty was exceedingly rare (< 0.1%; Table S1.4; Figure S1.2).

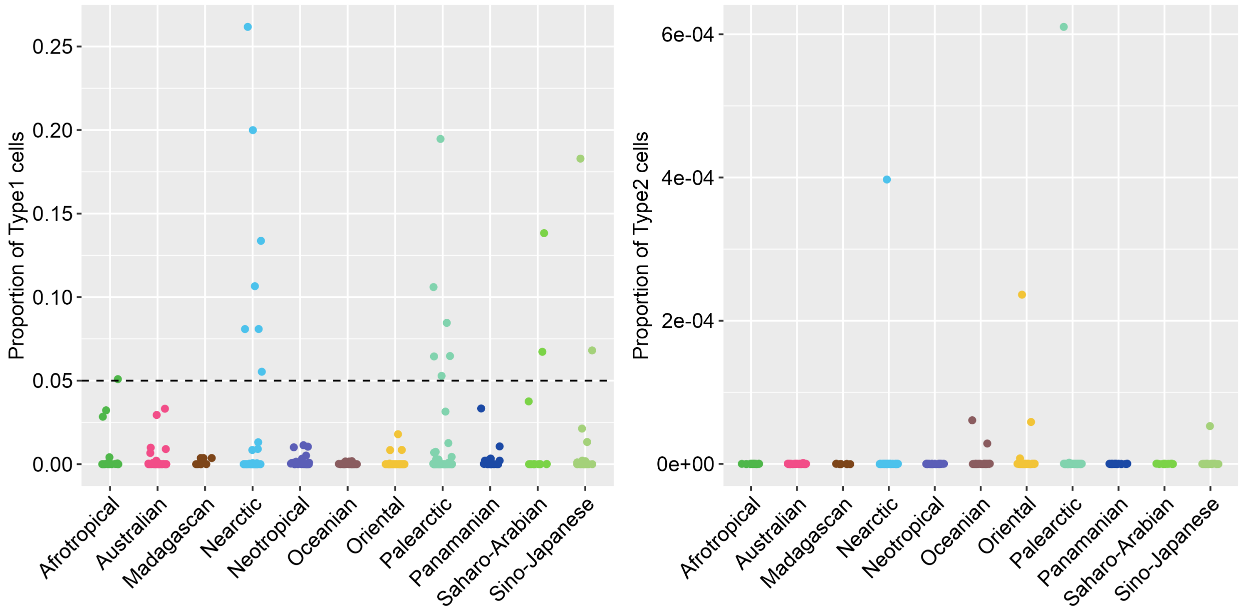

Figure S1.2: The proportion of cells in each species' alien background showing Type 1 (left) and Type 2 (right) climate novelty across zoogeographic realms, based on the Extrapolation Detection analysis. The dashed line indicates a 5% threshold.

1.2.2 Phylogenetic signal

We tested for phylogenetic signal in niche expansion to evaluate whether closely related species tended to show similar expansion values. We extracted 1000 mammal phylogenies from the Phylacine 1.2 database (Faurby et al., 2018) using the *ape* 5.6-2 R package (Paradis et al., 2024). Because some species were introduced to multiple realms, we collapsed the data to a single value per species by retaining the maximum expansion observed across realms, as phylogenetic signal testing does not allow for duplicated entries. From the posterior sample of pruned trees, we derived a maximum clade credibility (MCC) tree with the *phangorn* 2.10 R package (Schliep et al., 2024) by selecting the single posterior tree with the highest overall clade support, defined as the tree maximizing the sum of posterior probabilities of its constituent clades. The MCC retains internally consistent branch lengths and provides a representative phylogenetic hypothesis for subsequent analyses. We then estimated Pagel's λ for niche expansion using "phylosig" from the *phytools* 2.4-4 R package (Revell, 2024), where λ ranges from 0 (no phylogenetic signal) to 1

(strong signal) (Münkemüller et al., 2012). The estimated λ was 0.090 and the likelihood-ratio test against λ = 0 was not significant (p = 0.129), indicating no detectable phylogenetic signal.

Table S1.4: The proportion of cells in each species' alien background showing Type 1 and Type 2 climate novelty across zoogeographic realms, based on the Extrapolation Detection analysis

**Binomial**	**Realm**	**Type 1**	**Type 2**
Beatragus hunteri	Afrotropical	0.000	0.000
Cercopithecus mona	Afrotropical	0.000	0.000
Chlorocebus sabaeus	Afrotropical	0.000	0.000
Civettictis civetta	Afrotropical	0.000	0.000
Connochaetes taurinus	Afrotropical	0.000	0.000
Dama dama	Afrotropical	0.051	0.000
Hemitragus jemlahicus	Afrotropical	0.000	0.000
Herpestes auropunctatus	Afrotropical	0.000	0.000
Mustela nivalis	Afrotropical	0.000	0.000
Myocastor coypus	Afrotropical	0.032	0.000
Nanger soemmerringii	Afrotropical	0.000	0.000
Rattus tanezumi	Afrotropical	0.000	0.000
Rusa unicolor	Afrotropical	0.000	0.000
Sciurus carolinensis	Afrotropical	0.004	0.000
Suncus murinus	Afrotropical	0.028	0.000
Tragelaphus angasii	Afrotropical	0.000	0.000
Viverricula indica	Afrotropical	0.000	0.000
Axis axis	Australian	0.000	0.000
Axis porcinus	Australian	0.000	0.000
Bettongia lesueur	Australian	0.000	0.000
Bettongia penicillata	Australian	0.000	0.000
Bos javanicus	Australian	0.000	0.000
Bubalus bubalis	Australian	0.000	0.000
Camelus dromedarius	Australian	2.24E-05	0.000
Cervus canadensis	Australian	3.64E-06	0.000
Cervus elaphus	Australian	8.02E-05	0.000
Cervus nippon	Australian	0.000	0.000
Dama dama	Australian	0.010	0.000
Erinaceus europaeus	Australian	0.000	0.000
Hemitragus jemlahicus	Australian	0.029	0.000
Isoodon obesulus	Australian	0.000	0.000
Lepus europaeus	Australian	0.001	1.18E-06
Macropus agilis	Australian	0.000	0.000
Macropus eugenii	Australian	0.000	0.000
Macropus giganteus	Australian	0.000	0.000
Macropus rufogriseus	Australian	0.000	0.000
Mustela erminea	Australian	3.64E-06	0.000
Mustela nivalis	Australian	3.64E-06	0.000
Mustela putorius	Australian	0.000	0.000
Odocoileus virginianus	Australian	0.000	0.000
Ornithorhynchus anatinus	Australian	0.000	0.000
Oryctolagus cuniculus	Australian	0.002	0.000

Petaurus breviceps	Australian	0.000	0.000
Petrogale lateralis	Australian	0.007	0.000
Phascolarctos cinereus	Australian	0.000	0.000
Pseudocheirus peregrinus	Australian	0.000	0.000
Rattus exulans	Australian	0.009	0.000
Rupicapra rupicapra	Australian	0.000	0.000
Rusa timorensis	Australian	0.033	0.000
Rusa unicolor	Australian	0.000	0.000
Sarcophilus harrisii	Australian	0.000	0.000
Tachyglossus aculeatus	Australian	0.000	0.000
Thylogale billardierii	Australian	0.000	0.000
Trichosurus vulpecula	Australian	0.000	0.000
Vombatus ursinus	Australian	0.000	0.000
Vulpes vulpes	Australian	1.18E-06	0.000
Eulemur fulvus	Madagascan	0.004	0.000
Eulemur mongoz	Madagascan	0.004	0.000
Herpestes auropunctatus	Madagascan	0.000	0.000
Potamochoerus larvatus	Madagascan	0.000	0.000
Suncus etruscus	Madagascan	0.000	0.000
Suncus murinus	Madagascan	1.91E-05	0.000
Tenrec ecaudatus	Madagascan	0.004	0.000
Viverricula indica	Madagascan	1.91E-05	0.000
Alces alces	Nearctic	0.000	0.000
Ammotragus lervia	Nearctic	0.134	0.000
Antilope cervicapra	Nearctic	0.262	0.000
Axis axis	Nearctic	0.009	0.000
Bison bison	Nearctic	0.000	0.000
Boselaphus tragocamelus	Nearctic	0.200	0.000
Castor canadensis	Nearctic	0.000	0.000
Cervus canadensis	Nearctic	0.000	0.000
Cervus elaphus	Nearctic	0.000	0.000
Cervus nippon	Nearctic	0.000	0.000
Dama dama	Nearctic	0.081	0.000
Dasypus novemcinctus	Nearctic	0.000	0.000
Didelphis virginiana	Nearctic	0.000	0.000
Herpestes auropunctatus	Nearctic	0.001	0.000
Lepus americanus	Nearctic	0.000	0.000
Lepus arcticus	Nearctic	0.000	0.000
Lepus californicus	Nearctic	0.000	0.000
Lepus europaeus	Nearctic	0.000	0.000
Martes americana	Nearctic	0.000	0.000
Martes foina	Nearctic	0.000	0.000
Microtus californicus	Nearctic	0.000	0.000
Myocastor coypus	Nearctic	0.055	0.000
Myodes gapperi	Nearctic	0.000	0.000
Neovison vison	Nearctic	0.000	0.000
Odocoileus hemionus	Nearctic	7.14E-05	0.000
Odocoileus virginianus	Nearctic	0.000	0.000
Ondatra zibethicus	Nearctic	0.000	0.000
Oreamnos americanus	Nearctic	0.000	0.000

Oryctolagus cuniculus	Nearctic	0.107	0.000
Oryx gazella	Nearctic	0.081	0.000
Ovibos moschatus	Nearctic	0.000	0.000
Ovis canadensis	Nearctic	0.000	0.000
Ovis orientalis	Nearctic	0.001	0.000
Peromyscus maniculatus	Nearctic	0.000	0.000
Petrogale penicillata	Nearctic	0.008	0.000
Procyon lotor	Nearctic	0.000	0.000
Rangifer tarandus	Nearctic	0.000	0.000
Rattus exulans	Nearctic	0.013	0.000
Rusa unicolor	Nearctic	0.000	0.000
Sciurus aberti	Nearctic	0.000	0.000
Sciurus carolinensis	Nearctic	0.000	0.000
Sciurus niger	Nearctic	0.000	0.000
Sorex cinereus	Nearctic	0.000	0.000
Sylvilagus floridanus	Nearctic	0.000	0.000
Tamias striatus	Nearctic	0.000	0.000
Tamiasciurus hudsonicus	Nearctic	0.000	0.000
Urocitellus parryii	Nearctic	0.000	0.000
Vulpes lagopus	Nearctic	0.000	0.000
Vulpes vulpes	Nearctic	0.000	0.000
Antilope cervicapra	Neotropical	0.010	0.000
Axis axis	Neotropical	0.001	0.000
Bubalus bubalis	Neotropical	0.001	0.000
Callithrix geoffroyi	Neotropical	0.000	0.000
Callithrix jacchus	Neotropical	0.001	0.000
Callithrix penicillata	Neotropical	0.000	0.000
Callosciurus erythraeus	Neotropical	0.000	0.000
Castor canadensis	Neotropical	0.000	0.000
Cercopithecus mona	Neotropical	0.011	0.000
Cervus elaphus	Neotropical	0.001	0.000
Chaetophractus villosus	Neotropical	0.000	0.000
Dama dama	Neotropical	0.010	0.000
Dasyprocta leporina	Neotropical	0.000	0.000
Dasypus novemcinctus	Neotropical	0.000	0.000
Didelphis marsupialis	Neotropical	0.000	0.000
Herpestes auropunctatus	Neotropical	0.001	0.000
Hippopotamus amphibius	Neotropical	0.001	0.000
Lepus europaeus	Neotropical	0.003	0.000
Lycalopex griseus	Neotropical	0.000	0.000
Neovison vison	Neotropical	0.000	0.000
Odocoileus virginianus	Neotropical	0.000	0.000
Ondatra zibethicus	Neotropical	0.000	0.000
Oryctolagus cuniculus	Neotropical	0.005	0.000
Rangifer tarandus	Neotropical	0.000	0.000
Saguinus oedipus	Neotropical	0.000	0.000
Saimiri sciureus	Neotropical	0.000	0.000
Sciurus stramineus	Neotropical	0.000	0.000
Vicugna vicugna	Neotropical	0.001	0.000
Babyrousa babyrussa	Oceanian	0.000	0.000

Bubalus bubalis	Oceanian	0.000	0.000
Crocidura maxi	Oceanian	0.000	0.000
Crocidura monticola	Oceanian	0.000	0.000
Dendrolagus matschiei	Oceanian	0.000	0.000
Herpestes auropunctatus	Oceanian	0.000	0.000
Herpestes fuscus	Oceanian	0.002	6.11E-05
Lepus nigricollis	Oceanian	1.84E-05	0.000
Macaca fascicularis	Oceanian	0.000	0.000
Macaca nigra	Oceanian	0.000	0.000
Macropus agilis	Oceanian	0.000	0.000
Oryctolagus cuniculus	Oceanian	0.002	0.000
Paradoxurus hermaphroditus	Oceanian	0.000	0.000
Petaurus breviceps	Oceanian	0.000	0.000
Phalanger orientalis	Oceanian	0.000	0.000
Rangifer tarandus	Oceanian	0.002	0.000
Rattus argentiventer	Oceanian	0.000	0.000
Rattus exulans	Oceanian	5.49E-05	0.000
Rattus nitidus	Oceanian	0.000	0.000
Rattus praetor	Oceanian	0.000	0.000
Rattus tanezumi	Oceanian	0.000	0.000
Rusa marianna	Oceanian	0.000	0.000
Rusa timorensis	Oceanian	0.000	0.000
Spilocuscus maculatus	Oceanian	0.000	0.000
Suncus murinus	Oceanian	4.66E-06	0.000
Sus celebensis	Oceanian	0.000	0.000
Tenrec ecaudatus	Oceanian	0.001	2.83E-05
Thylogale browni	Oceanian	0.000	0.000
Thylogale brunii	Oceanian	0.000	0.000
Viverra tangalunga	Oceanian	0.000	0.000
Axis axis	Oriental	0.000	0.000
Axis porcinus	Oriental	0.000	0.000
Bandicota indica	Oriental	0.000	0.000
Bos javanicus	Oriental	0.000	0.000
Bubalus bubalis	Oriental	0.000	0.000
Callosciurus notatus	Oriental	0.000	0.000
Callosciurus prevostii	Oriental	0.000	0.000
Cervus nippon	Oriental	0.000	0.000
Elephas maximus	Oriental	0.000	0.000
Funambulus pennantii	Oriental	0.000	0.000
Herpestes auropunctatus	Oriental	0.000	0.000
Hystrix javanica	Oriental	0.000	0.000
Lepus nigricollis	Oriental	0.000	0.000
Macaca fascicularis	Oriental	0.000	0.000
Macaca leonina	Oriental	0.000	0.000
Macaca nemestrina	Oriental	0.000	0.000
Manis culionensis	Oriental	0.000	0.000
Muntiacus muntjak	Oriental	0.000	0.000
Mus caroli	Oriental	0.000	0.000
Mus terricolor	Oriental	0.000	0.000

Mustela itatsi	Oriental	0.018	0.000
Paradoxurus hermaphroditus	Oriental	0.000	0.000
Phalanger orientalis	Oriental	0.008	5.83E-05
Rattus argentiventer	Oriental	0.000	0.000
Rattus exulans	Oriental	0.000	0.000
Rattus nitidus	Oriental	6.42E-06	0.000
Rattus tanezumi	Oriental	6.42E-06	0.000
Rusa timorensis	Oriental	6.42E-06	0.000
Semnopithecus entellus	Oriental	0.000	0.000
Spilocuscus maculatus	Oriental	0.000	0.000
Suncus murinus	Oriental	0.000	0.000
Sus celebensis	Oriental	0.009	7.58E-06
Trachypithecus auratus	Oriental	0.000	0.000
Viverra tangalunga	Oriental	0.000	0.000
Viverra zibetha	Oriental	0.000	0.000
Viverricula indica	Oriental	0.000	0.000
Ammotragus lervia	Palearctic	0.031	0.001
Apodemus sylvaticus	Palearctic	0.003	0.000
Atelerix algirus	Palearctic	0.000	0.000
Axis axis	Palearctic	0.000	0.000
Callosciurus erythraeus	Palearctic	0.000	0.000
Callosciurus finlaysonii	Palearctic	0.195	0.000
Capra aegagrus	Palearctic	0.000	0.000
Capra ibex	Palearctic	0.000	0.000
Capra sibirica	Palearctic	0.000	0.000
Capreolus capreolus	Palearctic	0.000	0.000
Capreolus pygargus	Palearctic	0.000	0.000
Castor canadensis	Palearctic	0.000	0.000
Castor fiber	Palearctic	0.000	0.000
Cervus canadensis	Palearctic	0.000	0.000
Cervus elaphus	Palearctic	0.000	0.000
Cervus nippon	Palearctic	3.50E-05	0.000
Crocidura dsinezumi	Palearctic	0.000	0.000
Crocidura pachyura	Palearctic	0.000	0.000
Crocidura russula	Palearctic	0.000	0.000
Crocidura suaveolens	Palearctic	0.000	0.000
Dama dama	Palearctic	0.106	0.000
Desmana moschata	Palearctic	7.85E-05	0.000
Eliomys quercinus	Palearctic	0.000	0.000
Erinaceus europaeus	Palearctic	0.001	0.000
Erinaceus roumanicus	Palearctic	0.000	0.000
Eutamias sibiricus	Palearctic	0.000	0.000
Genetta genetta	Palearctic	0.007	0.000
Glis glis	Palearctic	0.000	0.000
Herpestes auropunctatus	Palearctic	0.000	0.000
Hydropotes inermis	Palearctic	0.003	0.000
Hystrix cristata	Palearctic	0.000	0.000
Lepus capensis	Palearctic	0.000	0.000
Lepus corsicanus	Palearctic	0.000	0.000

Lepus europaeus	Palearctic	0.000	0.000
Lepus granatensis	Palearctic	0.000	0.000
Lepus timidus	Palearctic	0.000	0.000
Macropus rufogriseus	Palearctic	0.064	0.000
Marmota bobak	Palearctic	0.000	0.000
Marmota marmota	Palearctic	0.000	0.000
Martes foina	Palearctic	0.000	0.000
Martes martes	Palearctic	0.000	0.000
Martes zibellina	Palearctic	0.004	0.000
Meles meles	Palearctic	0.000	0.000
Micromys minutus	Palearctic	0.000	0.000
Microtus arvalis	Palearctic	0.000	0.000
Microtus levis	Palearctic	0.000	0.000
Muntiacus reevesi	Palearctic	0.053	2.10E-06
Mus spretus	Palearctic	0.000	0.000
Mustela erminea	Palearctic	0.000	0.000
Mustela lutreola	Palearctic	0.000	0.000
Mustela nivalis	Palearctic	0.000	0.000
Mustela putorius	Palearctic	0.000	0.000
Myocastor coypus	Palearctic	0.065	0.000
Myodes glareolus	Palearctic	0.000	0.000
Myodes rutilus	Palearctic	0.000	0.000
Nasua nasua	Palearctic	0.000	0.000
Neovison vison	Palearctic	0.000	0.000
Nyctereutes procyonoides	Palearctic	3.50E-05	0.000
Odocoileus virginianus	Palearctic	0.000	0.000
Ondatra zibethicus	Palearctic	0.001	0.000
Oryctolagus cuniculus	Palearctic	0.085	0.000
Ovibos moschatus	Palearctic	0.000	0.000
Ovis orientalis	Palearctic	0.013	0.000
Procyon lotor	Palearctic	0.007	0.000
Rangifer tarandus	Palearctic	0.000	0.000
Rupicapra rupicapra	Palearctic	0.000	0.000
Sciurus anomalus	Palearctic	0.000	0.000
Sciurus carolinensis	Palearctic	0.000	0.000
Sciurus vulgaris	Palearctic	0.000	0.000
Suncus etruscus	Palearctic	0.000	0.000
Sylvilagus floridanus	Palearctic	0.000	0.000
Vulpes lagopus	Palearctic	3.58E-05	0.000
Vulpes vulpes	Palearctic	0.000	0.000
Antilope cervicapra	Panamanian	0.003	0.000
Boselaphus tragocamelus	Panamanian	0.003	0.000
Bubalus bubalis	Panamanian	0.002	0.000
Chlorocebus sabaeus	Panamanian	0.000	0.000
Cuniculus paca	Panamanian	0.000	0.000
Dama dama	Panamanian	0.033	0.000
Dasyprocta leporina	Panamanian	0.000	0.000
Dasyprocta mexicana	Panamanian	0.002	0.000
Dasyprocta punctata	Panamanian	0.000	0.000
Dasypus novemcinctus	Panamanian	0.000	0.000

Didelphis marsupialis	Panamanian	0.000	0.000
Erythrocebus patas	Panamanian	0.000	0.000
Herpestes auropunctatus	Panamanian	0.000	0.000
Lepus europaeus	Panamanian	0.011	0.000
Macaca arctoides	Panamanian	0.002	0.000
Macaca mulatta	Panamanian	0.002	0.000
Odocoileus virginianus	Panamanian	0.000	0.000
Oryctolagus cuniculus	Panamanian	0.000	0.000
Ovis orientalis	Panamanian	0.000	0.000
Procyon lotor	Panamanian	0.000	0.000
Sylvilagus floridanus	Panamanian	0.000	0.000
Tragelaphus derbianus	Panamanian	0.000	0.000
Apodemus sylvaticus	Saharo-Arabian	0.000	0.000
Atelerix algirus	Saharo-Arabian	0.000	0.000
Atlantoxerus getulus	Saharo-Arabian	0.000	0.000
Bubalus bubalis	Saharo-Arabian	0.000	0.000
Crocidura pachyura	Saharo-Arabian	0.000	0.000
Funambulus pennantii	Saharo-Arabian	0.000	0.000
Lepus europaeus	Saharo-Arabian	0.000	0.000
Mustela nivalis	Saharo-Arabian	0.000	0.000
Myocastor coypus	Saharo-Arabian	0.038	0.000
Oryctolagus cuniculus	Saharo-Arabian	0.138	0.000
Ovis orientalis	Saharo-Arabian	0.000	0.000
Suncus etruscus	Saharo-Arabian	0.000	0.000
Suncus murinus	Saharo-Arabian	0.067	0.000
Vulpes vulpes	Saharo-Arabian	0.000	0.000
Callosciurus erythraeus	Sino-Japanese	0.000	0.000
Callosciurus finlaysonii	Sino-Japanese	0.002	0.000
Crocidura dsinezumi	Sino-Japanese	0.000	0.000
Eutamias sibiricus	Sino-Japanese	0.000	0.000
Herpestes auropunctatus	Sino-Japanese	0.000	0.000
Macaca cyclopis	Sino-Japanese	0.183	0.000
Macaca fascicularis	Sino-Japanese	0.002	0.000
Macaca mulatta	Sino-Japanese	0.000	0.000
Martes melampus	Sino-Japanese	0.000	0.000
Muntiacus reevesi	Sino-Japanese	0.021	5.28E-05
Mustela itatsi	Sino-Japanese	0.000	0.000
Mustela lutreola	Sino-Japanese	0.000	0.000
Mustela sibirica	Sino-Japanese	0.000	0.000
Myocastor coypus	Sino-Japanese	0.068	0.000
Neovison vison	Sino-Japanese	0.000	0.000
Nyctereutes procyonoides	Sino-Japanese	0.000	0.000
Ondatra zibethicus	Sino-Japanese	0.000	0.000
Oryctolagus cuniculus	Sino-Japanese	0.001	0.000
Paguma larvata	Sino-Japanese	0.013	0.000
Procyon lotor	Sino-Japanese	0.000	0.000
Suncus murinus	Sino-Japanese	0.000	0.000

1.3 Variables statistics

The Fast-growth index (Figure S1.3) spanned from -14.713 for *Elephas maximus* in the Oriental realm to 4.724 for *Suncus murinus* in the Afrotropical realm (0.326 ± 2.283). Dispersal distance (Figure S1.4) ranged from 0.070 km for *Micromys minutus* in the Palearctic realm to 90.080 km for *Elephas maximus* in the Oriental realm (2.689 ± 10.090). Native range size (Figure S1.5) extended from 983.312 km² for *Bettongia lesueur* in the Australian realm to 59,763,903.000 km² for *Vulpes vulpes* in the Australian realm (3,119,322.000 ± 9,263,143.000). The Specialization index (Figure S1.6) ranged from -0.322 for *Odocoileus virginianus* in the Australian realm to 4.605 for *Camelus dromedarius* in the Australian realm (1.897 ± 0.938). Native range loss (Figure S1.7), measured in grid cells, had a minimum of 0.000 for *Apodemus sylvaticus* in the Palearctic realm and a maximum of 2545.000 for *Camelus dromedarius* in the Australian realm (0.000 ± 386.478). Introduction effort (Figure S1.8) varied from 1.000 for *Apodemus sylvaticus* in the Saharo-Arabian realm to 36.249 for *Oryctolagus cuniculus* in the Palearctic realm (median with standard deviation = 2.000 ± 3.096). Residence time (Figure S1.9) varied from 9.000 years for *Sarcophilus harrisii* in the Australian realm to 23,521,000 years for *Phalanger orientalis* in the Oceanian realm (137.000 ± 2,891.777). Out of 337 alien ranges, 214 were restricted to islands (Figure S1.10). Human disturbance (Figure S1.11) ranged from 0.000 for *Ovibos moschatus* in the Nearctic realm to 46.002 for *Macaca fascicularis* in the Sino-Japanese realm (11.814 ± 8.546). Native mammal richness Figure S1.12) varied from 0.000 species for *Rangifer tarandus* in the Neotropical realm to 123.108 species for *Herpestes auropunctatus* in the Neotropical realm (19.478 ± 22.465). Community similarity (Figure S1.13) ranged from 0 for *Ammotragus lervia* in the Nearctic realm to 1 for *Alces alces* in the same realm (0.333 ± 0.461).

Table S1.5. Variance Inflation Factor values for all variables.

**Variables**	**VIF**
Dispersal	2.561
Fast-growth index	2.183
Alien insularity	1.959
Native mammal richness	1.802
Native range loss	1.729
Residence time	1.365
Introduction effort	1.220
Native range size	1.195
Human disturbance	1.193
Community similarity	1.102
Specialization index	1.070

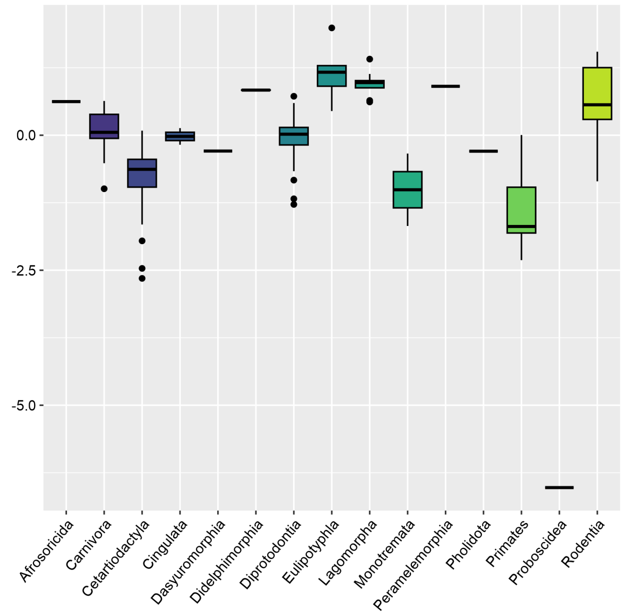

Figure S1.3: Scaled values of the Fast-growth index across orders.

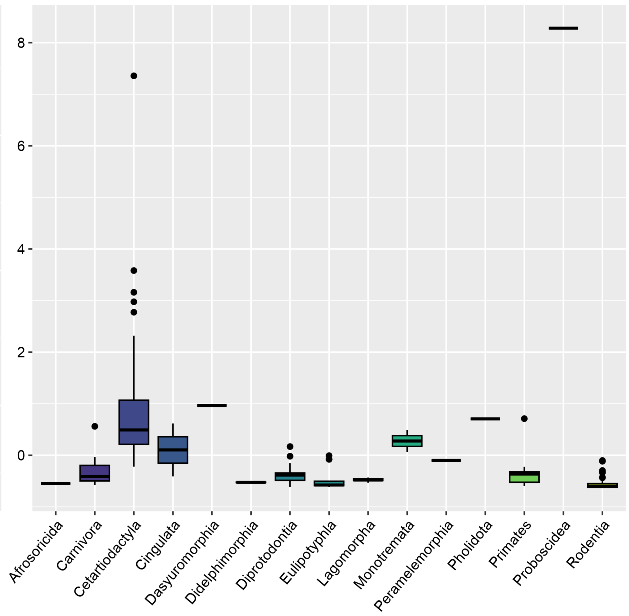

Figure S1.4: Scaled values of Dispersal across orders.

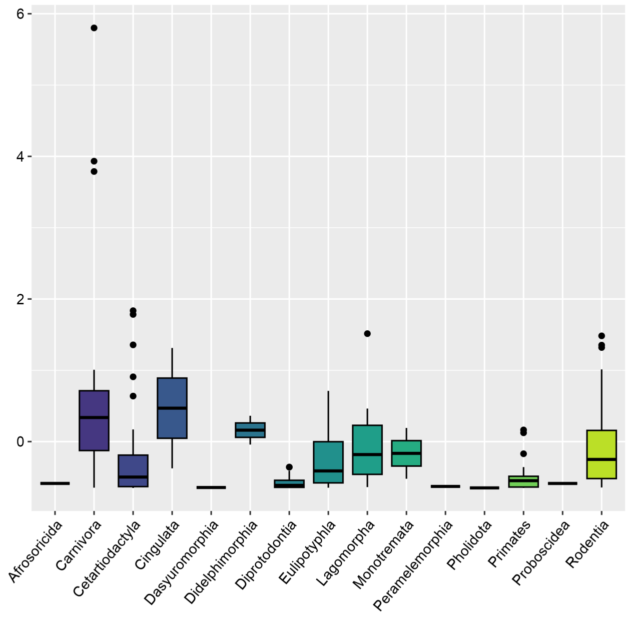

Figure S1.5: Scaled values of Native ranges sizes across orders.

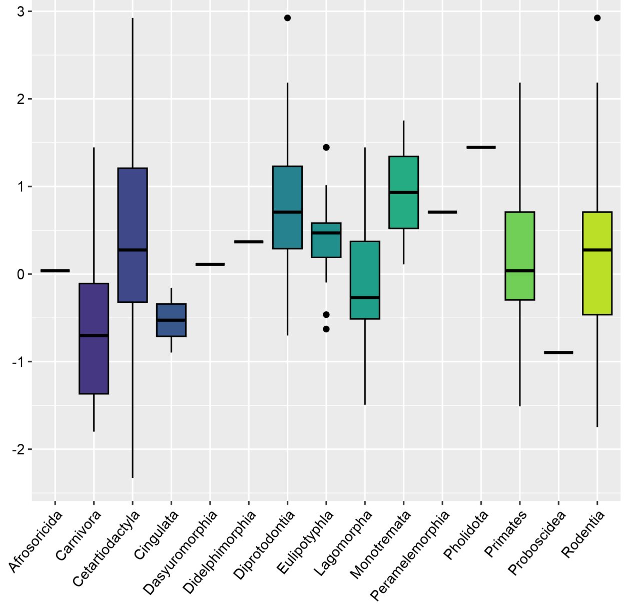

Figure S1.6: Scaled values of the Specialization index across orders.

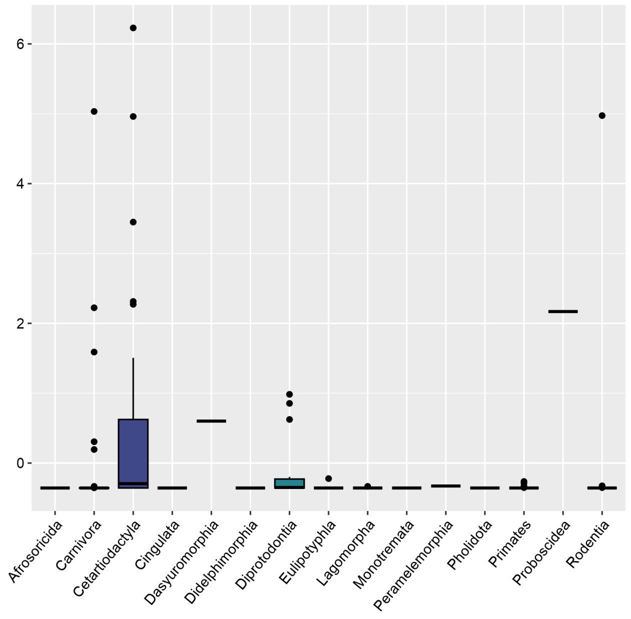

Figure S1.7: Scaled values of Native range loss across orders.

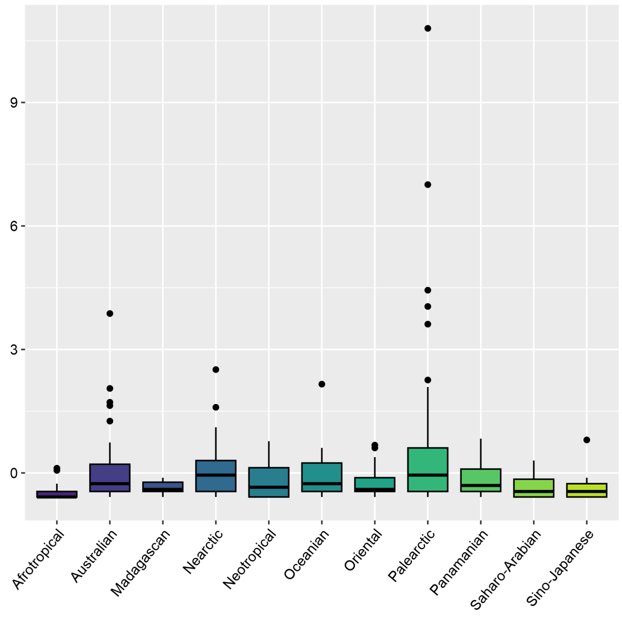

Figure S1.8: Scaled values of Introduction effort across realms.

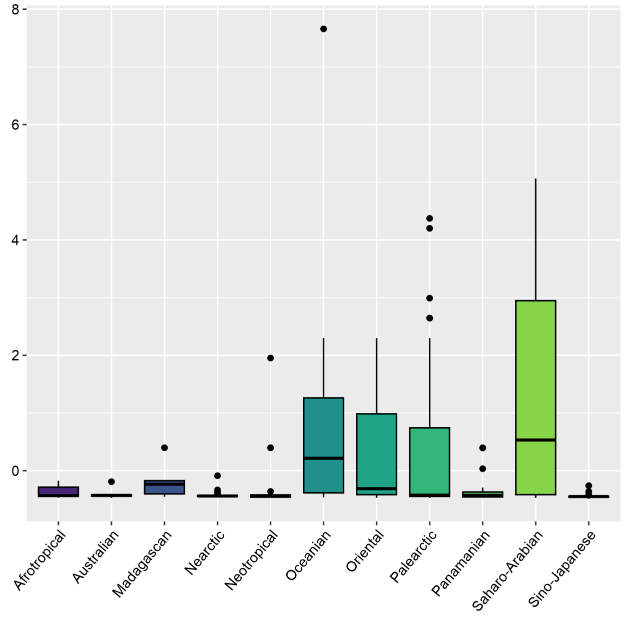

Figure S1.9: Scaled values of Residence time across realms.

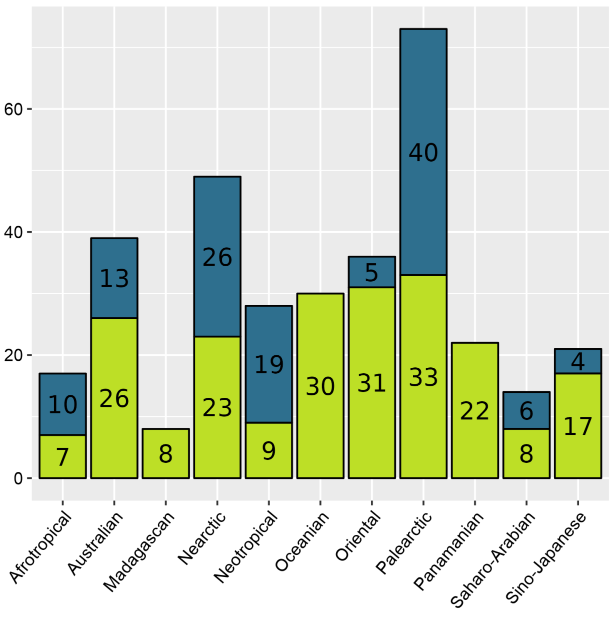

Figure S1.10: Alien insularity across realms. Alien ranges confined to islands (represented as 1) are depicted in green, while those extending to both islands and the mainland (represented as 0) are shown in blue.

Global Change Biology

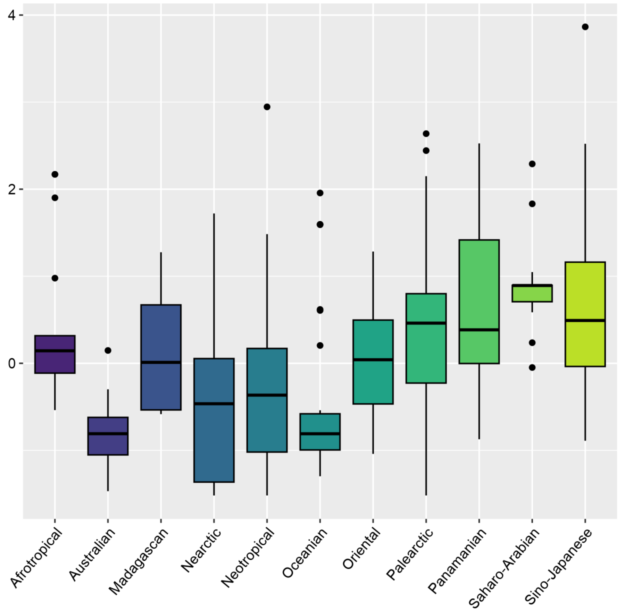

Figure S1.11: Scaled values of Human disturbance across realms.

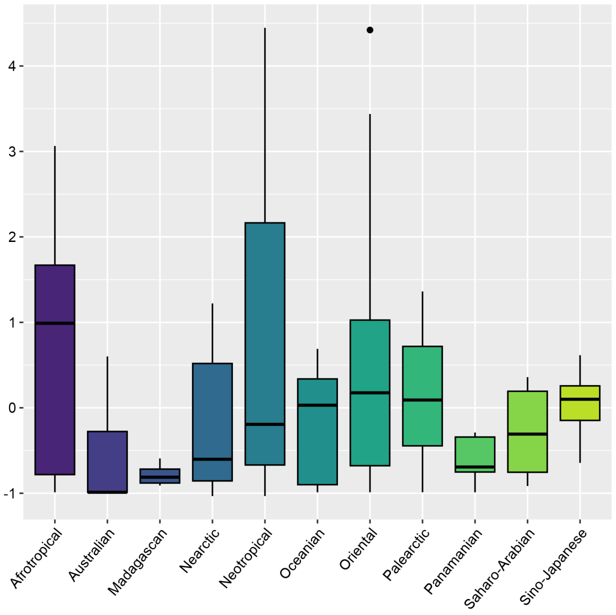

Figure S1.12: Scaled values of Native mammal richness across realms.

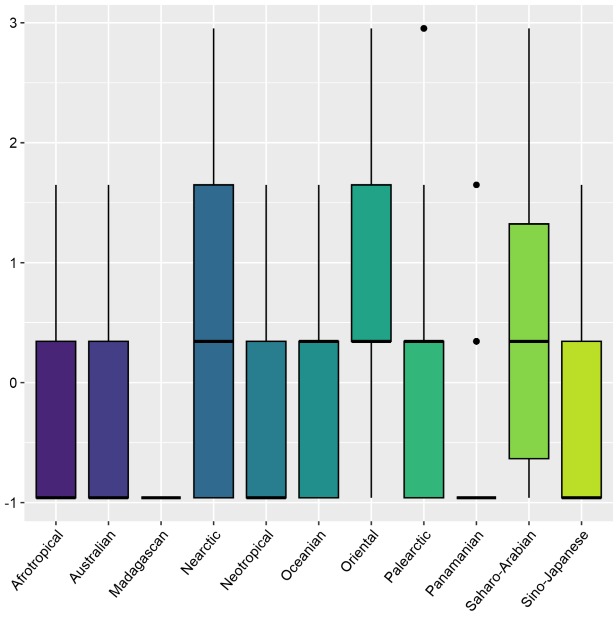

Figure S1.13: Scaled values of Community similarity across realms.

1.4 Transformations of niche changes and their drivers

We examined the distribution of the response variables using the “descdist” function from the *fitdistrplus* R package (Delignette-Muller et al., 2024). All distributions depart markedly from normality, showing skewness and kurtosis values outside the Gaussian expectation (skewness = 0, kurtosis = 3) (Zuur et al., 2007, 2009). Niche unfilling ranged from 0 to 1 and showed a median of 0.766 (Standard deviation = ± 0.330); its skewness of -0.700 indicated a moderate tail toward lower values, and a kurtosis of 2.13 pointed to a somewhat flatter peak, and lighter tails than a normal distribution. Niche expansion also ranged from 0 to 1 and showed a median of 0.00022 (± 0.267), reflecting that most species showed negligible expansion; its skewness of 2.382 revealed a strong right-hand tail of rare high-expansion values, and a kurtosis of 7.41 indicated a sharply peaked distribution with heavy tails.

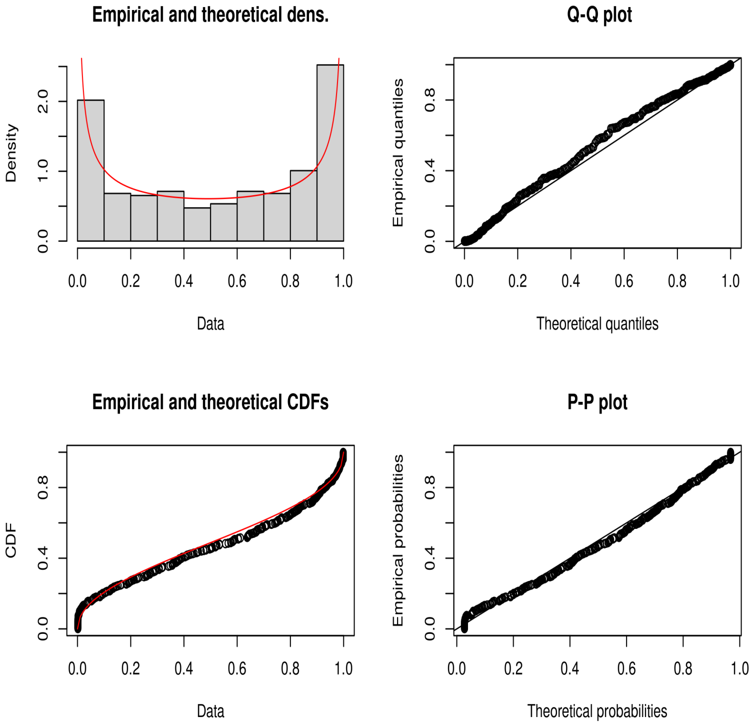

Figure S1.15: Goodness-of-fit diagnostics for the beta distribution fitted to the niche unfilling values. The plots show: (top-left) empirical histogram and theoretical beta density (red line), (top-right) Q-Q plot comparing empirical and theoretical quantiles, (bottom-left) empirical and theoretical cumulative distribution functions and (bottom-right) P-P plot comparing empirical and theoretical probabilities.

For niche expansion, we used k-means clustering with k = 2 to divide expansion values into low and high groups (Mucherino et al., 2009; Zuur et al., 2007). Observations with expansion values above the lower cluster centroid (0.035) were classified as significant expansion (1), and all others as non-significant (0). This binarization addressed skewness and yielded a response variable appropriate for binomial modeling. To prepare the niche unfilling data for modelling, we first applied the square root transformation to its values (Zuur et al., 2009). The squared values were then rescaled using the formula: (unfilling^2 × (n - 1) + 0.5) / n, where n is the sample

size (Smithson & Verkuilen, 2006). This common rescaling technique avoids boundary values of 0 and 1, which are not permitted in standard beta regression. We then fitted a beta distribution via maximum-likelihood estimation using the "fitdist" function from fitdistrplus. Diagnostic checks confirmed close agreement between transformed values and the fitted beta distribution (Figure S1.1)

1.4.1 Fixed-response variable relationships

To enhance the linearity of relationships between candidate drivers and niche-change responses and to reduce heteroscedasticity, we applied variable-specific transformations to the fixed effects included in each niche GLMM and compared models fitted with raw versus transformed covariates. For the niche-expansion model, we applied Yeo-Johnson transformations to native range loss, introduction effort, native mammal richness, and human disturbance, a Box-Cox transformation to native range size, and a log transformation to dispersal and residence time, while fast growth index, alien insularity, community similarity, and specialization index were retained on their original scale. For the niche-unfilling model, we applied a Yeo-Johnson transformation to native range loss and introduction effort, and log transformations to dispersal, native range size, native mammal richness, and human disturbance. For both models, residence time was log-transformed after adding a decade offset to account for reporting lags, because dates often reflect first detection rather than introduction events (Seebens et al., 2017) and are frequently uncertain or imprecisely reported (Biancolini et al., 2021); this offset down-weights small differences among recent introductions and stabilizes model fitting. We implemented Yeo-Johnson and Box-Cox transformations using the the *bestNormalize* R package ("yeojohnson" and "boxcox" functions) (Peterson, 2023a, 2023b) and log transformations using base R, then evaluated raw versus transformed model variants via the small sample-size corrected Akaike's Information Criterion (AICc), alongside visual inspection of bivariate response plots to check whether transformations improved the linearity and variance structure of each driver-response relationship.

We constructed bivariate response plots in the ggplot2 R package (Wickham et al., 2024) by plotting the observed response against each fixed variable and overlaying a smoothed conditional mean estimated with stat_smooth(method = "glm") using the same distribution and link as the corresponding niche-change model (beta with logit link for niche unfilling and binomial with

logit link for niche expansion). These smooths were intended as diagnostic visualizations of the marginal bivariate relationships rather than partial effects from the full GLMM.

For the niche-expansion model, the raw vs transformed response plots (Figure S1.17) indicated that transformations mainly stabilized the driver scale by reducing tail leverage and yielding smoother fitted mean relationships with uncertainty that was more evenly distributed across the observed x-range. In the raw scale, several drivers showed strong clustering near zero with sparse high-end values, so the fitted curve was disproportionately influenced by the upper tail and the confidence band expanded rapidly where data were rare. This pattern was especially evident for dispersal and residence time after log transformation, and for introduction effort and native range loss after Yeo-Johnson transformation, where the upper tail was compressed and the fitted relationships appeared less sensitive to a few extreme points. Native range size showed the clearest improvement: the raw plot was strongly curved with a long right tail, whereas the transformed plot supported a cleaner negative pattern with reduced inflation of uncertainty at high values. Introduction effort showed a similar improvement in the opposite direction, with the transformed scale supporting a more regular increase rather than an apparently strongly non-linear pattern driven by the mass of near-zero values. For native mammal richness and human disturbance, transformations largely preserved the direction of the marginal relationships but attenuated the influence of extreme values, producing slightly smoother trends and a more stable uncertainty

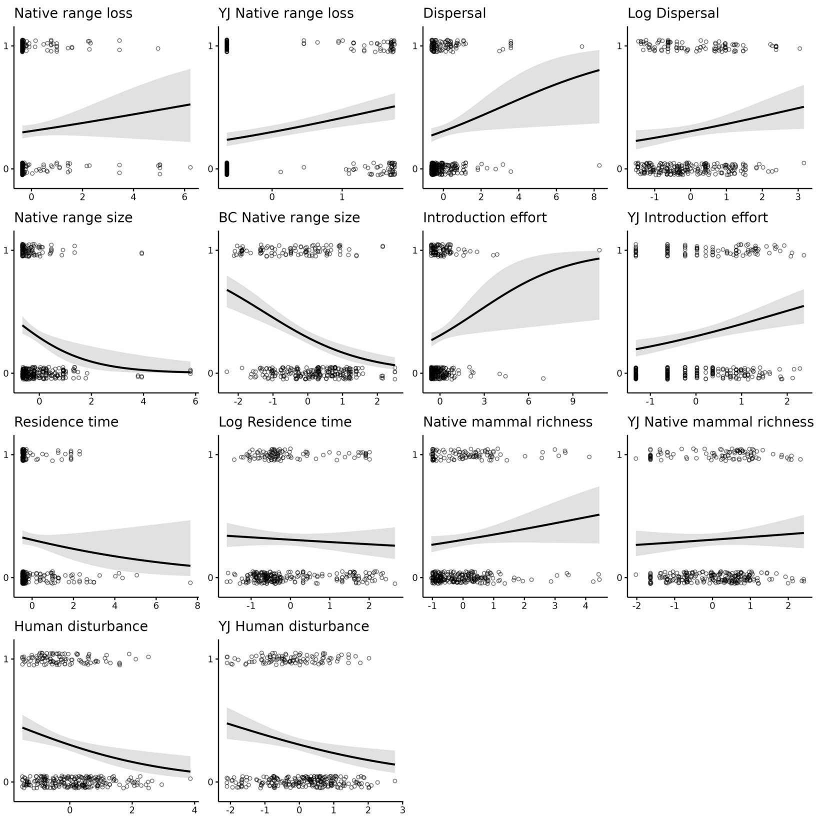

Figure S1.17: Response plots showing the relationship between niche expansion (y-axis) and seven fixed variables (x-axes) presented in both their raw and transformed version. The black line represents the modeled relationship based on the transformed variables, and the gray shaded area is the 95% confidence interval.

envelope. Overall, the patterns observed in the plots matched the model comparison: the transformed niche-expansion model was markedly better supported than the raw one, with a ΔAICc of −23.083.

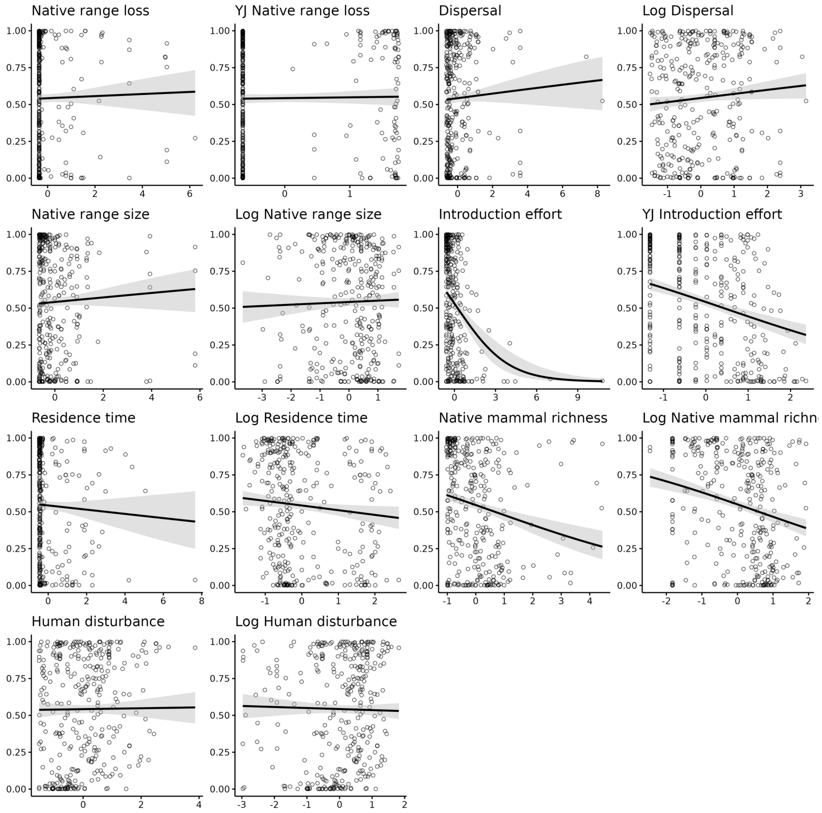

Figure S1.18: Response plots showing the relationship between niche unfilling (y-axis) and eight fixed variables (x-axes) presented in both their raw and transformed version. The black line represents the modeled relationship based on the transformed variables, and the gray shaded area is the 95% confidence interval.

Similarly, for the niche-unfilling model, the raw vs transformed plots in Figure S1.18 suggested that transformations mainly reduced the leverage of sparse extremes and made uncertainty more evenly distributed along the driver range, yielding smoother marginal fits. Introduction effort showed the clearest improvement: on the raw scale the fitted relationship was strongly non-linear, with a sharp decline at low values and rapid flattening where observations became sparse, whereas after Yeo-Johnson transformation the negative association appeared closer to linear and the confidence ribbon was less heterogeneous. Residence time showed a consistent negative association, with the log scale compressing the upper tail and reducing ribbon inflation at high values. Native range loss and dispersal showed weak positive marginal trends on both scales, but the transformed versions appeared less sensitive to the uneven raw-scale distribution. Human disturbance remained positively associated with unfilling, and the log-transformed scale yielded a smoother fitted increase with reduced sensitivity at the extremes. Native range size showed only a weak marginal association with niche unfilling on the raw scale, and after log transformation the relationship became flatter, consistent with reduced leverage of a few high-end values and a more stable fit where data were

sparse. Overall, the patterns revealed in the plots aligned with the model comparison: a transformed niche-unfilling model was considerably more supported than the raw one, with a ΔAICc of −23.642.

1.5 Transferability of native species distribution models

We employed alien presences as an independent evaluation set to test the transferability of Native-SDMs within recipient zoo-regions. We computed the CBI (Jiménez & Soberón, 2020), using the "ecospat.boyce" function from the ecospat R package, and we derived both the AUC, and the maximized TSS via the "evalAUC" and "evalTSS" functions in the enmSdmX R package (Smith, 2024). For AUC and TSS, pseudo-absences (pseudo-absences) were generated with the "randomPoints" function from the dismo R package (Hijmans, Phillips, et al., 2024). We sampled pseudo-absences as follows: (1) matching the number of alien presences within the alien background; (2) if unoccupied cells were insufficient to reach this target, matching the 5% of the background (Rausell-Moreno et al., 2025); and (3) if still inadequate, matching the number of alien presences across all realms (Biancolini & Rondinini, 2025). Finally, we calculated Sensitivity and Specificity at the threshold that maximizes TSS using a custom version of evalTSS.

1.5.1 Transformation of transferability metrics

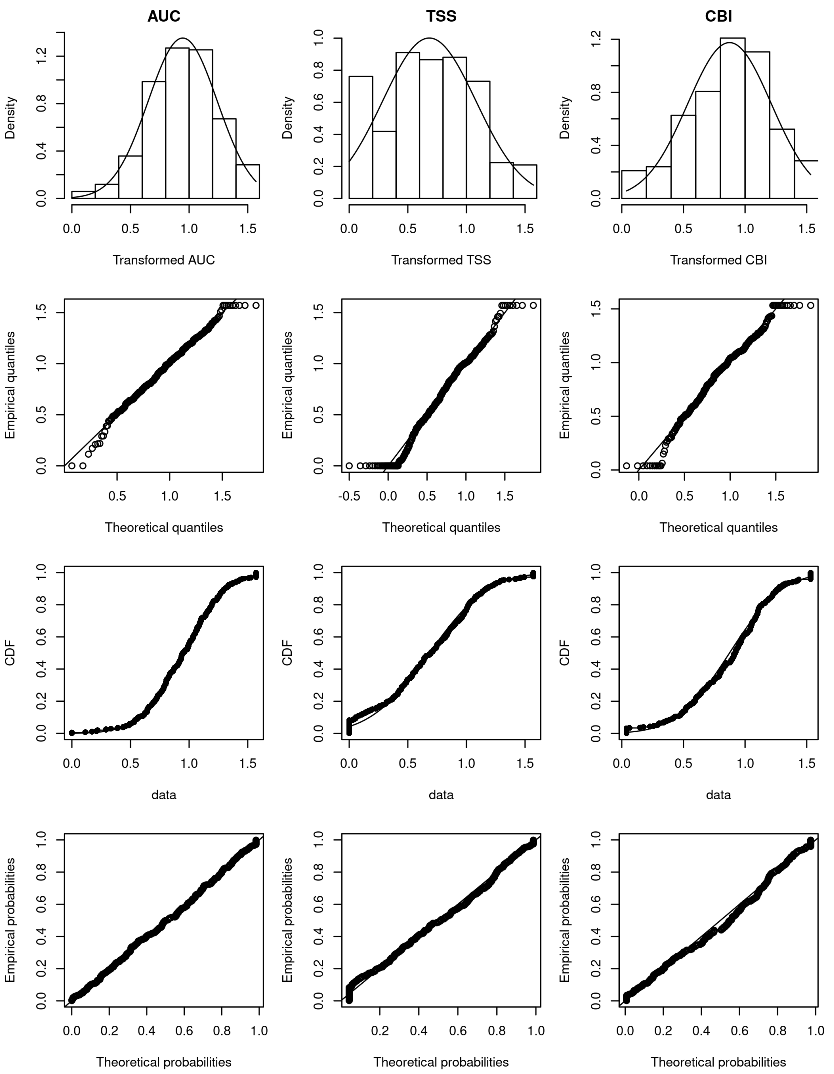

Figure S1.16: Goodness-of-fit diagnostics for the beta distribution fitted to the transformed AUC, TSS and CBI values. The plots show: (first-row) empirical histogram and theoretical beta density (red line), (second-row) Q-Q plot comparing empirical and theoretical quantiles, (third-row) empirical and theoretical cumulative distribution functions and (forth-row) P-P plot comparing empirical and theoretical probabilities.

The Area Under the receiver operating characteristic Curve (AUC) varied from 0 to 1 with a median of 0.660 (± 0.241); slight negative skewness of -0.487 showed more high-performing models than low ones, and a kurtosis of 2.51 suggested mildly lighter tails. True Skill Statistic (TSS) ranged between 0 and 1 with a median of 0.411 (± 0.306); its skewness of 0.168 indicated an almost symmetric distribution with a slight tilt toward higher values, and kurtosis of 1.81 implied a flatter profile with lighter tails. The Continuous Boyce Index (CBI) spanned -1 to 1 with a median of 0.267 (± 0.553); skewness of -0.415 signaled a modest concentration of higher-quality models, and kurtosis of 2.16 reflected a marginally platykurtic shape. Sensitivity ranged from 0.3 to 100 with a median of 98.1 (± 19.28); its marked negative skewness of -2.055 showed that most models achieved very high Sensitivity but a few performed poorly, while kurtosis of 7.12 pointed to a heavy-tailed distribution. Specificity lay between 0 and 100 with a median of 62.8 (± 32.86); skewness of -0.377 indicated a slight lean toward

higher Specificity values, and kurtosis of 1.78 suggested a broader peak, and thinner tails than a normal curve.

For both the AUC and the TSS we applied an arcsine square root transformation using the formula asin sqrt (y). This classical transformation for proportional data makes variance more constant and residuals more normally distributed, thereby meeting the assumptions of a Gaussian model (Zuur et al., 2007, 2009). The Boyce continuous index (CBI), which originally ranges from - 1 to 1, was first rescaled to span 0 to 1 by computing (CBI + 1) / 2 and then subjected to the same arcsine square root transformation. After transforming all three metrics, we fitted normal distributions to each one by maximum likelihood estimation with the fitdist function in the fitdistrplus package. Diagnostic checks showed excellent agreement between the transformed values and their fitted normal distributions for every metric (Figure S1.16). On this basis, we proceeded to model the transformed metrics using LMMs. We treated Sensitivity and Specificity differently by converting each into a binary outcome. Using data-driven thresholds we classified sensitivity and specificity as "high" when they exceeded their first quartile. We then fitted a binomial GLMM with a logit link to these binary variables.

1.6 GLMM diagnostics

We evaluated GLMM fit and assumptions using simulation-based residual diagnostics in DHARMa (Hartig, 2022; Hartig et al., 2024). For each fitted glmmTMB model, we generated randomized quantile residuals with simulateResiduals, which simulates responses under the fitted model and compares them to observations to produce scaled residuals expected to be uniform on [0,1] under correct specification. We inspected DHARMa diagnostic plots (Q-Q of scaled residuals and residuals versus fitted values) and tested residual uniformity (Kolmogorov-Smirnov), dispersion, and extreme residuals (outlier test) (Hartig, 2022; Hartig et al., 2024). Diagnostics were run for the full models and for best-supported models (ΔAICc < 4) using a fixed number of simulations (n = 1000). When the outlier test was significant, we identified extreme observations based on the simulation threshold, inspected their response and fitted mean, and refit the same model after excluding those rows to assess sensitivity by comparing fixed-effect estimates. This allowed us to assess whether outlier signals were driven by a few extreme observations with limited effects on fixed-effect estimates and model support, or instead indicated broader violations of model assumptions.

1.7 Graphic visualization

We created visual plots, including histograms, boxplots, and bar charts, primarily using the ggplot2 R package (Lüdecke et al., 2025). We employed the viridis R package (Garnier et al., 2024) for its visually appealing and colorblind-friendly color gradients, and the scales R package (Wickham et al., 2023) to ensure effective axis scaling. We then utilized gridExtra to arrange these multiple plots into single, cohesive layouts. As a preparatory step, we used the reshape2 R package (Wickham, 2020) to transform the data into formats suitable for visualization. We generated all marginal-effect and response plots using ggplot2 together with the ggeffects R package (Lüdecke et al., 2025).

1.8 References

Biancolini, D., Pacifici, M., Falaschi, M., Bellard, C., Blackburn, T. M., Ficetola, G. F., & Rondinini, C. (2024). Global distribution of alien mammals under climate change. *Global Change Biology*. https://doi.org/10.1111/gcb.17560

Biancolini, D., & Rondinini, C. (2025). Global enhancers and constraints of alien range size in mammals: The roles of species attributes, invasion history, and ecological contexts. *Global Ecology and Biogeography*, *34*(7), e70081. https://doi.org/10.1111/geb.70081

Biancolini, D., Vascellari, V., Melone, B., Blackburn, T. M., Cassey, P., Scrivens, S. L., & Rondinini, C. (2021). DAMA: The global Distribution of Alien Mammals database. *Ecology*, *102*(11), e03474. https://doi.org/10.1002/ecy.3474

Broennimann, O., Cola, V. D., Petitpierre, B., Breiner, F., Scherrer, D., D`Amen, M., Randin, C., Engler, R., Hordijk, W., Mod, H., Pottier, J., Febbraro, M. D., Pellissier, L., Pio, D., Mateo, R. G., Dubuis, A., Maiorano, L., Psomas, A., Ndiribe, C., … Guisan, A. (2024). *ecospat: Spatial Ecology Miscellaneous Methods* (Version 4.1.1). https://cran.r-project.org/web/packages/ecospat/index.html

Delignette-Muller, M.-L., Dutang, C., Pouillot, R., Denis, J.-B., & Siberchicot, A. (2024). *fitdistrplus: Help to Fit of a Parametric Distribution to Non-Censored or Censored Data* (Version 1.2-1). The Comprehensive R Archive Network. https://cran.r-project.org/web/packages/fitdistrplus/index.html

Di Cola, V., Broennimann, O., Petitpierre, B., Breiner, F. T., D'Amen, M., Randin, C., Engler, R., Pottier, J., Pio, D., Dubuis, A., Pellissier, L., Mateo, R. G., Hordijk, W., Salamin, N., & Guisan, A. (2017). ecospat: An R package to support spatial analyses and modeling of species niches and distributions. *Ecography*, *40*(6), 774–787. https://doi.org/10.1111/ecog.02671

Faurby, S., Davis, M., Pedersen, R. Ø., Schowanek, S. D., Antonelli1, A., & Svenning, J. (2018). PHYLACINE 1.2: The Phylogenetic Atlas of Mammal Macroecology. *Ecology*, *99*(11), 2626–2626. https://doi.org/10.1002/ecy.2443

Garnier, S., Ross, N., Rudis, B., Sciaini, M., Camargo, A. P., & Scherer, C. (2024). *viridis: Colorblind-Friendly Color Maps for R* (Version 0.6.5). The Comprehensive R Archive Network. https://cran.r-project.org/web/packages/viridis/index.html

Hartig, F. (2022, October 12). *DHARMa: Residual diagnostics for hierarchical (multi-level/mixed) regression models*. The Comprehensive R Archive Network. https://cran.r-project.org/web/packages/DHARMa/vignettes/DHARMa.html

Hartig, F., Lohse, L., & leite, M. de S. (2024). *DHARMa: Residual Diagnostics for Hierarchical (Multi-Level / Mixed) Regression Models* (Version 0.4.7). https://cran.r-project.org/web/packages/DHARMa/index.html

Hijmans, R. J., Bivand, R., Cordano, E., Dyba, K., Pebesma, E., & Sumner, M. D. (2024). *terra: Spatial Data Analysis* (Version 1.7-83). https://cran.r-project.org/web/packages/terra/index.html

Hijmans, R. J., Phillips, S., Leathwick, J., & Elith, J. (2024). *dismo: Species Distribution Modeling* (Version 1.3-16). The Comprehensive R Archive Network. https://cran.r-project.org/web/packages/dismo/index.html

IUCN. (2020). *The IUCN Red List of Threatened Species. Version 2020-1*. IUCN Red List of Threatened Species. https://www.iucnredlist.org/en

Jiménez, L., & Soberón, J. (2020). Leaving the area under the receiving operating characteristic curve behind: An evaluation method for species distribution modelling applications based on presence-only data. *Methods in Ecology and Evolution*, *11*(12), 1571–1586. https://doi.org/10.1111/2041-210X.13479

Karger, D. N., Conrad, O., Böhner, J., Kawohl, T., Kreft, H., Soria-Auza, R. W., Zimmermann, N. E., Linder, H. P., & Kessler, M. (2017). Climatologies at high resolution for the

earth's land surface areas. *Scientific Data*, *4*(1), 170122. https://doi.org/10.1038/sdata.2017.122

Lüdecke, D., Aust, F., Crawley, S., Ben-Shachar, M. S., & Anderson, S. C. (2025). *ggeffects: Create Tidy Data Frames of Marginal Effects for "ggplot" from Model Outputs* (Version 2.3.0). The Comprehensive R Archive Network. https://cran.r-project.org/web/packages/ggeffects/index.html

Mesgaran, M. B., Cousens, R. D., & Webber, B. L. (2014). Here be dragons: A tool for quantifying novelty due to covariate range and correlation change when projecting species distribution models. *Diversity and Distributions*, *20*(10), 1147–1159. https://doi.org/10.1111/ddi.12209

Mucherino, A., Papajorgji, P. J., & Pardalos, P. M. (2009). Clustering by k-means. In A. Mucherino, P. J. Papajorgji, & P. M. Pardalos, *Data Mining in Agriculture* (Vol. 34, pp. 47–82). Springer New York. https://doi.org/10.1007/978-0-387-88615-2_3

Münkemüller, T., Lavergne, S., Bzeznik, B., Dray, S., Jombart, T., Schiffers, K., & Thuiller, W. (2012). How to measure and test phylogenetic signal. *Methods in Ecology and Evolution*, *3*(4), 743–756. https://doi.org/10.1111/j.2041-210X.2012.00196.x

Paradis, E., Blomberg, S., Bolker, B., Brown, J., Claramunt, S., Claude, J., Cuong, H. S., Desper, R., Didier, G., Durand, B., Dutheil, J., Ewing, R. J., Gascuel, O., Guillerme, T., Heibl, C., Ives, A., Jones, B., Krah, F., Lawson, D., … Vienne, D. de. (2024). *ape: Analyses of Phylogenetics and Evolution* (Version 5.8). The Comprehensive R Archive Network. https://cran.r-project.org/web/packages/ape/index.html

Pebesma, E., Bivand, R., Racine, E., Sumner, M., Cook, I., Keitt, T., Lovelace, R., Wickham, H., Ooms, J., Müller, K., Pedersen, T. L., Baston, D., & Dunnington, D. (2023). *sf: Simple Features for R* (Version 1.0-15). The Comprehensive R Archive Network. https://cran.r-project.org/web/packages/sf/index.html

Peterson, R. A. (2023a). *bestNormalize: Normalizing Transformation Functions* (Version 1.9.1). The Comprehensive R Archive Network. https://cran.r-project.org/web/packages/bestNormalize/index.html

Peterson, R. A. (2023b, August 18). *Using the bestNormalize Package*. The Comprehensive R Archive Network. https://cran.r-project.org/web/packages/bestNormalize/vignettes/bestNormalize.html

Rausell-Moreno, A., Galiana, N., Naimi, B., & Araújo, M. B. (2025). Improving species distribution models by optimising background points: Impacts on current and future climate projections. *Ecological Modelling*, *507*, 111177. https://doi.org/10.1016/j.ecolmodel.2025.111177

Revell, L. J. (2024). *phytools: Phylogenetic Tools for Comparative Biology (and Other Things)* (Version 2.3-0). https://cran.r-project.org/web/packages/phytools/index.html

Schliep, K., Paradis, E., Martins, L. de O., Potts, A., Bardel-Kahr, I., White, T. W., Stachniss, C., Kendall, M., Halabi, K., Bilderbeek, R., Winchell, K., Revell, L., Gilchrist, M., Beaulieu, J., O'Meara, B., Qu, L., Brown, J., & Claramunt, S. (2024). *phangorn: Phylogenetic Reconstruction and Analysis* (Version 2.12.1). https://cran.r-project.org/web/packages/phangorn/index.html

Seebens, H., Blackburn, T. M., Dyer, E. E., Genovesi, P., Hulme, P. E., Jeschke, J. M., Pagad, S., Pyšek, P., Winter, M., Arianoutsou, M., Bacher, S., Blasius, B., Brundu, G., Capinha, C., Celesti-Grapow, L., Dawson, W., Dullinger, S., Fuentes, N., Jäger, H., … Essl, F. (2017). No saturation in the accumulation of alien species worldwide. *Nature Communications*, *8*, 14435. https://doi.org/10.1038/ncomms14435

Smith, A. B. (2024). *enmSdmX: Species Distribution Modeling and Ecological Niche Modeling* (Version 1.1.9). The Comprehensive R Archive Network. https://cran.r-project.org/web/packages/enmSdmX/index.html

Smithson, M., & Verkuilen, J. (2006). A better lemon squeezer? Maximum-likelihood regression with beta-distributed dependent variables. *Psychological Methods*, *11*(1), 54–71. https://doi.org/10.1037/1082-989X.11.1.54

Wickham, H. (2020). *reshape2: Flexibly Reshape Data: A Reboot of the Reshape Package* (Version 1.4.4). The Comprehensive R Archive Network. https://cran.r-project.org/web/packages/reshape2/index.html

Wickham, H., Chang, W., Henry, L., Pedersen, T. L., Takahashi, K., Wilke, C., Woo, K., Yutani, H., Dunnington, D., Brand, T. van den, Posit, & PBC. (2024). *ggplot2: Create Elegant Data Visualisations Using the Grammar of Graphics* (Version 3.5.1) [R]. The Comprehensive R Archive Network. https://cran.r-project.org/web/packages/ggplot2/index.html

Wickham, H., Pedersen, T. L., Seidel, D., Posit, & PBC. (2023). *scales: Scale Functions for Visualization* (Version 1.3.0). The Comprehensive R Archive Network. https://cran.r-project.org/web/packages/scales/index.html

Zuur, A. F., Ieno, E. N., & Smith, G. M. (2007). *Analysing Ecological Data*. Springer New York. https://link.springer.com/book/10.1007/978-0-387-45972-1

Zuur, A. F., Ieno, E. N., Walker, N., Saveliev, A. A., & Smith, G. M. (2009). *Mixed effects models and extensions in ecology with R*. Springer New York. https://doi.org/10.1007/978-0-387-87458-6
