# Supplementary material for: Global Patterns of Niche Changes in Alien Mammals: Potential Drivers and Significance for Invasion Projections
Source: Glob Chang Biol. 2026 Mar 20;32(3):e70755. doi: 10.1111/gcb.70755 (PMC13004023; doi:10.1111/gcb.70755)
Supplement: Supplementary file 2 — Appendix S2: Supporting Results. [file GCB-32-e70755-s001.pdf]

Global Change Biology

Appendix S1 for:

Global patterns of niche changes in alien mammals: potential drivers and significance for invasion projections

Table of Contents

Supporting Results	Page 1
Table S2.1: Niche expansion, stability and unfilling for each species in each realm.	Page 1
Table S2.2: Area Under the receiver operating characteristic Curve (AUC), maximized True Skill Statistic (TSS), Continuous Boyce Index (CBI), Sensitivity (Sens) and Specificity (Spec) of Native-SDMs for each species in each realm. Pseudo-absences (PAs) used sampling methods: Equal to the number of presences, 5% of the alien background and all Realms.	Page 7
Figure S2.1: Area Under the receiver operating characteristic Curve (AUC), maximized True Skill Statistic (TSS), Continuous Boyce Index (CBI), Sensitivity (Sens) and Specificity (Spec) of Native-SDMs for each species in each realm.	Page 14
Figure S2.2: DHARMa tests and diagnostic plots for the global niche-expansion GLMM.	Page 15
Figure S2.3: DHARMa tests and diagnostic plots for the best-supported niche-expansion GLMM number 1369.	Page 15
Figure S2.4: DHARMa tests and diagnostic plots for the best-supported niche-expansion GLMM number 1400.	Page 15
Figure S2.5: DHARMa tests and diagnostic plots for the best-supported niche-expansion GLMM number 1880.	Page 15
Figure S2.6: DHARMa tests and diagnostic plots for the best-supported niche-expansion GLMM number 1368.	Page 16
Figure S2.7: DHARMa tests and diagnostic plots for the best-supported niche-expansion GLMM number 345.	Page 16
Figure S2.8: DHARMa tests and diagnostic plots for the best-supported niche-expansion GLMM number 344.	Page 16
Figure S2.9: DHARMa tests and diagnostic plots for the global niche-unfilling GLMM.	Page 17
Figure S2.10: DHARMa tests and diagnostic plots for the best-supported niche-unfilling GLMM number 697.	Page 17
Figure S2.11: DHARMa tests and diagnostic plots for the best-supported niche-unfilling GLMM number 569.	Page 17
Figure S2.12: DHARMa tests and diagnostic plots for the best-supported niche-unfilling GLMM number 696.	Page 17
Figure S2.13: DHARMa diagnostics tests and plots for the LMM of the Area Under the receiver operating characteristic Curve.	Page 18
Figure S2.14: DHARMa diagnostics tests and plots for the LMM of the maximized True Skill Statistic.	Page 18
Figure S2.15: DHARMa diagnostics tests and plots for the GLMM of Sensitivity.	Page 18
Figure S2.16: DHARMa diagnostics tests and plots for the GLMM of Specificity.	Page 18

Global Change Biology

Figure S2.17: DHARMa diagnostics tests and plots for the LMM of the Continuous Boyce Index.	Page 18
Table S2.3: Comparison of niche-expansion models, ranked by Akaike's Information Criterion corrected for small sample sizes (AICc). Only models with a ΔAICc < 7 are reported, along with their corresponding Bayesian Information Criterion (BIC), log-likelihood (logLik), and residual degrees of freedom (RDF).	Page 19
Table S2.4: Comparison of niche-unfilling models, ranked by Akaike's Information Criterion corrected for small sample sizes (AICc). Only models with a ΔAICc < 7 are reported, along with their corresponding Bayesian Information Criterion (BIC), log-likelihood (logLik), and residual degrees of freedom (RDF).	Page 19
Table S2.5: Fixed Variable name, Estimate, Standard Error (Std. Error), 95% Confidence Interval (CI), z-value, p-value, and Significance levels (Sign) of the niche-expansion models with a ΔAICc < 7.	Page 19
Table S2.6: Fixed Variable name, Estimate, Standard Error (Std. Error), 95% Confidence Interval (CI), z-value, p-value, and Significance levels of the niche-unfilling models with a ΔAICc < 7.	Page 20
Table S2.7: Random-effect variance and standard deviation of the niche-expansion models with a ΔAICc < 7.	Page 21
Table S2.8: Random-effect variance and standard deviation of the niche-unfilling models with a ΔAICc < 7.	Page 21
Table S2.9: Fixed Variable name, Estimate, adjusted Standard Error (Std. Error) and 95% Confidence Interval (CI) of the full-averaged niche-expansion model.	Page 22
Figure S2.18: Marginal effects of six variables on the probability of niche expansion from the full-averaged GLMM. Each panel shows the predicted probability (solid line) and its 95% confidence ribbon as a function of one standardized predictor while holding all others at their mean.	Page 22
Table S2.10: Fixed Variable name, Estimate, adjusted Standard Error (Std. Error) and 95% Confidence Interval (CI) of the full-averaged niche-unfilling model.	Page 23
Figure S2.19: Marginal effects of six variables on the probability of niche unfilling from the full-averaged GLMM. Each panel shows the predicted probability (solid line) and its 95% confidence ribbon as a function of one standardized predictor while holding all others at their mean.	Page 23
Table S2.11: Fixed Variable name, Estimate, Standard Error (Std. Error), 95% Confidence Interval (CI), z-value, p-value, and Significance levels of the Native-SDMs transferability models.	Page 24
Table S2.12: Random effects Standard Deviation and Variance for the Native-SDMs transferability models.	Page 24
Table S2.13: The Akaike's Information Criterion corrected for small sample sizes (AICc), Bayesian Information Criterion (BIC), log-likelihood (logLik), residual standard deviation (sigma) and residual degrees of freedom (RDF) for the Native-SDMs transferability models.	Page 24
Figure S2.20: Marginal effects of six variables on the probability of Native-SDMs AUC, TSS, CBI, Sensitivity and Specificity from (G)LMMs. Each panel shows the predicted probability (solid line) and its 95% confidence ribbon as a function of one standardized predictor while holding all others at their mean.	Page 25

Global Change Biology

2. Supporting Results

In this appendix we provide niche expansion, stability and unfilling values and Native-SDMs Area Under the receiver operating characteristic Curve (AUC), maximized True Skill Statistic (TSS), Continuous Boyce Index (CBI), Sensitivity and Specificity for all species in their realm. Furthermore, we present DHARMa tests and diagnostic plots for Generalized Linear Mixed Models (GLMMs) of niche metrics and Native-SDMs transferability, along with tables of niche niche metrics, performance metrics, model selection, and variable effects.

For one the best-supported niche-unfilling models (ID 697, Figure S2.10), DHARMa indicated adequate residual uniformity (KS p = 0.294) and dispersion (p = 0.634), but the outlier test was significant (p = 0.031) due to three extreme observations. Excluding them did not change the sign of any coefficient and produced only minor effect-size shifts (0.069 for native mammal richness; 0.046 for human disturbance; 0.043 for Introduction effort). These results indicate that inference is robust to extreme cases and support the use of model 697 in multi-model inference.

Table S2.1: Niche expansion, stability and unfilling for each species in each realm.

Realm	Species	Expansion	Stability	Unfilling
Afrotropical	*Beatragus hunteri*	0.825	0.175	0.989
Afrotropical	*Cercopithecus mona*	0.000	1.000	0.834
Afrotropical	*Chlorocebus sabaeus*	0.179	0.821	0.998
Afrotropical	*Civettictis civetta*	0.001	0.999	0.885
Afrotropical	*Connochaetes taurinus*	0.000	1.000	0.564
Afrotropical	*Dama dama*	0.983	0.017	0.711
Afrotropical	*Hemitragus jemlahicus*	0.000	1.000	0.923
Afrotropical	*Herpestes auropunctatus*	0.000	1.000	0.999
Afrotropical	*Mustela nivalis*	0.885	0.115	0.995
Afrotropical	*Myocastor coypus*	0.000	1.000	0.457
Afrotropical	*Nanger soemmerringii*	0.000	1.000	0.942
Afrotropical	*Rattus tanezumi*	0.000	1.000	0.825
Afrotropical	*Rusa unicolor*	0.000	1.000	0.963
Afrotropical	*Sciurus carolinensis*	0.119	0.881	0.400
Afrotropical	*Suncus murinus*	0.018	0.982	0.350
Afrotropical	*Tragelaphus angasii*	0.974	0.026	0.950
Afrotropical	*Viverricula indica*	0.000	1.000	0.873
Australian	*Axis axis*	0.077	0.923	0.175
Australian	*Axis porcinus*	0.063	0.937	0.903
Australian	*Bettongia lesueur*	0.000	1.000	0.899
Australian	*Bettongia penicillata*	0.201	0.799	0.596
Australian	*Bos javanicus*	0.000	1.000	0.888
Australian	*Bubalus bubalis*	0.079	0.921	0.566
Australian	*Camelus dromedarius*	0.000	1.000	0.521
Australian	*Cervus canadensis*	0.648	0.352	0.888
Australian	*Cervus elaphus*	0.048	0.952	0.000
Australian	*Cervus nippon*	0.000	1.000	0.618
Australian	*Dama dama*	0.743	0.257	0.143
Australian	*Erinaceus europaeus*	0.066	0.934	0.002
Australian	*Hemitragus jemlahicus*	0.276	0.724	0.024

Australian	*Isoodon obesulus*	0.000	1.000	0.950
Australian	*Lepus europaeus*	0.138	0.862	0.000
Australian	*Macropus agilis*	0.000	1.000	0.970
Australian	*Macropus eugenii*	0.947	0.053	0.682
Australian	*Macropus giganteus*	0.000	1.000	0.993
Australian	*Macropus rufogriseus*	0.151	0.849	0.760
Australian	*Mustela erminea*	0.001	0.999	0.005
Australian	*Mustela nivalis*	0.043	0.957	0.073
Australian	*Mustela putorius*	0.075	0.925	0.001
Australian	*Odocoileus virginianus*	0.150	0.850	0.759
Australian	*Ornithorhynchus anatinus*	0.000	1.000	0.953
Australian	*Oryctolagus cuniculus*	0.286	0.714	0.004
Australian	*Petaurus breviceps*	0.169	0.831	0.893
Australian	*Petrogale lateralis*	0.903	0.097	0.999
Australian	*Phascolarctos cinereus*	0.000	1.000	0.553
Australian	*Pseudocheirus peregrinus*	0.000	1.000	0.891
Australian	*Rattus exulans*	0.717	0.283	0.131
Australian	*Rupicapra rupicapra*	0.281	0.719	0.000
Australian	*Rusa timorensis*	0.969	0.031	0.012
Australian	*Rusa unicolor*	0.000	1.000	0.765
Australian	*Sarcophilus harrisii*	0.000	1.000	0.940
Australian	*Tachyglossus aculeatus*	0.000	1.000	0.998
Australian	*Thylogale billardierii*	0.000	1.000	0.616
Australian	*Trichosurus vulpecula*	0.180	0.820	0.694
Australian	*Vombatus ursinus*	0.000	1.000	0.958
Australian	*Vulpes vulpes*	0.000	1.000	0.333
Madagascan	*Eulemur fulvus*	0.000	1.000	0.997
Madagascan	*Eulemur mongoz*	1.000	0.000	1.000
Madagascan	*Herpestes auropunctatus*	0.000	1.000	0.957
Madagascan	*Potamochoerus larvatus*	0.013	0.987	0.064
Madagascan	*Suncus etruscus*	0.001	0.999	0.106
Madagascan	*Suncus murinus*	0.000	1.000	0.076
Madagascan	*Tenrec ecaudatus*	0.000	1.000	0.873
Madagascan	*Viverricula indica*	0.000	1.000	0.113
Nearctic	*Alces alces*	0.000	1.000	0.936
Nearctic	*Ammotragus lervia*	0.003	0.997	0.638
Nearctic	*Antilope cervicapra*	1.000	0.000	1.000
Nearctic	*Axis axis*	0.791	0.209	0.914
Nearctic	*Bison bison*	0.312	0.688	0.994
Nearctic	*Boselaphus tragocamelus*	0.006	0.994	0.962
Nearctic	*Castor canadensis*	0.000	1.000	0.669
Nearctic	*Cervus canadensis*	0.158	0.842	0.988
Nearctic	*Cervus elaphus*	0.003	0.997	0.820
Nearctic	*Cervus nippon*	0.203	0.797	0.695
Nearctic	*Dama dama*	0.888	0.112	0.590
Nearctic	*Dasypus novemcinctus*	0.000	1.000	0.712
Nearctic	*Didelphis virginiana*	0.074	0.926	0.091
Nearctic	*Herpestes auropunctatus*	0.002	0.998	0.658
Nearctic	*Lepus americanus*	0.000	1.000	0.778
Nearctic	*Lepus arcticus*	0.000	1.000	0.989
Nearctic	*Lepus californicus*	0.000	1.000	0.799
Nearctic	*Lepus europaeus*	0.000	1.000	0.448
Nearctic	*Martes americana*	0.001	0.999	0.524
Nearctic	*Martes foina*	0.000	1.000	0.953
Nearctic	*Microtus californicus*	0.000	1.000	0.980
Nearctic	*Myocastor coypus*	0.000	1.000	0.068

Nearctic	*Myodes gapperi*	0.000	1.000	0.877
Nearctic	*Neovison vison*	0.000	1.000	0.914
Nearctic	*Odocoileus hemionus*	0.135	0.865	0.099
Nearctic	*Odocoileus virginianus*	0.000	1.000	0.952
Nearctic	*Ondatra zibethicus*	0.001	0.999	0.624
Nearctic	*Oreamnos americanus*	0.006	0.994	0.243
Nearctic	*Oryctolagus cuniculus*	0.050	0.950	0.769
Nearctic	*Oryx gazella*	0.339	0.661	0.874
Nearctic	*Ovibos moschatus*	0.000	1.000	0.554
Nearctic	*Ovis canadensis*	0.000	1.000	0.229
Nearctic	*Ovis orientalis*	0.881	0.119	0.993
Nearctic	*Peromyscus maniculatus*	0.000	1.000	0.767
Nearctic	*Petrogale penicillata*	0.000	0.000	1.000
Nearctic	*Procyon lotor*	0.006	0.994	0.854
Nearctic	*Rangifer tarandus*	0.003	0.997	0.934
Nearctic	*Rattus exulans*	0.111	0.889	0.118
Nearctic	*Rusa unicolor*	0.000	1.000	0.766
Nearctic	*Sciurus aberti*	0.007	0.993	0.306
Nearctic	*Sciurus carolinensis*	0.149	0.851	0.095
Nearctic	*Sciurus niger*	0.032	0.968	0.064
Nearctic	*Sorex cinereus*	0.000	1.000	0.895
Nearctic	*Sylvilagus floridanus*	0.039	0.961	0.178
Nearctic	*Tamias striatus*	0.000	1.000	0.949
Nearctic	*Tamiasciurus hudsonicus*	0.001	0.999	0.383
Nearctic	*Urocitellus parryii*	0.143	0.857	0.947
Nearctic	*Vulpes lagopus*	0.000	1.000	0.956
Nearctic	*Vulpes vulpes*	0.000	1.000	0.436
Neotropical	*Antilope cervicapra*	1.000	0.000	1.000
Neotropical	*Axis axis*	0.504	0.496	0.983
Neotropical	*Bubalus bubalis*	0.314	0.686	0.124
Neotropical	*Callithrix geoffroyi*	0.119	0.881	0.288
Neotropical	*Callithrix jacchus*	0.319	0.681	0.366
Neotropical	*Callithrix penicillata*	0.116	0.884	0.233
Neotropical	*Callosciurus erythraeus*	0.000	1.000	0.834
Neotropical	*Castor canadensis*	0.000	1.000	0.662
Neotropical	*Cercopithecus mona*	0.000	1.000	0.947
Neotropical	*Cervus elaphus*	0.013	0.987	0.021
Neotropical	*Chaetophractus villosus*	0.000	1.000	0.991
Neotropical	*Dama dama*	0.621	0.379	6.060e-05
Neotropical	*Dasyprocta leporina*	0.000	1.000	0.461
Neotropical	*Dasypus novemcinctus*	0.000	1.000	0.908
Neotropical	*Didelphis marsupialis*	0.000	1.000	0.641
Neotropical	*Herpestes auropunctatus*	0.000	1.000	0.981
Neotropical	*Hippopotamus amphibius*	0.100	0.900	0.908
Neotropical	*Lepus europaeus*	0.115	0.885	1.631e-05
Neotropical	*Lycalopex griseus*	0.002	0.998	0.829
Neotropical	*Neovison vison*	0.017	0.983	0.073
Neotropical	*Odocoileus virginianus*	0.000	0.000	0.000
Neotropical	*Ondatra zibethicus*	0.000	1.000	0.524
Neotropical	*Oryctolagus cuniculus*	0.166	0.834	2.448e-05
Neotropical	*Rangifer tarandus*	0.122	0.878	0.969
Neotropical	*Saguinus oedipus*	0.000	1.000	0.834
Neotropical	*Saimiri sciureus*	0.105	0.895	0.507
Neotropical	*Sciurus stramineus*	0.000	1.000	0.983
Neotropical	*Vicugna vicugna*	0.000	1.000	0.618
Oceanian	*Babyrousa babyrussa*	0.610	0.390	0.000

Oceanian	*Bubalus bubalis*	0.897	0.103	0.187
Oceanian	*Crocidura maxi*	0.000	1.000	0.945
Oceanian	*Crocidura monticola*	0.000	1.000	0.304
Oceanian	*Dendrolagus matschiei*	0.102	0.898	0.956
Oceanian	*Herpestes auropunctatus*	0.000	1.000	0.942
Oceanian	*Herpestes fuscus*	0.000	1.000	0.721
Oceanian	*Lepus nigricollis*	0.035	0.965	0.801
Oceanian	*Macaca fascicularis*	0.000	1.000	0.439
Oceanian	*Macaca nigra*	0.062	0.938	0.840
Oceanian	*Macropus agilis*	0.005	0.995	0.918
Oceanian	*Oryctolagus cuniculus*	0.750	0.250	0.723
Oceanian	*Paradoxurus hermaphroditus*	0.000	1.000	0.610
Oceanian	*Petaurus breviceps*	0.000	1.000	0.481
Oceanian	*Phalanger orientalis*	0.000	1.000	0.190
Oceanian	*Rangifer tarandus*	0.190	0.810	0.001
Oceanian	*Rattus argentiventer*	0.000	1.000	0.868
Oceanian	*Rattus exulans*	0.016	0.984	0.197
Oceanian	*Rattus nitidus*	0.049	0.951	0.963
Oceanian	*Rattus praetor*	0.009	0.991	0.076
Oceanian	*Rattus tanezumi*	0.088	0.912	0.612
Oceanian	*Rusa marianna*	0.099	0.901	0.598
Oceanian	*Rusa timorensis*	0.389	0.611	0.006
Oceanian	*Spilocuscus maculatus*	0.000	1.000	0.245
Oceanian	*Suncus murinus*	0.000	1.000	0.540
Oceanian	*Sus celebensis*	0.000	1.000	0.262
Oceanian	*Tenrec ecaudatus*	0.069	0.931	0.390
Oceanian	*Thylogale browni*	0.000	1.000	0.441
Oceanian	*Thylogale brunii*	0.000	1.000	0.996
Oceanian	*Viverra tangalunga*	0.002	0.998	0.024
Oriental	*Axis axis*	0.000	1.000	0.992
Oriental	*Axis porcinus*	0.563	0.437	0.994
Oriental	*Bandicota indica*	0.003	0.997	0.652
Oriental	*Bos javanicus*	0.051	0.949	0.158
Oriental	*Bubalus bubalis*	0.227	0.773	0.322
Oriental	*Callosciurus notatus*	0.000	1.000	0.978
Oriental	*Callosciurus prevostii*	0.002	0.998	0.225
Oriental	*Cervus nippon*	0.000	1.000	0.987
Oriental	*Elephas maximus*	0.000	1.000	0.724
Oriental	*Funambulus pennantii*	0.000	1.000	0.996
Oriental	*Herpestes auropunctatus*	0.000	1.000	0.966
Oriental	*Hystrix javanica*	0.003	0.997	0.476
Oriental	*Lepus nigricollis*	0.000	1.000	0.975
Oriental	*Macaca fascicularis*	0.000	1.000	0.428
Oriental	*Macaca leonina*	0.000	1.000	0.986
Oriental	*Macaca nemestrina*	0.000	1.000	0.521
Oriental	*Manis culionensis*	0.638	0.362	0.920
Oriental	*Muntiacus muntjak*	0.007	0.993	0.822
Oriental	*Mus caroli*	0.000	1.000	0.821
Oriental	*Mus terricolor*	0.018	0.982	0.993
Oriental	*Mustela itatsi*	0.024	0.976	0.895
Oriental	*Paradoxurus hermaphroditus*	0.004	0.996	0.189
Oriental	*Phalanger orientalis*	0.508	0.492	0.988
Oriental	*Rattus argentiventer*	0.006	0.994	0.013
Oriental	*Rattus exulans*	0.286	0.714	0.618
Oriental	*Rattus nitidus*	0.154	0.846	0.764
Oriental	*Rattus tanezumi*	0.053	0.947	0.290

Oriental	*Rusa timorensis*	0.260	0.740	0.023
Oriental	*Semnopithecus entellus*	0.000	1.000	0.913
Oriental	*Spilocuscus maculatus*	0.000	1.000	0.990
Oriental	*Suncus murinus*	0.000	1.000	0.597
Oriental	*Sus celebensis*	0.001	0.999	0.540
Oriental	*Trachypithecus auratus*	0.001	0.999	0.303
Oriental	*Viverra tangalunga*	0.251	0.749	0.006
Oriental	*Viverra zibetha*	0.000	1.000	0.987
Oriental	*Viverricula indica*	0.000	1.000	0.843
Palearctic	*Ammotragus lervia*	0.029	0.971	0.778
Palearctic	*Apodemus sylvaticus*	0.005	0.995	0.185
Palearctic	*Atelerix algirus*	0.031	0.969	0.291
Palearctic	*Axis axis*	0.973	0.027	0.997
Palearctic	*Callosciurus erythraeus*	0.000	1.000	0.611
Palearctic	*Callosciurus finlaysonii*	1.000	0.000	1.000
Palearctic	*Capra aegagrus*	0.000	1.000	0.897
Palearctic	*Capra ibex*	0.048	0.952	0.536
Palearctic	*Capra sibirica*	0.000	1.000	0.731
Palearctic	*Capreolus capreolus*	0.000	1.000	0.970
Palearctic	*Capreolus pygargus*	0.000	1.000	0.913
Palearctic	*Castor canadensis*	0.000	1.000	0.554
Palearctic	*Castor fiber*	0.000	1.000	0.906
Palearctic	*Cervus canadensis*	0.000	1.000	0.981
Palearctic	*Cervus elaphus*	0.000	1.000	0.986
Palearctic	*Cervus nippon*	2.762e-05	1.000	0.510
Palearctic	*Crocidura dsinezumi*	0.000	1.000	0.689
Palearctic	*Crocidura pachyura*	0.051	0.949	0.475
Palearctic	*Crocidura russula*	0.006	0.994	0.911
Palearctic	*Crocidura suaveolens*	0.000	1.000	0.864
Palearctic	*Dama dama*	0.100	0.900	0.003
Palearctic	*Desmana moschata*	0.020	0.980	0.364
Palearctic	*Eliomys quercinus*	0.000	1.000	0.511
Palearctic	*Erinaceus europaeus*	0.005	0.995	0.451
Palearctic	*Erinaceus roumanicus*	0.011	0.989	0.866
Palearctic	*Eutamias sibiricus*	0.000	1.000	0.949
Palearctic	*Genetta genetta*	0.211	0.789	0.353
Palearctic	*Glis glis*	0.000	1.000	0.635
Palearctic	*Herpestes auropunctatus*	0.000	1.000	0.875
Palearctic	*Hydropotes inermis*	1.000	0.000	1.000
Palearctic	*Hystrix cristata*	0.237	0.763	0.543
Palearctic	*Lepus capensis*	0.000	1.000	0.866
Palearctic	*Lepus corsicanus*	0.000	1.000	0.355
Palearctic	*Lepus europaeus*	0.542	0.458	0.140
Palearctic	*Lepus granatensis*	0.004	0.996	0.369
Palearctic	*Lepus timidus*	0.000	1.000	0.971
Palearctic	*Macropus rufogriseus*	0.000	1.000	0.991
Palearctic	*Marmota bobak*	0.000	1.000	0.899
Palearctic	*Marmota marmota*	0.081	0.919	0.346
Palearctic	*Martes foina*	0.000	1.000	0.799
Palearctic	*Martes martes*	0.005	0.995	0.813
Palearctic	*Martes zibellina*	0.585	0.415	0.377
Palearctic	*Meles meles*	0.000	1.000	0.833
Palearctic	*Micromys minutus*	0.000	1.000	0.842
Palearctic	*Microtus arvalis*	0.000	1.000	0.994
Palearctic	*Microtus levis*	0.947	0.053	1.000
Palearctic	*Muntiacus reevesi*	0.049	0.951	0.783

Palearctic	*Mus spretus*	0.000	1.000	0.904
Palearctic	*Mustela erminea*	0.000	1.000	0.939
Palearctic	*Mustela lutreola*	1.000	0.000	1.000
Palearctic	*Mustela nivalis*	0.000	1.000	0.856
Palearctic	*Mustela putorius*	0.002	0.998	0.752
Palearctic	*Myocastor coypus*	0.000	1.000	0.316
Palearctic	*Myodes glareolus*	0.002	0.998	0.911
Palearctic	*Myodes rutilus*	0.000	1.000	0.986
Palearctic	*Nasua nasua*	0.000	1.000	0.925
Palearctic	*Neovison vison*	0.005	0.995	0.015
Palearctic	*Nyctereutes procyonoides*	0.011	0.989	0.130
Palearctic	*Odocoileus virginianus*	0.000	1.000	0.805
Palearctic	*Ondatra zibethicus*	0.028	0.972	0.032
Palearctic	*Oryctolagus cuniculus*	0.062	0.938	0.065
Palearctic	*Ovibos moschatus*	0.003	0.997	0.030
Palearctic	*Ovis orientalis*	0.018	0.982	0.514
Palearctic	*Procyon lotor*	0.001	0.999	0.289
Palearctic	*Rangifer tarandus*	0.004	0.996	0.802
Palearctic	*Rupicapra rupicapra*	0.000	1.000	0.815
Palearctic	*Sciurus anomalus*	0.000	1.000	0.990
Palearctic	*Sciurus carolinensis*	0.038	0.962	0.562
Palearctic	*Sciurus vulgaris*	0.004	0.996	0.014
Palearctic	*Suncus etruscus*	0.000	1.000	0.405
Palearctic	*Sylvilagus floridanus*	0.001	0.999	0.469
Palearctic	*Vulpes lagopus*	0.000	1.000	0.708
Palearctic	*Vulpes vulpes*	0.000	1.000	0.869
Panamanian	*Antilope cervicapra*	0.000	1.000	0.997
Panamanian	*Boselaphus tragocamelus*	0.300	0.700	0.980
Panamanian	*Bubalus bubalis*	0.000	1.000	0.941
Panamanian	*Chlorocebus sabaeus*	0.009	0.991	0.970
Panamanian	*Cuniculus paca*	0.000	1.000	0.642
Panamanian	*Dama dama*	0.000	0.000	1.000
Panamanian	*Dasyprocta leporina*	0.000	1.000	0.705
Panamanian	*Dasyprocta mexicana*	0.000	1.000	0.584
Panamanian	*Dasyprocta punctata*	0.000	1.000	0.730
Panamanian	*Dasypus novemcinctus*	0.000	1.000	0.916
Panamanian	*Didelphis marsupialis*	0.000	1.000	0.499
Panamanian	*Erythrocebus patas*	0.000	1.000	0.994
Panamanian	*Herpestes auropunctatus*	0.000	1.000	0.531
Panamanian	*Lepus europaeus*	1.000	0.000	1.000
Panamanian	*Macaca arctoides*	0.000	1.000	0.985
Panamanian	*Macaca mulatta*	0.000	1.000	0.968
Panamanian	*Odocoileus virginianus*	0.000	1.000	0.607
Panamanian	*Oryctolagus cuniculus*	0.000	0.000	1.000
Panamanian	*Ovis orientalis*	1.000	0.000	1.000
Panamanian	*Procyon lotor*	0.000	1.000	0.845
Panamanian	*Sylvilagus floridanus*	0.000	1.000	0.809
Panamanian	*Tragelaphus derbianus*	0.000	1.000	0.974
Saharo-Arabian	*Apodemus sylvaticus*	0.003	0.997	0.602
Saharo-Arabian	*Atelerix algirus*	0.000	1.000	0.980
Saharo-Arabian	*Atlantoxerus getulus*	0.000	1.000	0.973
Saharo-Arabian	*Bubalus bubalis*	1.000	0.000	1.000
Saharo-Arabian	*Crocidura pachyura*	0.000	1.000	0.967
Saharo-Arabian	*Funambulus pennantii*	0.000	1.000	0.720
Saharo-Arabian	*Lepus europaeus*	0.000	1.000	0.964
Saharo-Arabian	*Mustela nivalis*	0.027	0.973	0.801

Saharo-Arabian	*Myocastor coypus*	0.004	0.996	0.627
Saharo-Arabian	*Oryctolagus cuniculus*	0.000	1.000	0.956
Saharo-Arabian	*Ovis orientalis*	0.000	1.000	0.943
Saharo-Arabian	*Suncus etruscus*	0.000	1.000	0.647
Saharo-Arabian	*Suncus murinus*	0.022	0.978	0.597
Saharo-Arabian	*Vulpes vulpes*	0.000	1.000	0.957
Sino-Japanese	*Callosciurus erythraeus*	0.000	1.000	0.443
Sino-Japanese	*Callosciurus finlaysonii*	0.962	0.038	0.995
Sino-Japanese	*Crocidura dsinezumi*	0.000	1.000	0.821
Sino-Japanese	*Eutamias sibiricus*	0.000	1.000	0.924
Sino-Japanese	*Herpestes auropunctatus*	0.000	1.000	0.943
Sino-Japanese	*Macaca cyclopis*	0.582	0.418	0.954
Sino-Japanese	*Macaca fascicularis*	0.000	1.000	0.980
Sino-Japanese	*Macaca mulatta*	0.000	1.000	0.819
Sino-Japanese	*Martes melampus*	0.024	0.976	0.861
Sino-Japanese	*Muntiacus reevesi*	0.000	1.000	0.746
Sino-Japanese	*Mustela itatsi*	0.125	0.875	0.672
Sino-Japanese	*Mustela lutreola*	1.000	0.000	1.000
Sino-Japanese	*Mustela sibirica*	0.000	1.000	0.350
Sino-Japanese	*Myocastor coypus*	0.000	1.000	0.326
Sino-Japanese	*Neovison vison*	0.000	1.000	0.674
Sino-Japanese	*Nyctereutes procyonoides*	0.620	0.380	0.956
Sino-Japanese	*Ondatra zibethicus*	0.000	1.000	0.626
Sino-Japanese	*Oryctolagus cuniculus*	0.366	0.634	0.254
Sino-Japanese	*Paguma larvata*	0.000	1.000	0.239
Sino-Japanese	*Procyon lotor*	0.000	1.000	0.427
Sino-Japanese	*Suncus murinus*	0.000	1.000	0.803

Table S2.2: Area Under the receiver operating characteristic Curve (AUC), maximized True Skill Statistic (TSS), Continuous Boyce Index (CBI), Sensitivity (Sens) and Specificity (Spec) of Native-SDMs for each species in each realm. Pseudo-absences (PAs) used sampling methods: Equal to the number of presences, 5% of the alien background and all Realms.

**Realm**	**Species**	**AUC**	**TSS**	**CBI**	**Sens**	**Spec**	**PAs**
Afrotropical	*Beatragus hunteri*	0.903	0.720	0.929	80.000	92.000	Equal
Afrotropical	*Cercopithecus mona*	0.736	0.514	-0.374	100.000	51.400	Equal
Afrotropical	*Chlorocebus sabaeus*	0.615	0.604	0.238	100.000	60.400	Equal
Afrotropical	*Civettictis civetta*	0.730	0.459	1.000	100.000	45.900	Equal
Afrotropical	*Connochaetes taurinus*	0.760	0.412	0.644	76.200	65.000	Equal
Afrotropical	*Dama dama*	0.508	0.079	0.868	10.600	97.300	5%
Afrotropical	*Hemitragus jemlahicus*	0.962	0.921	0.367	100.000	92.100	Equal
Afrotropical	*Herpestes auropunctatus*	0.260	0.011	-0.600	100.000	1.100	Equal
Afrotropical	*Mustela nivalis*	0.695	0.389	1.000	100.000	38.900	Equal
Afrotropical	*Myocastor coypus*	0.786	0.514	0.765	55.800	95.600	Equal
Afrotropical	*Nanger soemmerringii*	1.000	1.000	1.000	100.000	100.000	Equal
Afrotropical	*Rattus tanezumi*	0.707	0.410	-0.072	97.900	43.100	Equal
Afrotropical	*Rusa unicolor*	0.359	0.356	-0.664	100.000	35.600	Equal
Afrotropical	*Sciurus carolinensis*	0.523	0.052	0.250	100.000	5.200	Equal
Afrotropical	*Suncus murinus*	0.550	0.131	0.219	83.100	30.000	Equal
Afrotropical	*Tragelaphus angasii*	0.262	0.066	0.548	99.400	7.300	Equal
Afrotropical	*Viverricula indica*	0.259	0.183	-0.783	100.000	18.300	Equal
Australian	*Axis axis*	0.679	0.353	0.246	61.000	74.200	Equal
Australian	*Axis porcinus*	0.394	0.000	-1.000	100.000	0.000	Equal
Australian	*Bettongia penicillata*	1.000	1.000	0.225	100.000	100.000	Equal

Australian	*Bos javanicus*	0.903	0.891	0.248	100.000	89.100	Equal
Australian	*Bubalus bubalis*	0.717	0.415	0.872	46.700	94.900	Equal
Australian	*Camelus dromedarius*	0.864	0.621	0.937	89.600	72.500	Equal
Australian	*Cervus canadensis*	0.827	0.771	0.475	77.800	99.300	Equal
Australian	*Cervus elaphus*	0.918	0.694	0.496	91.400	78.000	Equal
Australian	*Cervus nippon*	0.945	0.866	1.000	99.500	87.100	Equal
Australian	*Dama dama*	0.517	0.218	-0.839	94.900	26.900	5%
Australian	*Erinaceus europaeus*	0.755	0.630	-0.286	69.700	93.300	Equal
Australian	*Funambulus pennantii*	0.019	0.015	-0.600	100.000	1.500	Equal
Australian	*Hemitragus jemlahicus*	0.952	0.817	0.966	100.000	81.700	Equal
Australian	*Isoodon obesulus*	1.000	1.000	0.310	100.000	100.000	Equal
Australian	*Lepus europaeus*	0.737	0.427	0.367	83.700	59.100	5%
Australian	*Macropus agilis*	0.747	0.495	1.000	100.000	49.500	Equal
Australian	*Macropus eugenii*	0.309	0.000	0.318	100.000	0.000	Equal
Australian	*Macropus giganteus*	0.833	0.750	-0.214	100.000	75.000	Equal
Australian	*Macropus parma*	0.500	0.500	0.333	100.000	50.000	Equal
Australian	*Macropus rufogriseus*	0.873	0.699	0.390	95.700	74.300	Equal
Australian	*Mustela erminea*	0.980	0.906	1.000	94.200	96.400	Equal
Australian	*Mustela nivalis*	0.484	0.280	-0.301	100.000	28.000	Equal
Australian	*Mustela putorius*	0.643	0.557	-0.717	66.800	88.800	Equal
Australian	*Odocoileus virginianus*	0.043	0.000	0.018	100.000	0.000	Equal
Australian	*Ornithorhynchus anatinus*	1.000	1.000	-0.268	100.000	100.000	Equal
Australian	*Oryctolagus cuniculus*	0.642	0.461	0.758	57.400	88.700	Realms
Australian	*Petaurus breviceps*	0.853	0.766	-0.323	100.000	76.600	Equal
Australian	*Petrogale lateralis*	0.000	0.000	-1.000	100.000	0.000	Equal
Australian	*Petrogale penicillata*	0.776	0.763	0.143	100.000	76.300	Equal
Australian	*Phascolarctos cinereus*	0.630	0.326	0.775	52.200	80.400	Equal
Australian	*Pseudocheirus peregrinus*	1.000	1.000	0.200	100.000	100.000	Equal
Australian	*Rattus exulans*	0.653	0.351	0.044	49.900	85.200	Equal
Australian	*Rupicapra rupicapra*	0.793	0.622	0.870	67.200	95.000	Equal
Australian	*Rusa timorensis*	0.780	0.478	0.479	82.100	65.700	5%
Australian	*Rusa unicolor*	0.517	0.355	-0.516	87.200	48.300	Equal
Australian	*Sarcophilus harrisii*	1.000	1.000	0.000	100.000	100.000	Equal
Australian	*Tachyglossus aculeatus*	0.857	0.857	0.344	100.000	85.700	Equal
Australian	*Thylogale billardierii*	1.000	1.000	1.000	100.000	100.000	Equal
Australian	*Trichosurus vulpecula*	0.467	0.299	0.222	100.000	29.900	Equal
Australian	*Vombatus ursinus*	0.929	0.857	0.183	100.000	85.700	Equal
Australian	*Vulpes vulpes*	0.798	0.716	0.504	79.600	91.900	Realms
Australian	*Wallabia bicolor*	1.000	1.000	-0.511	100.000	100.000	Equal
Madagascan	*Eulemur fulvus*	0.821	0.786	-0.528	100.000	78.600	Equal
Madagascan	*Eulemur mongoz*	0.446	0.000	-1.000	100.000	0.000	Equal
Madagascan	*Herpestes auropunctatus*	0.147	0.000	-0.500	100.000	0.000	Equal
Madagascan	*Potamochoerus larvatus*	0.371	0.059	-0.361	99.000	6.900	Equal
Madagascan	*Suncus etruscus*	0.469	0.209	-0.556	95.900	25.000	Realms
Madagascan	*Suncus murinus*	0.339	0.016	-0.397	100.000	1.600	Equal
Madagascan	*Tenrec ecaudatus*	0.013	0.000	-0.440	100.000	0.000	Equal
Madagascan	*Viverricula indica*	0.426	0.002	-0.360	100.000	0.200	Equal
Nearctic	*Alces alces*	0.701	0.673	-0.725	100.000	67.300	Equal
Nearctic	*Ammotragus lervia*	0.447	0.097	0.511	98.100	11.600	Equal
Nearctic	*Axis axis*	0.696	0.463	-0.872	86.800	59.500	Equal
Nearctic	*Bison bison*	0.333	0.266	0.035	100.000	26.600	Equal
Nearctic	*Boselaphus tragocamelus*	0.770	0.650	0.273	99.700	65.400	Equal
Nearctic	*Castor canadensis*	0.818	0.674	0.309	100.000	67.400	Equal
Nearctic	*Cervus canadensis*	0.710	0.366	0.343	40.900	95.700	Equal
Nearctic	*Cervus elaphus*	0.910	0.838	-0.083	100.000	83.800	Equal
Nearctic	*Cervus nippon*	0.534	0.293	-0.515	100.000	29.300	Equal

Nearctic	*Chlorocebus sabaeus*	0.623	0.604	-0.400	100.000	60.400	Equal
Nearctic	*Dama dama*	0.699	0.491	0.017	96.300	52.800	Equal
Nearctic	*Dasypus novemcinctus*	0.979	0.958	1.000	100.000	95.800	Equal
Nearctic	*Didelphis virginiana*	0.936	0.756	0.392	96.900	78.800	Equal
Nearctic	*Herpestes auropunctatus*	0.198	0.018	-0.685	100.000	1.800	Equal
Nearctic	*Lepus americanus*	0.787	0.687	0.189	97.900	70.800	Equal
Nearctic	*Lepus arcticus*	0.897	0.846	0.702	100.000	84.600	Equal
Nearctic	*Lepus californicus*	0.738	0.667	0.582	66.700	100.000	Equal
Nearctic	*Lepus europaeus*	0.865	0.614	0.729	95.600	65.800	Equal
Nearctic	*Macaca mulatta*	0.765	0.530	1.000	100.000	53.000	Equal
Nearctic	*Martes americana*	0.764	0.404	0.292	66.200	74.300	Equal
Nearctic	*Martes foina*	0.406	0.094	-0.253	100.000	9.400	Equal
Nearctic	*Microtus californicus*	0.825	0.650	1.000	100.000	65.000	Equal
Nearctic	*Myocastor coypus*	0.491	0.004	0.222	94.400	6.000	Equal
Nearctic	*Myodes gapperi*	0.843	0.684	0.330	79.100	89.300	Equal
Nearctic	*Neovison vison*	0.871	0.710	0.312	94.900	76.100	5%
Nearctic	*Odocoileus hemionus*	0.794	0.690	0.767	96.500	72.500	Equal
Nearctic	*Odocoileus virginianus*	0.644	0.573	-0.860	100.000	57.300	Equal
Nearctic	*Ondatra zibethicus*	0.869	0.659	0.278	86.500	79.300	5%
Nearctic	*Oreamnos americanus*	0.709	0.447	-0.568	85.700	59.100	Equal
Nearctic	*Oryctolagus cuniculus*	0.989	0.980	0.380	100.000	98.000	Equal
Nearctic	*Oryx gazella*	0.516	0.114	0.346	62.500	48.800	Equal
Nearctic	*Ovibos moschatus*	0.698	0.371	-0.472	59.300	77.800	Equal
Nearctic	*Ovis canadensis*	0.826	0.600	0.959	96.200	63.800	Equal
Nearctic	*Ovis orientalis*	0.350	0.180	-0.086	100.000	18.000	Equal
Nearctic	*Peromyscus fraterculus*	1.000	1.000	-0.451	100.000	100.000	Equal
Nearctic	*Peromyscus maniculatus*	0.944	0.804	0.800	89.500	90.900	Equal
Nearctic	*Petrogale penicillata*	0.766	0.579	0.250	100.000	57.900	Equal
Nearctic	*Procyon lotor*	0.849	0.724	0.523	100.000	72.400	Equal
Nearctic	*Rangifer tarandus*	0.241	0.130	0.274	98.000	15.000	Equal
Nearctic	*Rattus exulans*	0.580	0.269	0.089	89.700	37.100	5%
Nearctic	*Rusa unicolor*	0.740	0.575	0.406	89.100	68.400	Equal
Nearctic	*Sciurus aberti*	0.926	0.716	0.748	91.800	79.700	Equal
Nearctic	*Sciurus aureogaster*	1.000	1.000	-0.605	100.000	100.000	Equal
Nearctic	*Sciurus carolinensis*	0.860	0.768	0.279	99.100	77.700	Equal
Nearctic	*Sciurus niger*	0.837	0.544	0.564	94.900	59.500	Equal
Nearctic	*Sorex cinereus*	0.786	0.550	0.664	100.000	55.000	Equal
Nearctic	*Sylvilagus floridanus*	0.771	0.554	-0.435	85.800	69.600	Equal
Nearctic	*Tamias striatus*	0.904	0.881	0.046	100.000	88.100	Equal
Nearctic	*Tamiasciurus hudsonicus*	0.836	0.695	0.557	92.700	76.700	Equal
Nearctic	*Urocitellus parryii*	0.537	0.186	0.024	69.100	49.500	Equal
Nearctic	*Vulpes lagopus*	0.905	0.819	0.520	100.000	81.900	Equal
Nearctic	*Vulpes vulpes*	0.601	0.285	0.449	99.900	28.600	5%
Neotropical	*Antilope cervicapra*	0.648	0.260	0.774	96.400	29.600	Equal
Neotropical	*Axis axis*	0.221	0.000	-0.995	100.000	0.000	Equal
Neotropical	*Bubalus bubalis*	0.660	0.323	0.508	93.600	38.700	Equal
Neotropical	*Callithrix geoffroyi*	0.864	0.660	0.911	88.500	77.500	Equal
Neotropical	*Callithrix jacchus*	0.792	0.462	0.511	89.900	56.300	Equal
Neotropical	*Callithrix penicillata*	0.628	0.205	0.031	60.700	59.800	Equal
Neotropical	*Callosciurus erythraeus*	0.497	0.166	0.790	100.000	16.600	Equal
Neotropical	*Castor canadensis*	0.382	0.140	-0.551	85.200	28.800	Equal
Neotropical	*Cercopithecus mona*	0.357	0.343	-0.366	100.000	34.300	Equal
Neotropical	*Cervus elaphus*	0.588	0.325	0.368	94.600	37.900	Equal
Neotropical	*Chaetophractus villosus*	0.767	0.723	-0.176	100.000	72.300	Equal
Neotropical	*Dama dama*	0.705	0.432	0.119	66.600	76.600	Equal
Neotropical	*Dasyprocta leporina*	0.787	0.565	0.038	100.000	56.500	Equal

Neotropical	*Dasypus novemcinctus*	0.960	0.920	1.000	100.000	92.000	Equal
Neotropical	*Didelphis marsupialis*	0.971	0.938	0.891	98.000	95.700	Equal
Neotropical	*Herpestes auropunctatus*	0.446	0.162	-0.514	84.300	31.900	Equal
Neotropical	*Hippopotamus amphibius*	0.496	0.166	0.495	37.700	78.900	Equal
Neotropical	*Lama guanicoe*	1.000	1.000	-0.600	100.000	100.000	Equal
Neotropical	*Lepus europaeus*	0.646	0.296	-0.344	30.400	99.200	Equal
Neotropical	*Lycalopex griseus*	0.796	0.450	0.280	49.800	95.100	Equal
Neotropical	*Nasua nasua*	0.851	0.851	-0.462	100.000	85.100	Equal
Neotropical	*Neovison vison*	0.247	0.034	-0.836	100.000	3.400	5%
Neotropical	*Odocoileus virginianus*	0.761	0.720	0.407	100.000	72.000	Equal
Neotropical	*Ondatra zibethicus*	0.267	0.064	-0.368	12.400	94.000	5%
Neotropical	*Oryctolagus cuniculus*	0.485	0.119	-0.283	73.500	38.400	5%
Neotropical	*Rangifer tarandus*	0.838	0.611	0.280	78.600	82.500	Equal
Neotropical	*Saguinus oedipus*	1.000	1.000	0.600	100.000	100.000	Equal
Neotropical	*Saimiri sciureus*	0.736	0.583	0.074	100.000	58.300	Equal
Neotropical	*Sapajus apella*	0.250	0.250	-0.550	100.000	25.000	Equal
Neotropical	*Sciurus stramineus*	0.083	0.000	0.568	100.000	0.000	Equal
Neotropical	*Vicugna vicugna*	0.714	0.571	0.716	57.100	100.000	Equal
Oceanian	*Babyrousa babyrussa*	0.568	0.240	-0.403	72.400	51.500	Equal
Oceanian	*Bubalus bubalis*	0.364	0.003	-0.767	0.300	100.000	Realms
Oceanian	*Crocidura maxi*	0.802	0.529	0.587	97.100	55.700	Equal
Oceanian	*Crocidura monticola*	0.668	0.289	-0.170	88.900	40.000	Equal
Oceanian	*Dasyurus hallucatus*	1.000	1.000	1.000	100.000	100.000	Equal
Oceanian	*Dendrolagus matschiei*	0.224	0.096	-0.942	96.000	13.600	Equal
Oceanian	*Herpestes auropunctatus*	0.559	0.219	0.356	78.700	43.200	Equal
Oceanian	*Herpestes fuscus*	0.548	0.133	0.832	94.700	18.600	Equal
Oceanian	*Lepus nigricollis*	0.234	0.186	-0.751	98.200	20.500	Equal
Oceanian	*Macaca fascicularis*	0.341	0.265	0.545	100.000	26.500	Equal
Oceanian	*Macaca nigra*	0.180	0.098	-0.900	100.000	9.800	Equal
Oceanian	*Macropus agilis*	0.542	0.211	-0.400	47.400	73.700	Equal
Oceanian	*Oryctolagus cuniculus*	0.276	0.035	-0.585	100.000	3.500	5%
Oceanian	*Paradoxurus hermaphroditus*	0.496	0.008	-0.571	100.000	0.800	Equal
Oceanian	*Petaurus breviceps*	0.426	0.025	-0.168	100.000	2.500	Equal
Oceanian	*Phalanger orientalis*	0.499	0.223	-0.544	99.700	22.600	Equal
Oceanian	*Rangifer tarandus*	0.263	0.079	-0.643	100.000	7.900	Equal
Oceanian	*Rattus argentiventer*	0.257	0.162	0.509	100.000	16.200	Equal
Oceanian	*Rattus exulans*	0.740	0.534	0.237	83.800	69.500	Realms
Oceanian	*Rattus nitidus*	0.410	0.000	0.101	100.000	0.000	Equal
Oceanian	*Rattus praetor*	0.655	0.402	0.644	90.700	49.500	Equal
Oceanian	*Rattus tanezumi*	0.417	0.000	-0.749	100.000	0.000	5%
Oceanian	*Rusa marianna*	0.699	0.551	0.626	68.800	86.400	Equal
Oceanian	*Rusa timorensis*	0.518	0.170	-0.232	65.000	52.000	5%
Oceanian	*Spilocuscus maculatus*	0.623	0.315	0.575	88.900	42.600	Equal
Oceanian	*Suncus murinus*	0.942	0.770	0.858	77.600	99.400	5%
Oceanian	*Sus celebensis*	0.279	0.003	-0.832	100.000	0.300	Equal
Oceanian	*Tenrec ecaudatus*	0.107	0.013	-0.578	100.000	1.300	Equal
Oceanian	*Thylogale browni*	0.451	0.183	-0.144	99.400	18.900	Equal
Oceanian	*Thylogale brunii*	0.923	0.923	-0.374	100.000	92.300	Equal
Oceanian	*Viverra tangalunga*	0.464	0.010	0.085	90.900	10.100	Equal
Oriental	*Axis axis*	0.519	0.472	-0.052	100.000	47.200	Equal
Oriental	*Axis porcinus*	0.364	0.028	-0.359	57.000	45.800	Equal
Oriental	*Bandicota indica*	0.705	0.352	0.276	54.300	80.900	Equal
Oriental	*Bos javanicus*	0.402	0.003	0.121	100.000	0.400	Equal
Oriental	*Bubalus bubalis*	0.645	0.372	-0.241	85.100	52.100	Equal
Oriental	*Callosciurus finlaysonii*	0.233	0.000	-1.000	100.000	0.000	Equal

Oriental	*Callosciurus notatus*	0.082	0.000	-0.483	100.000	0.000	Equal
Oriental	*Callosciurus prevostii*	0.557	0.197	0.202	95.200	24.500	Equal
Oriental	*Cervus nippon*	0.715	0.698	0.200	100.000	69.800	Equal
Oriental	*Elephas maximus*	0.744	0.534	0.764	87.000	66.400	Equal
Oriental	*Funambulus pennantii*	0.497	0.273	-0.512	64.700	62.600	Equal
Oriental	*Herpestes auropunctatus*	0.046	0.028	-0.828	100.000	2.800	Equal
Oriental	*Hystrix javanica*	0.854	0.680	0.772	92.400	75.500	Equal
Oriental	*Lepus nigricollis*	0.181	0.000	-1.000	100.000	0.000	Equal
Oriental	*Macaca fascicularis*	0.782	0.711	0.117	96.900	74.200	Equal
Oriental	*Macaca leonina*	0.867	0.733	-0.514	100.000	73.300	Equal
Oriental	*Macaca nemestrina*	0.914	0.803	0.783	90.500	89.900	Equal
Oriental	*Manis culionensis*	1.000	1.000	0.618	100.000	100.000	Equal
Oriental	*Muntiacus muntjak*	0.779	0.693	-0.311	83.300	86.000	Equal
Oriental	*Mus caroli*	0.568	0.331	0.694	42.500	90.600	Equal
Oriental	*Mus terricolor*	0.217	0.000	-1.000	100.000	0.000	Equal
Oriental	*Mustela itatsi*	0.476	0.326	-0.552	100.000	32.600	Equal
Oriental	*Paradoxurus hermaphroditus*	0.474	0.020	0.356	10.400	91.600	5%
Oriental	*Phalanger orientalis*	0.194	0.000	-1.000	100.000	0.000	Equal
Oriental	*Rattus argentiventer*	0.850	0.607	0.740	86.900	73.800	Equal
Oriental	*Rattus exulans*	0.894	0.623	0.858	84.700	77.600	Equal
Oriental	*Rattus nitidus*	0.374	0.000	0.081	100.000	0.000	Equal
Oriental	*Rattus tanezumi*	0.292	0.000	-0.169	100.000	0.000	Realms
Oriental	*Rusa timorensis*	0.650	0.302	0.378	66.800	63.400	Equal
Oriental	*Semnopithecus entellus*	0.863	0.821	-0.042	100.000	82.100	Equal
Oriental	*Spilocuscus maculatus*	0.576	0.261	0.690	91.700	34.400	Equal
Oriental	*Suncus murinus*	0.634	0.252	0.836	58.000	67.300	Equal
Oriental	*Sus celebensis*	0.505	0.143	-0.140	24.300	90.000	Equal
Oriental	*Trachypithecus auratus*	0.755	0.458	0.497	90.900	54.900	Equal
Oriental	*Viverra tangalunga*	0.798	0.561	0.329	68.600	87.600	Equal
Oriental	*Viverra zibetha*	0.932	0.799	0.445	98.200	81.700	Equal
Oriental	*Viverricula indica*	0.890	0.774	-0.302	100.000	77.400	Equal
Palearctic	*Ammotragus lervia*	0.437	0.062	0.051	91.300	14.800	Equal
Palearctic	*Apodemus sylvaticus*	0.756	0.568	0.611	78.400	78.400	Equal
Palearctic	*Atelerix algirus*	0.919	0.711	0.518	90.600	80.500	Equal
Palearctic	*Axis axis*	0.522	0.129	-0.200	100.000	12.900	Equal
Palearctic	*Callosciurus erythraeus*	0.832	0.692	0.077	100.000	69.200	Equal
Palearctic	*Callosciurus finlaysonii*	0.767	0.700	-1.000	100.000	70.000	Equal
Palearctic	*Capra aegagrus*	0.727	0.649	-0.051	99.600	65.400	Equal
Palearctic	*Capra ibex*	0.912	0.727	0.722	89.100	83.600	Equal
Palearctic	*Capra sibirica*	0.879	0.831	0.362	100.000	83.100	Equal
Palearctic	*Capreolus capreolus*	0.796	0.773	0.500	100.000	77.300	Equal
Palearctic	*Capreolus pygargus*	0.359	0.000	-0.195	100.000	0.000	Equal
Palearctic	*Castor canadensis*	0.483	0.304	-0.151	99.700	30.700	Equal
Palearctic	*Castor fiber*	0.617	0.486	-0.657	100.000	48.600	Equal
Palearctic	*Cervus canadensis*	0.467	0.162	-0.583	60.000	56.200	Equal
Palearctic	*Cervus elaphus*	0.422	0.351	0.617	100.000	35.100	Equal
Palearctic	*Cervus nippon*	0.794	0.543	0.833	100.000	54.300	Equal
Palearctic	*Crocidura dsinezumi*	0.996	0.988	0.593	100.000	98.800	Equal
Palearctic	*Crocidura pachyura*	0.944	0.830	0.965	96.500	86.600	Equal
Palearctic	*Crocidura russula*	0.874	0.751	0.754	91.500	83.700	Equal
Palearctic	*Crocidura suaveolens*	0.392	0.146	-0.260	100.000	14.600	Equal
Palearctic	*Dama dama*	0.647	0.411	-0.331	96.100	45.000	Equal
Palearctic	*Desmana moschata*	0.826	0.539	0.046	100.000	53.900	Equal
Palearctic	*Eliomys quercinus*	0.854	0.708	1.000	100.000	70.800	Equal
Palearctic	*Erinaceus europaeus*	0.908	0.732	0.537	96.200	77.000	Equal

Palearctic	*Erinaceus roumanicus*	0.284	0.020	-0.511	100.000	2.000	Equal
Palearctic	*Eutamias sibiricus*	0.339	0.000	0.512	100.000	0.000	Equal
Palearctic	*Genetta genetta*	0.540	0.221	0.324	37.600	84.500	Equal
Palearctic	*Glis glis*	0.518	0.356	0.417	94.500	41.100	Equal
Palearctic	*Herpestes auropunctatus*	0.580	0.441	-0.435	58.000	86.100	Equal
Palearctic	*Hydropotes inermis*	0.826	0.677	0.618	98.400	69.200	Equal
Palearctic	*Hystrix cristata*	0.440	0.000	0.361	100.000	0.000	Equal
Palearctic	*Lama glama*	0.500	0.500	-0.393	100.000	50.000	Equal
Palearctic	*Lepus capensis*	0.907	0.858	0.605	100.000	85.800	Equal
Palearctic	*Lepus corsicanus*	0.960	0.823	0.886	97.400	84.900	Equal
Palearctic	*Lepus europaeus*	0.618	0.187	0.808	35.100	83.600	Equal
Palearctic	*Lepus granatensis*	0.910	0.712	0.453	84.400	86.800	Equal
Palearctic	*Lepus timidus*	0.732	0.509	0.531	100.000	50.900	Equal
Palearctic	*Macropus rufogriseus*	0.338	0.329	0.662	100.000	32.900	Equal
Palearctic	*Marmota bobak*	0.866	0.724	0.039	85.700	86.800	Equal
Palearctic	*Marmota marmota*	0.807	0.562	0.625	74.200	82.000	Equal
Palearctic	*Martes foina*	0.296	0.166	-0.369	100.000	16.600	Equal
Palearctic	*Martes martes*	0.410	0.152	0.439	96.000	19.200	Equal
Palearctic	*Martes zibellina*	0.550	0.154	0.726	30.200	85.300	Equal
Palearctic	*Meles meles*	0.535	0.436	-0.462	100.000	43.600	Equal
Palearctic	*Micromys minutus*	0.766	0.582	0.469	100.000	58.200	Equal
Palearctic	*Microtus arvalis*	0.519	0.500	0.605	100.000	50.000	Equal
Palearctic	*Microtus levis*	0.748	0.709	-0.275	100.000	70.900	Equal
Palearctic	*Muntiacus reevesi*	0.859	0.676	0.832	97.700	69.900	Equal
Palearctic	*Mus spretus*	0.970	0.900	0.236	97.300	92.700	Equal
Palearctic	*Mustela erminea*	0.669	0.625	0.561	100.000	62.500	Equal
Palearctic	*Mustela lutreola*	0.784	0.736	-0.670	100.000	73.600	Equal
Palearctic	*Mustela nivalis*	0.622	0.367	0.186	74.000	62.800	5%
Palearctic	*Mustela putorius*	0.643	0.342	-0.480	53.700	80.500	Equal
Palearctic	*Myocastor coypus*	0.519	0.038	0.084	98.700	5.100	Equal
Palearctic	*Myodes glareolus*	0.870	0.741	0.953	93.900	80.200	Equal
Palearctic	*Myodes rutilus*	0.359	0.348	0.105	100.000	34.800	Equal
Palearctic	*Nasua nasua*	0.489	0.000	-1.000	100.000	0.000	Equal
Palearctic	*Neovison vison*	0.639	0.212	0.359	52.100	69.000	Equal
Palearctic	*Nyctereutes procyonoides*	0.604	0.195	0.208	45.500	74.000	Equal
Palearctic	*Odocoileus virginianus*	0.705	0.613	0.604	98.300	63.000	Equal
Palearctic	*Ondatra zibethicus*	0.624	0.224	0.561	41.400	81.100	5%
Palearctic	*Oryctolagus cuniculus*	0.725	0.500	-0.280	72.400	77.600	Equal
Palearctic	*Ovibos moschatus*	0.767	0.540	0.354	78.400	75.600	Equal
Palearctic	*Ovis orientalis*	0.285	0.087	0.013	100.000	8.700	Equal
Palearctic	*Procyon lotor*	0.692	0.465	0.596	91.900	54.500	Equal
Palearctic	*Rangifer tarandus*	0.435	0.285	0.589	96.700	31.900	Equal
Palearctic	*Rupicapra rupicapra*	0.716	0.457	0.575	63.500	82.200	Equal
Palearctic	*Sciurus anomalus*	0.667	0.667	0.605	100.000	66.700	Equal
Palearctic	*Sciurus carolinensis*	0.545	0.173	0.464	100.000	17.300	Equal
Palearctic	*Sciurus vulgaris*	0.837	0.560	0.811	84.800	71.200	Equal
Palearctic	*Suncus etruscus*	0.978	0.902	0.810	95.800	94.400	Equal
Palearctic	*Sylvilagus floridanus*	0.654	0.329	-0.503	79.100	53.800	Equal
Palearctic	*Vulpes lagopus*	0.897	0.838	0.596	100.000	83.800	Equal
Palearctic	*Vulpes vulpes*	0.589	0.266	0.491	98.100	28.500	5%
Panamanian	*Antilope cervicapra*	0.584	0.427	0.594	100.000	42.700	5%
Panamanian	*Boselaphus tragocamelus*	0.756	0.551	0.555	73.700	81.400	Equal
Panamanian	*Bubalus bubalis*	0.423	0.234	0.497	93.800	29.600	5%
Panamanian	*Chlorocebus sabaeus*	0.047	0.000	-1.000	100.000	0.000	Equal
Panamanian	*Cuniculus paca*	0.551	0.190	0.832	97.500	21.500	Equal
Panamanian	*Dama dama*	0.443	0.202	-0.286	100.000	20.200	5%

Panamanian	[illegible]	[illegible]	[illegible]	[illegible]	[illegible]	[illegible]	[illegible]
Panamanian	[illegible]	[illegible]	[illegible]	[illegible]	[illegible]	[illegible]	[illegible]
Panamanian	[illegible]	[illegible]	[illegible]	[illegible]	[illegible]	[illegible]	[illegible]
Panamanian	[illegible]	[illegible]	[illegible]	[illegible]	[illegible]	[illegible]	[illegible]
Panamanian	[illegible]	[illegible]	[illegible]	[illegible]	[illegible]	[illegible]	[illegible]
Panamanian	[illegible]	[illegible]	[illegible]	[illegible]	[illegible]	[illegible]	[illegible]
Panamanian	[illegible]	[illegible]	[illegible]	[illegible]	[illegible]	[illegible]	[illegible]
Panamanian	[illegible]	[illegible]	[illegible]	[illegible]	[illegible]	[illegible]	[illegible]
Panamanian	[illegible]	[illegible]	[illegible]	[illegible]	[illegible]	[illegible]	[illegible]
Panamanian	[illegible]	[illegible]	[illegible]	[illegible]	[illegible]	[illegible]	[illegible]
Panamanian	[illegible]	[illegible]	[illegible]	[illegible]	[illegible]	[illegible]	[illegible]
Panamanian	[illegible]	[illegible]	[illegible]	[illegible]	[illegible]	[illegible]	[illegible]
Panamanian	[illegible]	[illegible]	[illegible]	[illegible]	[illegible]	[illegible]	[illegible]
Panamanian	[illegible]	[illegible]	[illegible]	[illegible]	[illegible]	[illegible]	[illegible]
Panamanian	[illegible]	[illegible]	[illegible]	[illegible]	[illegible]	[illegible]	[illegible]
Panamanian	[illegible]	[illegible]	[illegible]	[illegible]	[illegible]	[illegible]	[illegible]
Panamanian	[illegible]	[illegible]	[illegible]	[illegible]	[illegible]	[illegible]	[illegible]
Saharo-Arabian	[illegible]	[illegible]	[illegible]	[illegible]	[illegible]	[illegible]	[illegible]
Saharo-Arabian	[illegible]	[illegible]	[illegible]	[illegible]	[illegible]	[illegible]	[illegible]
Saharo-Arabian	[illegible]	[illegible]	[illegible]	[illegible]	[illegible]	[illegible]	[illegible]
Saharo-Arabian	[illegible]	[illegible]	[illegible]	[illegible]	[illegible]	[illegible]	[illegible]
Saharo-Arabian	[illegible]	[illegible]	[illegible]	[illegible]	[illegible]	[illegible]	[illegible]
Saharo-Arabian	[illegible]	[illegible]	[illegible]	[illegible]	[illegible]	[illegible]	[illegible]
Saharo-Arabian	[illegible]	[illegible]	[illegible]	[illegible]	[illegible]	[illegible]	[illegible]
Saharo-Arabian	[illegible]	[illegible]	[illegible]	[illegible]	[illegible]	[illegible]	[illegible]
Saharo-Arabian	[illegible]	[illegible]	[illegible]	[illegible]	[illegible]	[illegible]	[illegible]
Saharo-Arabian	[illegible]	[illegible]	[illegible]	[illegible]	[illegible]	[illegible]	[illegible]
Saharo-Arabian	*Oryctolagus cuniculus*	0.645	0.435	-0.350	72.500	71.000	5%
Saharo-Arabian	*Ovis orientalis*	0.437	0.005	-0.179	11.000	89.500	Equal
Saharo-Arabian	*Suncus etruscus*	0.973	0.888	0.800	95.400	93.400	Equal
Saharo-Arabian	*Suncus murinus*	0.606	0.229	0.267	46.900	76.000	Equal
Saharo-Arabian	*Vulpes vulpes*	0.584	0.293	0.491	98.100	31.200	5%
Sino-Japanese	*Callosciurus erythraeus*	0.632	0.226	0.595	78.000	44.600	Equal
Sino-Japanese	*Callosciurus finlaysonii*	0.699	0.398	-0.400	63.200	76.700	Equal
Sino-Japanese	*Crocidura dsinezumi*	1.000	0.994	0.593	100.000	99.400	Equal
Sino-Japanese	*Eutamias sibiricus*	0.373	0.053	0.010	100.000	5.300	Equal
Sino-Japanese	*Herpestes auropunctatus*	0.029	0.000	-0.200	100.000	0.000	5%
Sino-Japanese	*Macaca cyclopis*	0.797	0.591	0.696	73.200	85.900	Equal
Sino-Japanese	*Macaca fascicularis*	0.925	0.916	0.248	100.000	91.600	Equal
Sino-Japanese	*Macaca mulatta*	0.366	0.110	0.117	100.000	11.000	Equal
Sino-Japanese	*Martes melampus*	0.998	0.988	0.393	100.000	98.800	Equal
Sino-Japanese	*Muntiacus reevesi*	0.547	0.093	1.000	100.000	9.300	Equal
Sino-Japanese	*Mustela itatsi*	0.890	0.788	-0.007	90.700	88.200	Equal
Sino-Japanese	*Mustela lutreola*	0.782	0.770	-0.670	100.000	77.000	Equal
Sino-Japanese	*Mustela sibirica*	0.399	0.002	-0.237	91.000	9.200	5%
Sino-Japanese	*Myocastor coypus*	0.325	0.000	-0.824	100.000	0.000	Equal
Sino-Japanese	*Neovison vison*	0.674	0.286	0.325	50.200	78.300	5%
Sino-Japanese	*Nyctereutes procyonoides*	0.000	0.000	-1.000	100.000	0.000	5%
Sino-Japanese	*Ondatra zibethicus*	0.223	0.001	-0.959	100.000	0.100	5%
Sino-Japanese	*Oryctolagus cuniculus*	0.357	0.006	0.016	42.900	57.700	5%
Sino-Japanese	*Paguma larvata*	0.986	0.929	0.840	97.600	95.300	Equal
Sino-Japanese	*Procyon lotor*	0.851	0.639	0.780	100.000	63.900	Equal
Sino-Japanese	*Sciurus vulgaris*	0.155	0.026	-0.529	100.000	2.600	Equal
Sino-Japanese	*Suncus murinus*	0.698	0.466	-0.135	94.300	52.300	5%

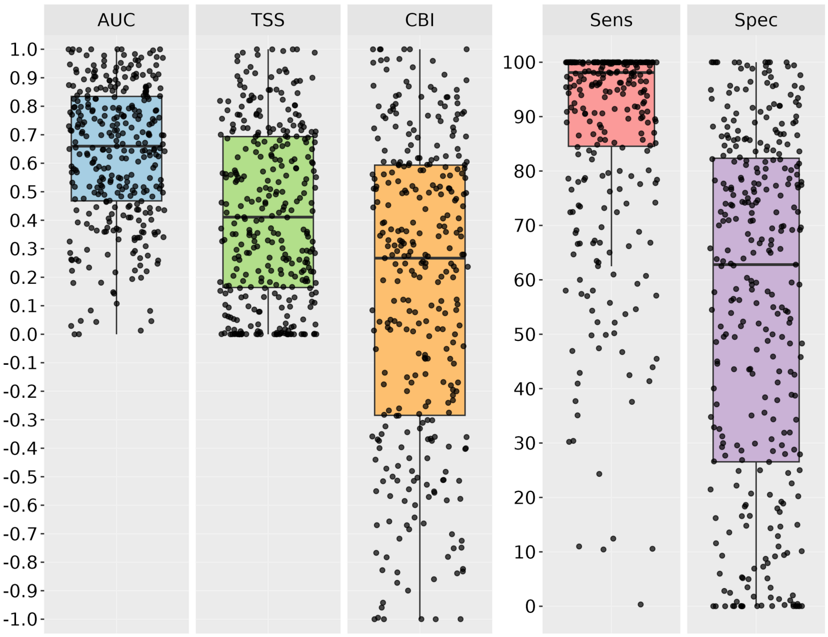

Figure S2.1: Area Under the receiver operating characteristic Curve (AUC), maximized True Skill Statistic (TSS), Continuous Boyce Index (CBI), Sensitivity (Sens) and Specificity (Spec) of Native-SDMs for each species in each realm.

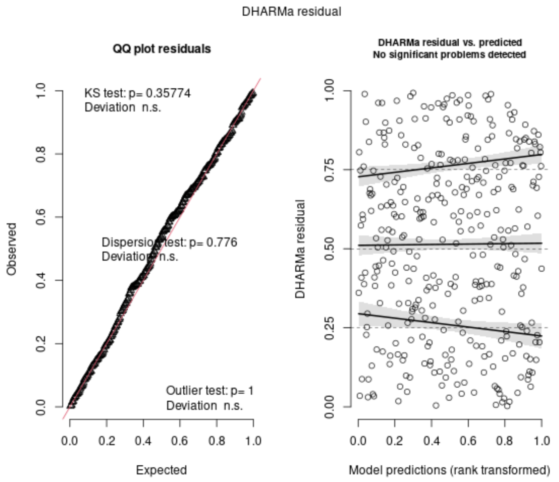

Figure S2.2: DHARMa tests and diagnostic plots for the global niche-expansion GLMM.

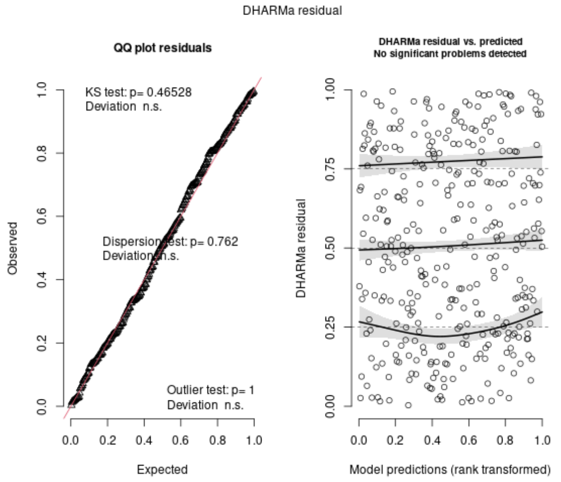

Figure S2.3: DHARMa tests and diagnostic plots for the best-supported niche-expansion GLMM number 1369.

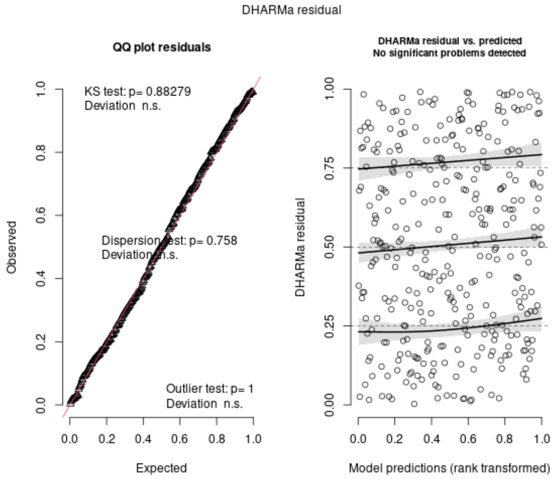

Figure S2.4: DHARMa tests and diagnostic plots for the best-supported niche-expansion GLMM number 1400.

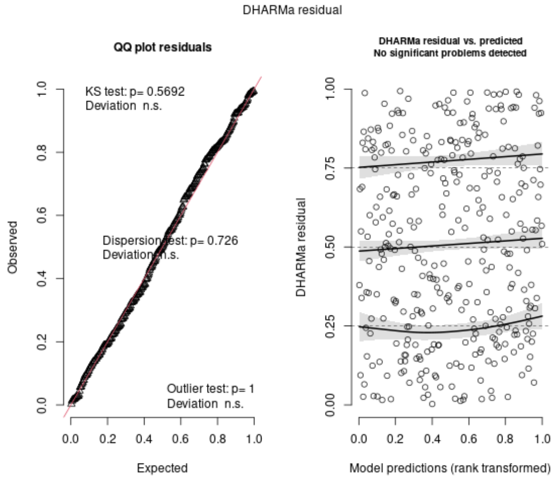

Figure S2.5: DHARMa tests and diagnostic plots for the best-supported niche-expansion GLMM number 1880.

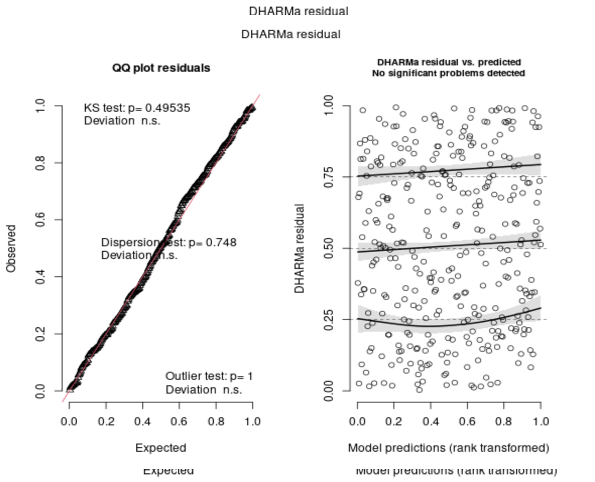

Figure S2.6: DHARMa tests and diagnostic plots for the best-supported niche-expansion GLMM number 1368.

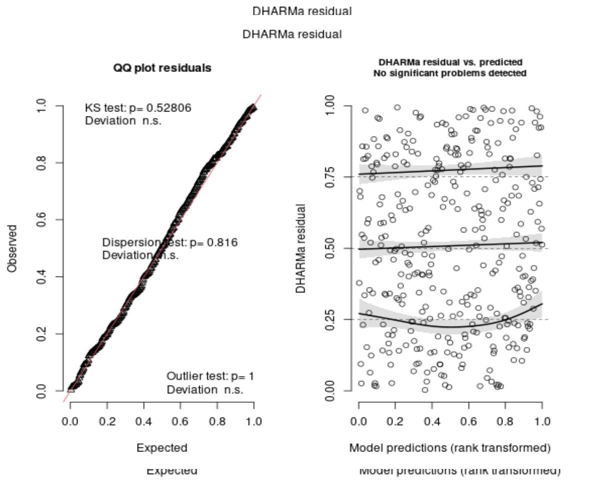

Figure S2.7: DHARMa tests and diagnostic plots for the best-supported niche-expansion GLMM number 345.

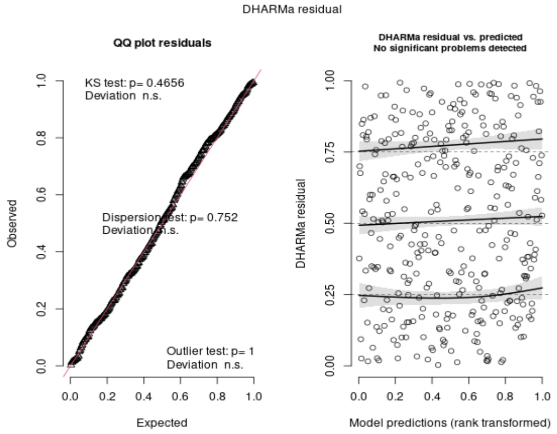

Figure S2.8: DHARMa tests and diagnostic plots for the best-supported niche-expansion GLMM number 344.

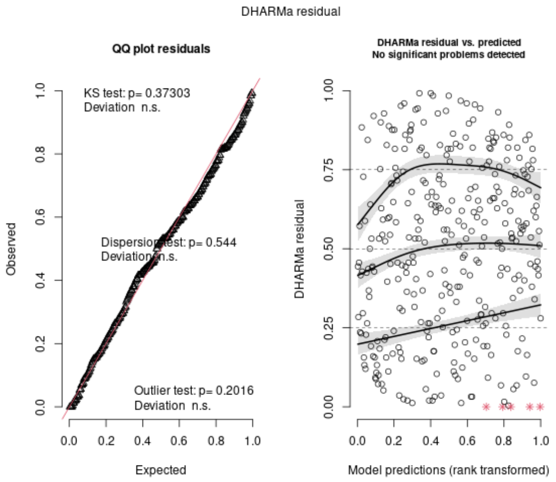

Figure S2.9: DHARMa tests and diagnostic plots for the global niche-unfilling GLMM.

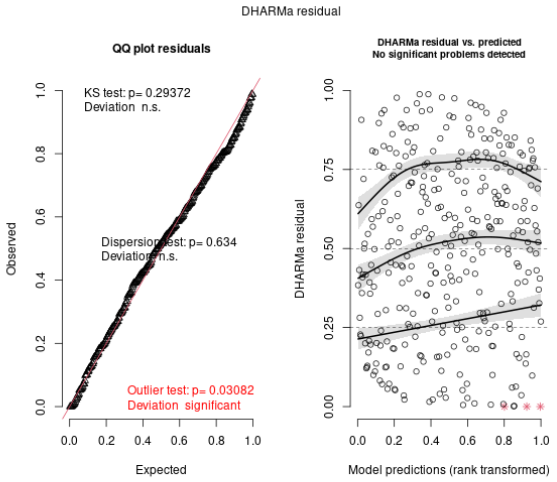

Figure S2.10: DHARMa tests and diagnostic plots for the best-supported niche-unfilling GLMM number 697.

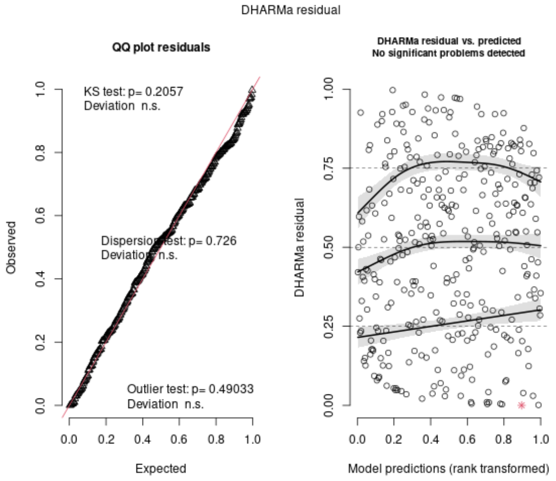

Figure S2.11: DHARMa tests and diagnostic plots for the best-supported niche-unfilling GLMM number 569.

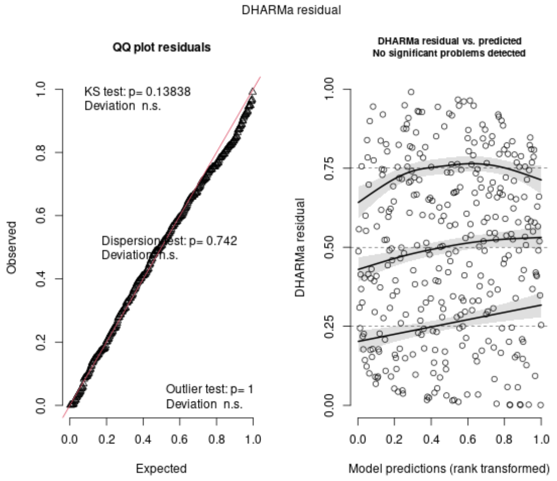

Figure S2.12: DHARMa tests and diagnostic plots for the best-supported niche-unfilling GLMM number 696.

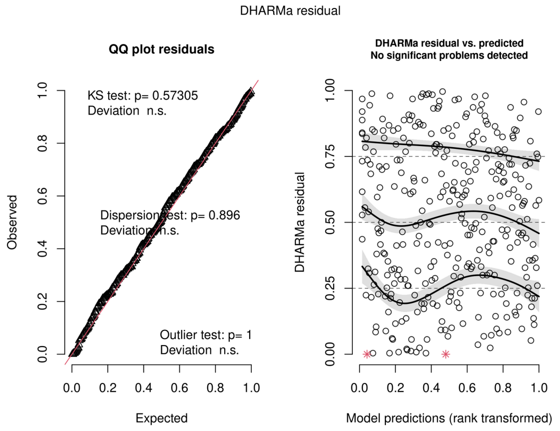

Figure S2.13: DHARMa diagnostics tests and plots for the LMM of the Area Under the receiver operating characteristic Curve.

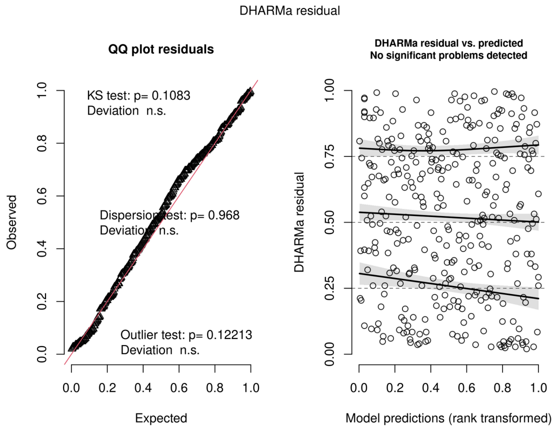

Figure S2.14: DHARMa diagnostics tests and plots for the LMM of the maximized True Skill Statistic.

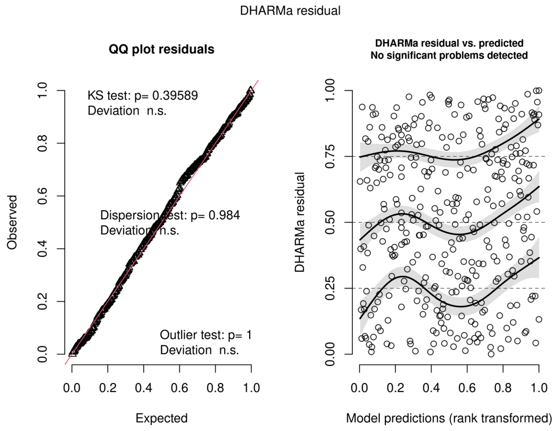

Figure S2.15: DHARMa diagnostics tests and plots for the GLMM of Sensitivity.

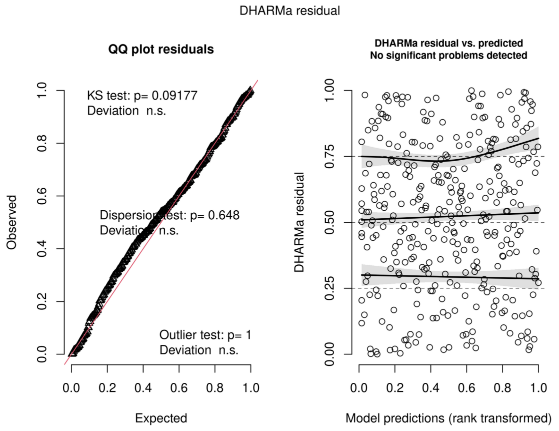

Figure S2.16: DHARMa diagnostics tests and plots for the GLMM of Specificity.

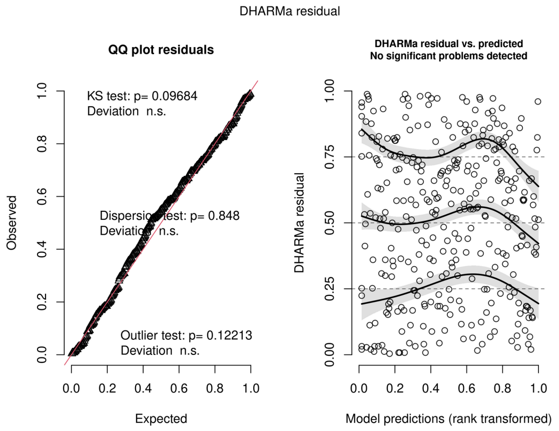

Figure S2.17: DHARMa diagnostics tests and plots for the LMM of the Continuous Boyce Index.

Table S2.3: Comparison of niche-expansion models, ranked by Akaike's Information Criterion corrected for small sample sizes (AICc). Only models with a ΔAICc < 7 are reported, along with their corresponding Bayesian Information Criterion (BIC), log-likelihood (logLik), and residual degrees of freedom (RDF).

Model	AICc	BIC	logLik	RDF	ΔAICc
1369	342.52	373.08	-163.26	330	0
1400	342.98	373.54	-163.49	330	0.46
1880	343.13	373.69	-163.57	330	0.61
1368	343.35	370.09	-164.67	331	0.73
345	343.86	370.6	-164.93	331	1.24
344	344.14	367.06	-166.07	332	1.44
1385	348.53	379.09	-166.27	330	6.01
1353	349.3	376.04	-167.65	331	6.69

Table S2.4: Comparison of niche-unfilling models, ranked by Akaike's Information Criterion corrected for small sample sizes (AICc). Only models with a ΔAICc < 7 are reported, along with their corresponding Bayesian Information Criterion (BIC), log-likelihood (logLik), and residual degrees of freedom (RDF).

Model	AICc	BIC	logLik	RDF	ΔAICc
697	-293.95	-255.75	156.97	330	0
569	-292.04	-257.66	155.02	331	1.79
696	-290.28	-255.9	154.14	331	3.54
689	-287.88	-253.5	152.94	331	5.95
184	-287.51	-256.95	151.76	332	6.2
561	-287.16	-256.6	151.58	332	6.55

Table S2.5: Fixed Variable name, Estimate, Standard Error (Std. Error), 95% Confidence Interval (CI), z-value, p-value, and Significance levels (Sign) of the niche-expansion models with a ΔAICc < 7.

Model	Variable	Estimate	Std. Error	CI low	CI high	z.value	p.value	Sign
1369	Intercept	-0.9	0.3	-1.48	-0.32	-3.05	0	**
1369	Alien insularity	-0.57	0.34	-1.24	0.09	-1.68	0.09	
1369	Human disturbance	-0.62	0.18	-0.97	-0.26	-3.4	0	***
1369	Introduction effort	0.49	0.17	0.15	0.83	2.85	0	**
1369	Native range size	-1.11	0.22	-1.53	-0.68	-5.15	0	***
1369	Community similarity	-0.67	0.17	-1.02	-0.33	-3.86	0	***
1369	Specialization index	-0.32	0.18	-0.68	0.03	-1.77	0.08	
1400	Intercept	-1.27	0.22	-1.71	-0.83	-5.65	0	***
1400	Human disturbance	-0.53	0.19	-0.89	-0.17	-2.88	0	**
1400	Introduction effort	0.53	0.17	0.2	0.87	3.1	0	**
1400	Native range loss	0.27	0.18	-0.08	0.63	1.53	0.13	
1400	Native range size	-1	0.22	-1.42	-0.57	-4.6	0	***
1400	Community similarity	-0.67	0.18	-1.01	-0.32	-3.8	0	***
1400	Specialization index	-0.32	0.19	-0.68	0.04	-1.73	0.08	
1880	Intercept	-1.3	0.23	-1.75	-0.85	-5.63	0	***
1880	Human disturbance	-0.58	0.18	-0.93	-0.22	-3.16	0	**
1880	Introduction effort	0.62	0.18	0.27	0.97	3.45	0	***
1880	Native range size	-1.1	0.22	-1.53	-0.67	-4.99	0	***
1880	Community similarity	-0.68	0.18	-1.02	-0.33	-3.82	0	***
1880	Residence time	-0.26	0.18	-0.6	0.09	-1.45	0.15	
1880	Specialization index	-0.32	0.19	-0.69	0.05	-1.68	0.09	
1368	Intercept	-1.27	0.23	-1.71	-0.83	-5.66	0	***
1368	Human disturbance	-0.59	0.18	-0.95	-0.24	-3.25	0	**
1368	Introduction effort	0.56	0.17	0.23	0.9	3.28	0	**
1368	Native range size	-1.08	0.22	-1.5	-0.65	-4.99	0	***
1368	Community similarity	-0.68	0.18	-1.02	-0.34	-3.88	0	***
1368	Specialization index	-0.3	0.18	-0.66	0.06	-1.62	0.1	

345	Intercept	-0.94	0.29	-1.52	-0.37	-3.22	0	**
345	Alien insularity	-0.51	0.34	-1.17	0.15	-1.52	0.13	
345	Human disturbance	-0.59	0.18	-0.94	-0.24	-3.33	0	***
345	Introduction effort	0.51	0.17	0.18	0.84	2.99	0	**
345	Native range size	-1.03	0.21	-1.44	-0.63	-5.04	0	***
345	Community similarity	-0.7	0.17	-1.04	-0.36	-4.05	0	***
344	Intercept	-1.27	0.22	-1.71	-0.83	-5.68	0	***
344	Human disturbance	-0.57	0.18	-0.92	-0.22	-3.2	0	**
344	Introduction effort	0.57	0.17	0.24	0.9	3.37	0	***
344	Native range size	-1.01	0.21	-1.42	-0.61	-4.92	0	***
344	Community similarity	-0.7	0.17	-1.04	-0.36	-4.05	0	***
1385	Intercept	-0.78	0.28	-1.34	-0.23	-2.76	0.01	**
1385	Alien insularity	-0.74	0.33	-1.39	-0.08	-2.21	0.03	*
1385	Human disturbance	-0.59	0.18	-0.95	-0.24	-3.27	0	**
1385	Native range loss	0.29	0.18	-0.06	0.64	1.65	0.1	
1385	Native range size	-0.96	0.21	-1.37	-0.56	-4.63	0	***
1385	Community similarity	-0.65	0.17	-0.98	-0.31	-3.79	0	***
1385	Specialization index	-0.38	0.18	-0.74	-0.02	-2.08	0.04	*
1353	Intercept	-0.75	0.28	-1.3	-0.19	-2.65	0.01	**
1353	Alien insularity	-0.8	0.33	-1.45	-0.15	-2.42	0.02	*
1353	Human disturbance	-0.66	0.18	-1.01	-0.31	-3.72	0	***
1353	Native range size	-1.04	0.21	-1.45	-0.64	-5.06	0	***
1353	Community similarity	-0.66	0.17	-1	-0.33	-3.89	0	***
1353	Specialization index	-0.36	0.18	-0.71	-0.01	-1.99	0.05	*

Table S2.6: Fixed Variable name, Estimate, Standard Error (Std. Error), 95% Confidence Interval (CI), z-value, p-value, and Significance levels of the niche-unfilling models with a ΔAICc < 7.

**Model**	**Variable**	**Estimate**	**Std. Error**	**CI low**	**CI high**	**z.value**	**p.value**	**Sign**
697	Intercept	-0.182	0.25	-0.67	0.31	-0.73	4.645e-01	
697	Alien insularity	0.513	0.22	0.09	0.93	2.38	1.717e-02	*
697	Human disturbance	0.222	0.08	0.07	0.38	2.85	4.330e-03	**
697	Introduction effort	-0.447	0.07	-0.59	-0.3	-6.04	1.579e-09	***
697	Native range loss	0.325	0.09	0.16	0.49	3.82	1.336e-04	***
697	Native-mammal richness	-0.191	0.1	-0.38	0	-1.98	4.816e-02	*
697	Residence time	-0.247	0.08	-0.41	-0.08	-2.93	3.369e-03	**
569	Intercept	-0.353	0.23	-0.8	0.1	-1.54	1.248e-01	
569	Alien insularity	0.777	0.17	0.44	1.11	4.56	5.161e-06	***
569	Human disturbance	0.204	0.08	0.05	0.36	2.63	8.609e-03	**
569	Introduction effort	-0.442	0.07	-0.59	-0.3	-5.96	2.602e-09	***
569	Native range loss	0.319	0.09	0.15	0.49	3.75	1.796e-04	***
569	Residence time	-0.290	0.08	-0.45	-0.13	-3.57	3.539e-04	***
696	Intercept	0.170	0.21	-0.24	0.58	0.82	4.104e-01	
696	Human disturbance	0.212	0.08	0.06	0.37	2.7	6.843e-03	**
696	Introduction effort	-0.485	0.07	-0.63	-0.34	-6.68	2.350e-11	***
696	Native range loss	0.323	0.09	0.16	0.49	3.78	1.599e-04	***
696	Native-mammal richness	-0.333	0.08	-0.48	-0.18	-4.34	1.452e-05	***
696	Residence time	-0.172	0.08	-0.33	-0.02	-2.18	2.904e-02	*
689	Intercept	-0.112	0.27	-0.64	0.42	-0.42	6.775e-01	
689	Alien insularity	0.480	0.22	0.05	0.91	2.21	2.736e-02	*
689	Introduction effort	-0.459	0.08	-0.61	-0.31	-6.15	7.862e-10	***
689	Native range loss	0.287	0.09	0.12	0.45	3.36	7.760e-04	***
689	Native-mammal richness	-0.158	0.1	-0.35	0.03	-1.65	1.001e-01	
689	Residence time	-0.234	0.09	-0.4	-0.07	-2.77	5.648e-03	**
184	Intercept	0.154	0.2	-0.24	0.55	0.77	4.418e-01	
184	Human disturbance	0.206	0.08	0.05	0.36	2.63	8.518e-03	**

184	Introduction effort	-0.510	0.07	-0.65	-0.37	-7.11	1.161e-12	***
184	Native range loss	0.332	0.09	0.17	0.5	3.89	9.953e-05	***
184	Native-mammal richness	-0.339	0.08	-0.49	-0.19	-4.41	1.027e-05	***
561	Intercept	-0.264	0.25	-0.75	0.22	-1.06	2.885e-01	
561	Alien insularity	0.708	0.17	0.38	1.04	4.2	2.621e-05	***
561	Introduction effort	-0.455	0.08	-0.6	-0.31	-6.1	1.094e-09	***
561	Native range loss	0.284	0.09	0.12	0.45	3.33	8.823e-04	***
561	Residence time	-0.272	0.08	-0.43	-0.11	-3.34	8.448e-04	***

Table S2.7: Random-effect variance and standard deviation of the niche-expansion models with a ΔAICc < 7.

**Model**	**Group**	**Variance**	**Std. Dev**
1369	Binomial	0.958	0.979
1400	Binomial	1.030	1.015
1880	Binomial	1.130	1.063
1368	Binomial	1.049	1.024
345	Binomial	0.952	0.976
344	Binomial	1.028	1.014
1385	Binomial	0.981	0.990
1353	Binomial	0.990	0.995

Table S2.8: Random-effect variance and standard deviation of the niche-unfilling models with a ΔAICc < 7.

**Model**	**Group**	**Variance**	**Std. Dev**
697	Realm	0.202	0.450
697	Order	0.122	0.350
569	Realm	0.190	0.436
569	Order	0.120	0.347
696	Realm	0.208	0.456
696	Order	0.135	0.367
689	Realm	0.282	0.531
689	Order	0.152	0.390
184	Realm	0.193	0.439
184	Order	0.123	0.350
561	Realm	0.261	0.511
561	Order	0.148	0.384

Table S2.9: Fixed Variable name, Estimate, adjusted Standard Error (Std. Error) and 95% Confidence Interval (CI) of the full-averaged niche-expansion model.

**Variable**	**Estimate**	**Std. Error**	**CI low**	**CI high**	**z.value**
Intercept	-1.146	0.308	-1.751	-0.542	3.717
Alien insularity	-0.200	0.336	-0.858	0.458	0.595
Human disturbance	-0.581	0.184	-0.942	-0.220	3.155
Introduction effort	0.543	0.179	0.193	0.893	3.043
Native range size	-1.060	0.219	-1.490	-0.631	4.837
Community similarity	-0.680	0.176	-1.025	-0.336	3.866
Specialization Index	-0.240	0.211	-0.654	0.174	1.137
Native range loss	0.051	0.132	-0.208	0.311	0.388
Residence time	-0.044	0.121	-0.282	0.194	0.364

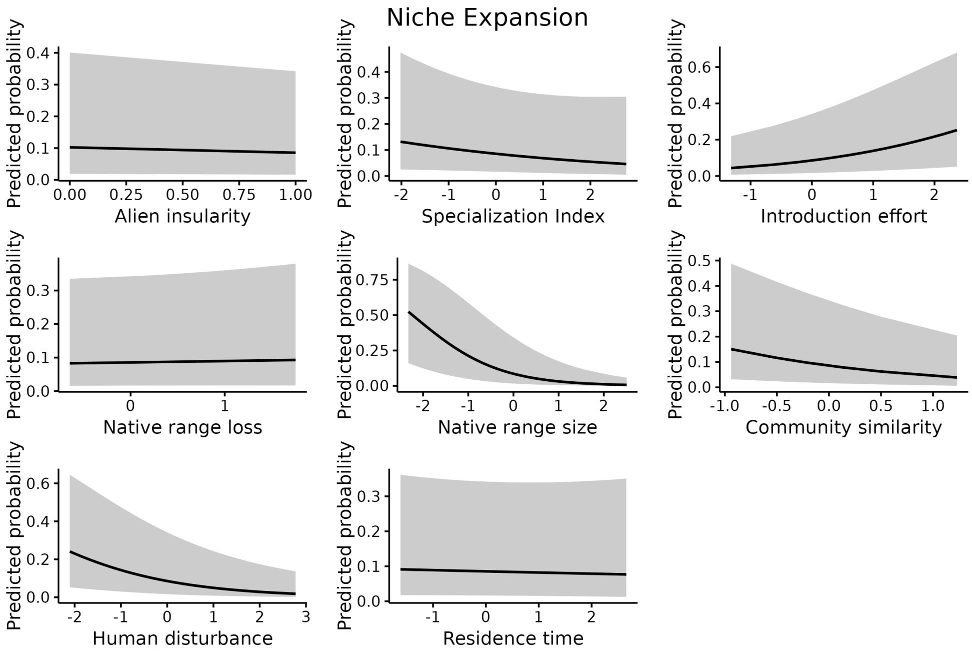

Figure S2.18: Marginal effects of six variables on the probability of niche expansion from the full-averaged GLMM. Each panel shows the predicted probability (solid line) and its 95% confidence ribbon as a function of one standardized predictor while holding all others at their mean.

Table S2.10: Fixed Variable name, Estimate, adjusted Standard Error (Std. Error) and 95% Confidence Interval (CI) of the full-averaged niche-unfilling model.

**Variable**	**Estimate**	**Std. Error**	**CI low**	**CI high**	**z.value**
Intercept	-0.188	0.281	-0.738	0.362	0.671
Alien insularity	0.526	0.289	-0.040	1.091	1.822
Human disturbance	0.216	0.078	0.062	0.370	2.754
Introduction effort	-0.450	0.075	-0.597	-0.302	5.978
Native range loss	0.323	0.085	0.156	0.491	3.780
Native-mammal richness	-0.157	0.130	-0.412	0.099	1.201
Residence time	-0.250	0.089	-0.425	-0.075	2.795

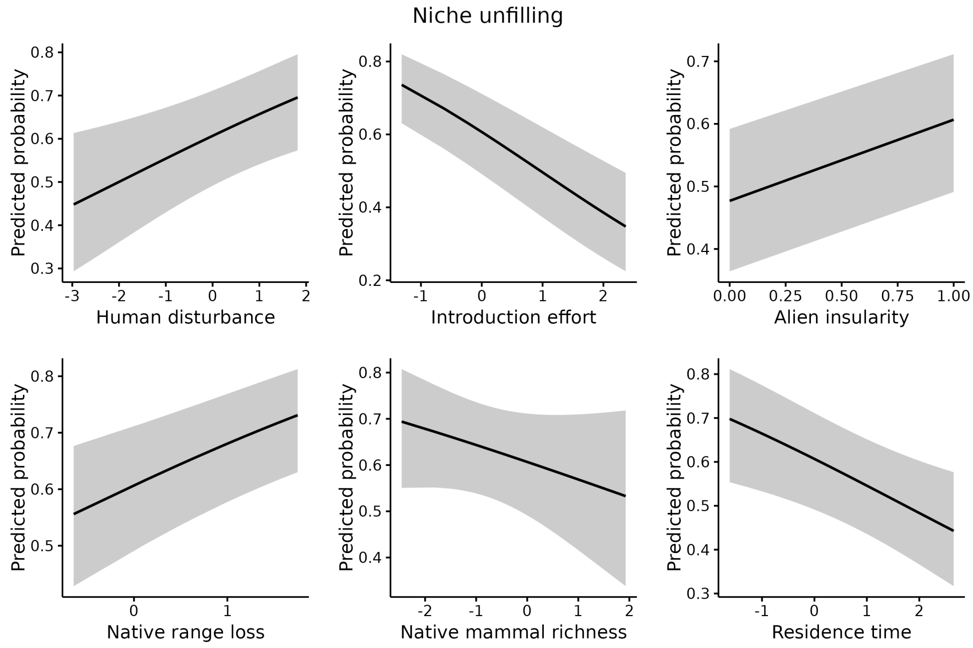

Figure S2.19: Marginal effects of six variables on the probability of niche unfilling from the full-averaged GLMM. Each panel shows the predicted probability (solid line) and its 95% confidence ribbon as a function of one standardized predictor while holding all others at their mean.

Table S2.11: Fixed Variable name, Estimate, Standard Error (Std. Error), 95% Confidence Interval (CI), z-value, p-value, and Significance levels of the Native-SDMs transferability models.

Model	Variable	Estimate	Std.Error	CI low	CI high	z.value	p.value	Significance
AUC	Intercept	1	0.06	0.88	1.12	16.35	4.697e-60	***
AUC	Expansion	-0.18	0.06	-0.3	-0.06	-3.02	2.509e-03	**
AUC	Unfilling	-0.07	0.05	-0.16	0.02	-1.44	1.500e-01	
Boyce	Intercept	0.97	0.06	0.86	1.09	16.36	3.539e-60	***
Boyce	Expansion	-0.2	0.07	-0.34	-0.06	-2.8	5.192e-03	**
Boyce	Unfilling	-0.13	0.06	-0.25	-0.02	-2.39	1.684e-02	*
Sens	Intercept	0.26	0.26	-0.24	0.77	1.03	3.020e-01	
Sens	Expansion	-1.18	0.46	-2.07	-0.28	-2.58	1.003e-02	*
Sens	Unfilling	1.62	0.38	0.88	2.37	4.26	2.027e-05	***
Spec	Intercept	1.71	0.56	0.62	2.81	3.07	2.174e-03	**
Spec	Expansion	-0.25	0.55	-1.32	0.83	-0.45	6.531e-01	
Spec	Unfilling	-0.85	0.48	-1.78	0.08	-1.78	7.461e-02	
TSS	Intercept	0.64	0.09	0.47	0.8	7.51	5.934e-14	***
TSS	Expansion	-0.28	0.08	-0.44	-0.13	-3.53	4.212e-04	***
TSS	Unfilling	0.08	0.06	-0.04	0.21	1.35	1.787e-01	

Table S2.12: Random effects Standard Deviation and Variance for the Native-SDMs transferability models.

Model	Group	Random Effect	Std. Deviation	Variance
AUC	Realm	0.01	0.12	0.028
AUC	Sample size	0.01	0.07	0.010
CBI	Realm	0.01	0.11	0.128
CBI	Sample size	0	0.06	0.014
Spec	Realm	1.45	1.2	0.005
Spec	Sample size	0.18	0.43	0.073
TSS	Realm	0.03	0.17	0.011
TSS	Sample size	0.01	0.11	0.003

Table S2.13: The Akaike's Information Criterion corrected for small sample sizes (AICc), Bayesian Information Criterion (BIC), log-likelihood (logLik), residual standard deviation (sigma) and residual degrees of freedom (RDF) for the Native-SDMs transferability models.

Model	AICc	BIC	logLik	sigma	RDF
AUC	109.202	132.087	-48.601	0.269	329
CBI	219.923	242.808	-103.961	0.321	329
Sensitivity	360.051	371.494	-177.026	1.000	330
Specificity	354.841	373.911	-172.420	1.000	330
TSS	301.474	324.359	-144.737	0.358	329

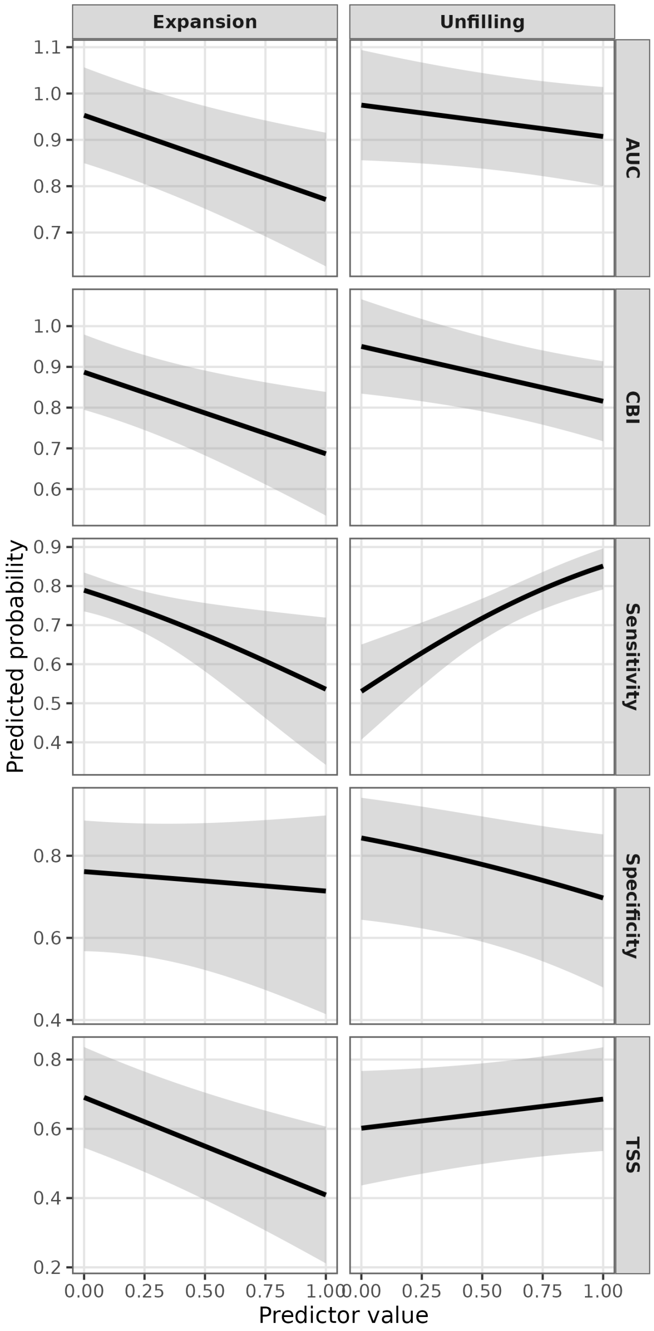

Figure S2.20: Marginal effects of six variables on the probability of Native-SDMs AUC, TSS, CBI, Sensitivity and Specificity from (G)LMMs. Each panel shows the predicted probability (solid line) and its 95% confidence ribbon as a function of one standardized predictor while holding all others at their mean.
